# Genome sequencing and *de novo* assembly elucidate the genetic basis of grain quality and fragrance in heirloom rice cultivar *Mushk Budji*

**DOI:** 10.3389/fpls.2026.1896265

**Published:** 2026-10-14

**Authors:** Raheel Shafeeq Khan, Noor-ul-Ain, V. Shasi Kiran Reddy, Ishrat Rafiq, Gazala H. Khan, Fayaz Mohiddin, Sajad M. Zargar, Syed Zameer Hussain, Bashir A. Alie, Najeeb-ul-Rehman Sofi, Asif Bashir Shikari

**Affiliations:** 1 Division of Genetics & Plant Breeding, Sher-e-Kashmir University of Agricultural Sciences & Technology of Kashmir, Wadura, J&K, India; 2 Mountain Research Centre for Field Crops, Sher-e Kashmir University of Agricultural Sciences and Technology of Kashmir, Khudwani, J&K, India; 3 Division of Biotechnology, Sher-e Kashmir University of Agricultural Sciences and Technology of Kashmir, Srinagar, J&K, India; 4 Division of Food Science and Technology, Sher-e Kashmir University of Agricultural Sciences and Technology of Kashmir, Srinagar, J&K, India; 5 Division of Agronomy, Sher-e Kashmir University of Agricultural Sciences and Technology of Kashmir, Wadura, J&K, India

**Keywords:** genes, genome, landrace, rice, sequencing, *Mushk Budji*

## Abstract

**Background:**

*Mushk Budji* is a premium, GI-tagged, heritage rice landrace of Kashmir valley, J&K, India, distinguished by its short-bold grains, strong aroma, and soft texture. While its morphological and biochemical traits have been documented, no dedicated genomic resource has previously been available. Here we report a *de novo* genome assembly of *Mushk Budji* with adequate depth.

**Methods:**

We first-time performed reference guided whole-genome sequencing of *Mushk Budji* using the Illumina NovaSeq 6000 platform, generating 64.97 Gb of raw data. The genome assembly was achieved using SPAdes and scaffolded using RagTag against the Nipponbare reference. Structural and functional annotations were conducted with AUGUSTUS and curated across NCBI, UniProt, and KEGG databases. The haplotype analysis was performed for loci related to quality using a set of contrasting genotypes.

**Results and discussion:**

The final assembly comprised 378.29 Mb with an N50 of 29.24 Mb and 97.0% BUSCO completeness. A total of 43,434 non-redundant protein-coding genes were identified, with significant enrichment in pathways related to aroma (2-acetyl-1-pyrroline) and starch biosynthesis. Allelic profiling confirmed the characteristic 8-bp deletion in *Badh2*, conferring fragrance, alongside additional genes shaping quality and adaptability. Unique haplotypes at *SSI* and *RAmy1A*, resembling those of temperate japonica variety Koshihikari, were associated with distinct soft and cohesive cooked texture of *Mushk Budji*. Comparative analyses revealed evolutionary divergence from canonical japonica references at specific loci. Selective signature analyses revealed contrasting evolutionary patterns among major quality genes. *SSI* showed evidence of balancing selection, whereas *GS3* and *Badh2* exhibited signatures of strong human-mediated selection, reflecting the historical preference for desirable grain quality and fragrance traits. Genes controlling amylose content and cooking quality (*Wx* and *Alk*) were under moderate purifying selection, indicating functional optimization.

**Conclusion:**

The genomics resource generated in the present study establishes *Mushk Budji* as a distinct temperate japonica landrace with unique allelic combinations underpinning its aroma, texture and adaptability. The dataset provides a foundation for conservation, breeding and functional genomics, ensuring the sustainable utilization of this heritage rice.

## Introduction

Heirloom rice landraces constitute a vital component of global rice biodiversity, encompassing a wide spectrum of aromatic, pigmented, nutritionally rich and regionally adapted strains that have evolved through centuries of farmer-led selection and traditional cultivation practices (Huang et al., 2012; Zhang et al., 2016). These landraces harbor unique allelic combinations and adaptive traits that are frequently absent in modern high-yielding cultivars developed through intensive breeding (Tanksley and McCouch, 1997; Xu et al., 2012; Zhao et al., 2018). Eventually, the heritage rice varieties represent an invaluable reservoir of genetic diversity for crop improvement, conservation and resilience under changing agro-climatic conditions. India serves as a primary center of genetic diversity for rice, harboring a wealth of indigenous landraces. National Gene Bank, ICAR-National Bureau of Plant Genetic Resources, New Delhi, India houses a rich repository of approximately 100,000 rice accessions (Ahlawat et al., 2022). The indigenous gene-pool comprises of numerous short-grained aromatic landraces such as *Mushk Budji*, *Kalanamak*, *Gobindobhog*, *Joha rice*, *Jeerakasala*, *Gandhakasala*, *Jeeraphool*, *Radhunipagal* and many others, each deeply embedded within specific cultural and agro-ecological contexts. Global shifts towards the traditional and specialty foods have increased a demand for heritage aromatic rice, with GI-tagged varieties commanding substantial price premiums and contributed to rural livelihoods.

Unlike most rice-growing regions of the country, the Kashmir Valley offers a unique combination of altitude, temperate climate, glacial water irrigation and fertile loamy soils, creating an exclusive ecological niche for temperate aromatic rice cultivation. This environment has facilitated the evolution and maintenance of *Mushk Budji*, a distinctive aromatic landrace uniquely adapted to the Himalayan temperate ecosystem. *Mushk Budji* is a famous Geographical Indication (GI) tagged, short-bold aromatic *japonica* rice landrace of Kashmir. It is valued for its musky aroma, soft-cooked texture and long-standing cultural significance in the Kashmir Valley (Khan et al., 2018; Roy et al., 2020). The rice kernels are characterized by short kernel length, lower length-to-breadth ratio, moderate to high cooked kernel elongation, low amylose content (13-16%), soft texture, moisture retention and enhanced palatability (Khan et al., 2018; Juliano, 1993). The hallmark fragrance of *Mushk Budji* is primarily attributed to the accumulation of 2-acetyl-1-pyrroline (2AP), the principal volatile compound responsible for aroma in rice (Shikari et al., 2026).

Despite its special status, exceptional sensory attributes and premium market value, *Mushk Budji* has remained genomically under-characterized. This gap constrains the discovery of landrace-specific alleles, structural variants and adaptive loci that underpin unique traits such as aroma, grain quality and temperate adaptation. Previous studies on *Mushk Budji* have mostly been oriented towards the PCR based validation of aroma-related genes, disease and cold tolerance (Shikari et al., 2026), gene expression (Shafi et al., 2023), glycemic response (Nowsheen et al., 2025) and the study carried out on volatiles and flavor profiling (Fayaz et al., 2024). Recent advances in next-generation and long-read sequencing technologies have enabled the generation of high-quality *de novo* genome assemblies, providing unprecedented resolution of structural variation and functional genomic elements in rice. Rice holds a distinction of the first crop plant to be successfully sequenced. The japonica variety, Nipponbare stands as a gold standard of rice genome (Project, 2005; Wang and Han, 2022). Lee et al. (2022) sequenced a famous and widely grown, japonica cultivar, Koshihikari and reported a denovo assembly of 348.7 Mb. Later, a comprehensively catalogued 3010 Rice Genomes database was developed (Mansueto et al., 2017; Wang et al., 2018), which represented the distinct genomic architectures of major sub-populations, including the indica, temperate japonica, tropical japonica, aus and other lineages. Further, the different working groups reported denovo-sequencing genome assemblies for various sub-populations (Schatz et al., 2014; Zhang et al., 2016; Du et al., 2017; Tanaka et al., 2020; Zhou et al., 2020; Qin et al., 2021). In this study, we report a *de novo* genome assembly of short-bold aromatic rice landrace, *Mushk Budji* with the aim to facilitate the characterization of loci governing aroma biosynthesis, starch metabolism and traits related to grain quality. We aim at creating a genomics resource for conservation, functional genomics studies and breeding of high-value aromatic rice varieties adapted to temperate environments.

## Materials and methods

### Plant material and genomic DNA isolation

Genetically pure seed of *Oryza sativa* L. cv. *Mushk Budji* (IRGC 34118) was obtained from the Gene Bank, Faculty of Agriculture, Sher-e-Kashmir University of Agricultural Sciences and Technology of Kashmir (SKUAST-K), Wadura Campus, J&K, India. Seedlings were raised under controlled greenhouse conditions and young fresh leaves were harvested for genomic DNA extraction. High-molecular-weight genomic DNA was isolated using the Nucleon PhytoPure Kit (GE Healthcare BioSciences) following the manufacturer’s protocol. DNA quality and integrity were assessed using agarose gel electrophoresis and the concentration was quantified using a Qubit dsDNA fluorometer (Invitrogen, Life Technologies, USA). Only samples exhibiting high purity and intact DNA were used for downstream library preparation (**Figures 1A, B**).

**Figure 1:**
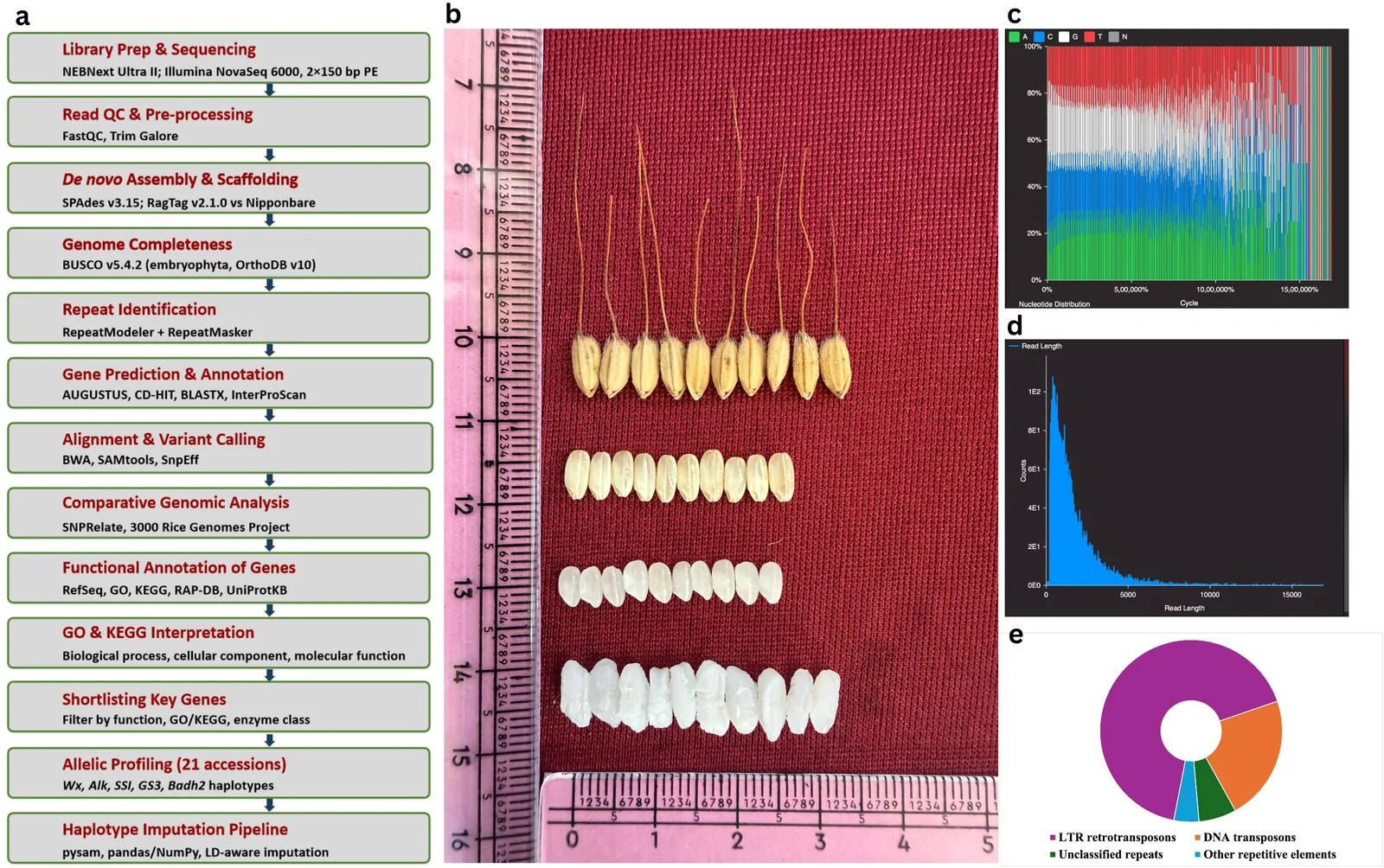
Whole-genome sequencing pipeline, grain morphology, quality control, and repeat element distribution of the aromatic rice landrace *Mushk Budji*. **(A)** Schematic representation of the complete bioinformatics workflow used for *de novo* genome assembly, annotation, comparative genomics, and allelic profiling; **(B)** Representative grain dimensions displayed alongside a centimeter ruler; Sequencing quality control metrics showing **(C)** the nucleotide base composition across sequencing cycles and **(D)** the read length distribution profile; **(E)** Relative proportion and abundance of major genomic repetitive elements, including LTR retrotransposons, DNA transposons, unclassified repeats and other repetitive sequences identified within the assembled genome.

### Evaluation of *Mushk Budji* and the reference genotypes for amylose content, aroma and grain dimensions

The non-destructive method for estimation of amylose content was employed using an NIR Grain Tester (AN-920, Kett Electric Laboratory Co. Ltd. Tokyo, Japan). Samples were placed in cuvettes with settings adjusted for polished rice and measurements were recorded. The samples of 20 polished kernels per genotype were cooked and assessed for aroma as per the sensory method described by Khanna et al. (2015). The milled grains were placed in a test tube containing 30 ml of distilled water. The tubes were stoppered and immersed in a boiling water bath for 8–10 min, then removed and allowed to cool to room temperature. Based on the sensory evaluation carried out by three independent panelists, the lines were scored for presence and absence of aroma. Further, that 10 whole, polished kernels per accession were measured for average kernel length before cooking (KLBC) and kernel width before cooking (KWBC) using a digital Vernier caliper. The genotypes were grouped as per SES, IRRI, 2013 into extra-long (>7.5 mm), long (6.61–7.5 mm), medium (5.51–6.60 mm), or short (<5.5 mm). Grain shape was assessed based on the length-breadth ratio and reported as slender (>3.0), medium (2.1–3.0), bold (1.1–2.0), or round (<1.1).

### Library preparation and Illumina sequencing

Approximately 500 ng of high-quality genomic DNA per sample was used for library preparation. Sequencing libraries were constructed using the NEBNext^®^ Ultra™ II DNA Library Preparation Kit for Illumina (Cat. No. E7770, New England Biolabs) according to the manufacturer’s protocol. Briefly, genomic DNA was fragmented to an average insert size of ~300–400 bp, followed by end-repair, A-tailing, and ligation of indexed adapters. Adapter-ligated fragments were purified and PCR-enriched to generate the final libraries. Library quantification was performed using a Qubit fluorometer and fragment size distribution was verified using the High Sensitivity DNA kit on the Agilent TapeStation system. Indexed libraries were pooled equimolarly and subjected to cluster generation on the Illumina cBot system. Sequencing was carried out on the Illumina NovaSeq 6000 platform using S4 flow cells with 2 × 150 bp paired-end chemistry. After sequencing, raw reads were demultiplexed and adapter sequences were removed using CASAVA v1.8.2 (Illumina Inc.). Library preparation and sequencing were performed by NxGenBio Life Sciences, New Delhi, India.

### Read quality control and pre-processing

Raw sequencing reads were subjected to quality assessment using FastQC v0.11.8. Quality metrics evaluated included per-base sequence quality, per-sequence quality scores, GC content distribution, and base composition. Adapter trimming and quality filtering were performed using Trim Galore v0.6.6 to remove residual Illumina universal adapters. Additional quality filtering was conducted to retain high confidence reads for assembly. Only high-quality paired-end reads were used for downstream analyses.

### *De novo* genome assembly

High-quality filtered paired-end reads were drafted into *de novo* assembly using SPAdes v3.15 (Prjibelski et al., 2020). SPAdes performs built-in read error correction followed by iterative assembly using multiple k-mer sizes (K21, K33, K55, and K77) to optimize contig construction. The resulting contigs generated from the multi-k-mer assembly were retained as the primary assembly. SPAdes incorporates an integrated read error-correction module and employs a multi-k-mer *de Bruijn* graph approach that improves contiguity and assembly accuracy for short-read datasets. Since our sequencing data consisted exclusively of Illumina short reads, SPAdes appeared to be an appropriate choice. To improve assembly contiguity and chromosomal organization, scaffolding was performed using RagTag v2.1.0 (Alonge et al., 2022) through a reference-guided approach against the *Oryza sativa* ssp. japonica Nipponbare reference genome (IRGSP-1.0) downloaded from Ensembl Plants. RagTag was used for homology-based mis-assembly correction, scaffolding, patching and scaffold merging. Assembly statistics, including total genome size, number of scaffolds, N50 and longest scaffold length, were computed using BBTools (stats.sh).

### Genome completeness assessment

Genome completeness was evaluated using BUSCO v5.4.2 (Manni et al., 2021) against the embryophyta lineage dataset from OrthoDB v10 (Kriventseva et al., 2019), using *Oryza sativa* as the reference species. BUSCO classifies orthologs into Complete (single-copy or duplicated), Fragmented, and Missing categories, providing quantitative assessment of assembly completeness.

### Repeat identification

Repetitive elements were identified using RepeatModeler (Flynn et al., 2020) to construct a *de novo* repeat library, followed by masking using RepeatMasker (Tarailo‐Graovac and Chen, 2009). Repeat classification was performed to determine the abundance of long terminal repeat (LTR) retrotransposons and other repeat families characteristic of rice genomes.

### Gene prediction and structural annotation

Gene prediction was performed using AUGUSTUS v3.1 (Stanke et al., 2006) with *Oryza sativa* as the training species. Predicted gene models were clustered using CD-HIT v4.6 (Fu et al., 2012) at a sequence similarity threshold of 90% to remove redundancy. Protein functional annotation was carried out using BLASTX and InterProScan, which integrates multiple protein signature databases for comprehensive identification of conserved domains, protein families, functional sites, and associated annotations. Protein sequences were queried against Pfam (v31.0), PRINTS (v42.0), ProDom (v2006.1), PROSITE Patterns (v20.132), PROSITE Profiles (v20.132), SMART (v7.1), SUPERFAMILY (v1.75), and TIGRFAMs (v15.0). The combined results were used to assign protein domains, predict molecular functions, classify proteins into families and retrieve associated Gene Ontology (GO) terms. Additional annotation was performed using UniProt and the KEGG Automatic Annotation Server (KAAS) (Moriya et al., 2007). Gene Ontology (GO) terms were assigned and GO classification plots were generated using WEGO (Ye et al., 2018) and tRNA and rRNA genes were identified using tRNAscan-SE 2.0 (Chan et al., 2021) and Barrnap, respectively.

### Reference-based alignment and variant analysis

Adapter-trimmed reads were aligned to the *Oryza sativa* Nipponbare (IRGSP-1.0) reference genome using BWA v0.7.17 (Li and Durbin, 2009). SAMtools v0.1.19 (Li et al., 2009) was used for conversion, sorting, and indexing of alignment files. Variants were called using SAMtools mpileup and filtered based on base quality, mapping quality, and read depth thresholds. SNPs and InDels were annotated using SnpEff (Cingolani et al., 2012) with the rice gene model database and classified into genic and intergenic categories, including synonymous and non-synonymous substitutions.

### Comparative genomic analysis

Comparative analysis was performed against publicly available rice genome datasets, including variants from the 3000 Rice Genomes Project. Variants were merged and analyzed using SNPRelate based on identity-by-state (IBS) distance to infer genetic relationships.

### Functional annotation of predicted genes

Functional annotation of predicted genes was performed using multiple public databases such as, NCBI RefSeq, Gene Ontology (GO) and Kyoto Encyclopedia of Genes and Genomes (KEGG) pathway. Each predicted gene was assigned a gene model identifier and protein homology searches were used to identify the most similar protein sequences from reference databases. The annotation process provided the information on protein function, gene symbols, enzyme classification, GO terms and metabolic pathway associations.

The compounds associated with key rice quality traits, including aroma and starch metabolism were identified through an extensive review of published literature. Major aroma-related compounds such as 2-acetyl-1-pyrroline (2-AP), along with other volatile metabolites derived from lipid and amino acid pathways were considered, while compounds involved in starch biosynthesis and modification were compiled based on established biochemical studies. Subsequently, the candidate genes associated with these compounds and pathways were identified using publicly available genomic resources and previous reports. Genes were shortlisted based on their involvement in relevant biological processes such as fatty acid metabolism, amino acid biosynthesis, carbohydrate metabolism, and lipid oxidation. Functional annotation of the identified genes was performed using the Rice Annotation Project Database (RAP-DB) (Sakai et al., 2013) and the UniProt Knowledgebase (UniProtKB). For each gene, detailed information including gene identifiers, protein names, enzyme classification (EC numbers) and functional descriptions was retrieved. Further, the classification of genes was carried out using GO annotations: biological process, molecular function and cellular component categories, along with pathway mapping through the KEGG database to determine their involvement in metabolic pathways such as starch and sucrose metabolism, fatty acid biosynthesis and amino acid metabolism.

Based on available functional evidence, the genes were categorized into directly and indirectly associated groups. Directly associated genes included those with a well-established role in aroma or starch biosynthesis, whereas indirectly associated genes were those involved in precursor formation, intermediate metabolic steps, or regulatory functions contributing to these traits. All annotated genes were compiled into a structured dataset comprising gene ID, RefSeq ID, protein name, GO terms and KEGG pathways, which was further used for downstream functional analysis and interpretation of gene-trait associations.

### Gene ontology functional interpretation

Gene Ontology analysis classified annotated genes into three major categories: biological process, molecular function, and cellular component. Biological process terms describe the physiological processes in which genes participate, such as metabolic processes, stress responses, signal transduction, and transcriptional regulation. Molecular function annotations indicate the biochemical activities of proteins, including ATP binding, protein kinase activity, oxidoreductase activity, and DNA binding. Cellular component annotations identify the subcellular localization of proteins, such as the nucleus, membrane, chloroplast, cytoplasm, and endoplasmic reticulum, providing insights into the cellular roles of these genes.

### KEGG pathway interpretation

KEGG pathway analysis revealed that several genes were associated with metabolic and signaling pathways, including terpenoid biosynthesis, steroid biosynthesis, carbon metabolism, and sphingolipid metabolism. These pathways are involved in essential biological functions such as secondary metabolite production, lipid metabolism, and energy metabolism. The identification of genes linked to these pathways highlights the complex metabolic network present in the *Mushk Budji* genome.

### Short listing important genes

To identify biologically significant genes, functional annotation parameters were used to filter genes based on protein function, Gene Ontology annotation, KEGG pathway involvement and enzyme classification. Genes with clear functional annotations, such as transcription factors, enzymes, transporters, and signaling proteins, were prioritized. In contrast, genes annotated as hypothetical or uncharacterized proteins without GO or pathway information were excluded. This filtering strategy enabled the identification of genes associated with metabolic pathways, stress responses, and regulatory processes. Several genes identified in the annotation dataset represented important biological functions. Enzymes such as sphingosine-1-phosphate lyase participate in lipid metabolism and signaling pathways, while enzymes like DXP reductoisomerase are involved in terpenoid biosynthesis pathways associated with secondary metabolite production. Transport proteins such as phosphate transporters play an important role in nutrient uptake and plant growth.

### Allelic profiling of quality-associated genes

A comparative allelic profiling was performed using a panel of 21 rice germplasm lines representing three major grain quality categories: short-grained aromatic, long-grained aromatic (Basmati-type), and short-grained non-aromatic rice cultivars. Whole-genome resequencing data was generated for indigenous landraces from the Kashmir and Jammu Himalayan regions (*Bata Baber*, *Begum*, *Ram Hall*, *Mushkandi I*, *GS-28* and *Bansali*), locally bred cultivars (*K-332* and *Shalimar Sugandh-1*), premium-quality rice varieties (*Pusa Basmati 1121* and *Koshihikari*), and diverse aromatic rice collections representing both short- and long-grained types (*Sonasal*, *Taraori Basmati*, *Gobindobhog*, *Katarni Rice*, and *Type-3 Basmati*). In addition, sequence alignment files (BAM format) for *IR64*, *Sant Andrea*, *Karnal Local*, and *Sadri Rice 1* were retrieved from the 3K Rice Genome Project repository hosted on the AWS public server using the AWS CLI (--no-sign-request mode). These cultivars were specifically included because they represent historically and commercially important rice quality archetypes preferred across diverse consumer traditions worldwide, ranging from high-yielding *indica* cultivar (*IR-64*), premium Mediterranean *japonica* rice (*Sant Andrea*), heritage aromatic Basmati (*Karnal Local*), to the highly aromatic Persian-type Sadri rice group (*Sadri Rice 1*), thereby providing a broader comparative framework for positioning the quality-associated allelic architecture of *Mushk Budji*. The complete set of 21 accessions was subsequently used for haplotype analysis of major quality-associated genes, such as *Wx*, *Alk*, *SSI*, *GS3*, *RAmy1A*, and *Badh2*.

### Computational framework for allelic profiling

To characterize genomic variation across targeted loci, we developed a custom Python pipeline using the pysam library to process positional pileup data from high-quality reads (MAPQ ≥ 30) aligned to the *Oryza sativa* ssp. *japonica* cv. Nipponbare (IRGSP-1.0) reference. A stringent genotyping framework (Q ≥ 40) was implemented to mitigate artifacts from soft-clipped read tails and strand bias, assigning genotypes based on allele frequency thresholds of ≥ 0.75 for homozygous and ≥ 0.25 for heterozygous calls. Following a cohort-aware filter to remove low-depth singletons (< 3×), high-confidence variants were functionally annotated using RAP-DB transcript coordinates (Sakai et al., 2013) to identify synonymous, missense, frameshift, and splice-site alterations, with Grantham distances employed to quantify the physicochemical impact of amino acid substitutions.

To resolve distinct functional haplotypes, we implemented a custom LD-aware imputation pipeline in Python, leveraging the pandas and NumPy libraries. We first established “core” haplotypes from accessions with high-confidence, homozygous calls, where even a single allelic mismatch (mismatch distance > 0) at targeted functional loci was sufficient to define a unique haplotype. For accessions with missing or heterozygous data, unresolved alleles were computationally assigned using a Nearest-Neighbor approach that calculated the mismatch rate across the entire intragenic background. Ambiguous alleles were then imputed based on the profile of the closest genetic match; in instances of tied genetic distances, a 5-kb flanking Linkage Disequilibrium (LD) window was utilized to break ties, ensuring biologically consistent haplotype assignments based on localized genetic linkage.

### Evolutionary population genetics analysis

To quantify selection pressure and genetic diversity, we integrated a comprehensive evolutionary analytics module into our pipeline, applying standard population genetic estimators to all identified high-confidence variants.

Nucleotide Diversity and Neutrality Testing: We calculated per-site nucleotide diversity (π) using the unbiased estimator of expected heterozygosity as described by Nei (1987). To test the neutral mutation hypothesis and identify potential genomic signatures of selective sweeps or population expansion, Tajima’s D was computed based on the frequency of segregating biallelic SNPs as outlined by Tajima (1989).

Selection Pressure Estimates (*K_a_*/*K_s_*): For coding regions, we employed the method developed by Nei and Gojobori (1986) to estimate the ratio of non-synonymous (*d_N_*) to synonymous (*d_S_*) substitution rates. This *K_a_*/*K_s_* ratio served as a proxy for identifying purifying, neutral, or positive selection acting upon the protein-coding sequence of the targeted loci.

Substitution Bias and Quality Control: The transition-to-transversion (Ti/Tv) ratio was determined to assess variant calling quality and mutational bias. Furthermore, we analyzed the distribution of SNPs across codon positions to verify whether synonymous changes predominated at the third (wobble) position, a classic indicator of evolutionary constraint.

## Results

### Sequencing output and data quality

Whole-genome sequencing of *Oryza sativa* cv. *Mushk Budji* generated 64.970 Gb of raw data, comprising 216,568,247 reads. Sequencing was performed using Illumina NovaSeq chemistry, and base calling was conducted using CASAVA software. The per-base nucleotide composition plot generated from Illumina whole-genome sequencing demonstrated the distribution of A, T, G, C, and ambiguous (N) bases across sequencing cycles, serving as an important quality control metric. In this dataset, reads were processed using a quality trimming threshold of 5% and a minimum read length cutoff of 30 bp, ensuring that low-confidence bases, particularly toward the read termini, were effectively removed while retaining sufficient sequence length for reliable downstream analyses. The relatively stable nucleotide proportions observed across most sequencing cycles indicate that these thresholds successfully preserved high-quality sequence information without introducing compositional bias. Quality assessment indicated no low-quality sequences in the raw dataset demonstrating high sequencing fidelity. Based on the final assembled genome size, the sequencing depth corresponded to approximately 171× genome coverage ensuring sufficient coverage for accurate *de novo* assembly and downstream analyses (**Figures 1C, D**).

### *De novo* genome assembly of *Mushk Budji* and comparative assessment

*De novo* genome sequencing and assembly of the aromatic rice landrace *Mushk Budji* generated a high-quality draft genome with a total assembled length of 378.29 Mb, represented by 4,479 sequences. This assembly size is comparable to reported genome sizes of *Oryza sativa* subsp *japonica* and indica cultivars (≈370–390 Mb), indicating near-complete genome recovery. The assembly exhibited strong contiguity, with an N50 of 29.24 Mb and a longest sequence of 44.14 Mb, values that are comparable to or exceed those reported for several landrace-based *de novo* rice assemblies and approach chromosome-scale continuity achieved in reference-guided assemblies (**Table 1**).

**Table 1:** Genome assembly and annotation statistics of *Mushk Budji*.

Feature	Statistic
*Genome assembly*	
Total assembled genome size (bp)	378,288,223
Number of sequences	4,479
Mean sequence length (bp)	84,458.19
Longest sequence (bp)	44,143,916
Shortest sequence (bp)	200
N50 (bp)	29,236,913
*Gene annotation*	
Total predicted genes	45,807
Transcripts	45,807
Coding sequences (CDS)	201,003
Exons	211,732
Genes after clustering	43,434
*Coding sequence statistics*	
Total CDS length (bp)	60,899,191
Mean gene length (bp)	1,402.11
Longest gene (bp)	16,905
Shortest gene (bp)	70
CDS N50 (bp)	1,896

### Gene prediction and structural annotation in comparison with cultivated rice genomes

Structural annotation of the *Mushk Budji* genome predicted 45,807 protein-coding genes. Of these 43,434 non-redundant protein coding genes were retained, the count that falls within the expected range for *O. sativa* genomes. This elevated initial gene prediction likely reflects landrace-specific gene duplications, allelic diversity and expanded gene families associated with environmental adaptation and stress responsiveness. The total length of predicted coding sequences was 60.90 Mb, with a mean gene length of 1,402 bp and a CDS N50 of 1,896 bp. A total of 201,003 CDS and 211,732 exons were identified, indicating complex gene architectures consistent with previously characterized rice genomes (**Table 2**).

**Table 2:** Comparative genome assembly and annotation statistics of *Mushk Budji* and reference rice genomes.

Feature	*Mushk Budji*	Nipponbare (*Japonica*)	IR64 (*Indica*)	Shuhui (*Indica*)
Assembly strategy	Reference-based	*De novo*	*De novo*/hybrid	*De novo*/hybrid
Genome size (Mb)	378.29	~373	~389	~395
Assembly level	Scaffold-level	Chromosome-level	Chromosome/near-chromosome	Chromosome-level
Number of sequences	4,479	12	12	12
N50 (Mb)	29.24	~30-32	~25-30	~30-35
Longest sequence (Mb)	44.14	~43	~41	~45
Predicted genes	43,434	~39,000-41,000	~41,000-42,000	~42,000-44,000
Mean gene length (bp)	1,402	~1,400	~1,380	~1,390
BUSCO completeness (%)	>97	>95	>95	>95
Repeat content (%)	Comparable	~35-40	~38-42	~40-45

### Genome completeness assessment and repeat element annotation

Genome completeness of the *Mushk Budji* assembly was evaluated using Benchmarking Universal Single-Copy Orthologs (BUSCO) with the embryophyta_odb10 lineage dataset (OrthoDB v10; n = 425 ortholog groups). The assembly demonstrated a high level of completeness, with 97.0% complete BUSCOs, comprising 93.9% single-copy and 3.1% duplicated orthologs. Only 1.6% of BUSCOs were fragmented and 1.4% were missing. In total, 412 of the 425 conserved ortholog groups were recovered as complete, including 399 single-copy and 13 duplicated genes (**Table 3**; **Supplementary Figure 1**). Repeat annotation revealed that repetitive sequences constitute approximately 45% of the *Mushk Budji* genome, consistent with previously reported repeat proportions in cultivated *Oryza sativa* genomes. Long terminal repeat (LTR) retrotransposons represented the predominant class, accounting for an estimated 30% of the genome, highlighting their major contribution to genome size and structural organization. DNA transposons comprised approximately 10%, while unclassified repeats (3%) and other repetitive elements (2%) collectively contributed a smaller fraction of the genome (**Figure 1E**).

**Table 3:** BUSCO assessment statistics for the *Mushk Budji* genome assembly.

Category	Count	Percentage (%)
Total BUSCO groups searched	425	100
Complete BUSCOs (C)	412	97.0
Complete and single-copy (S)	399	93.9
Complete and duplicated (D)	13	3.1
Fragmented BUSCOs (F)	7	1.6
Missing BUSCOs (M)	6	1.4

Collectively, BUSCO completeness metrics and repeat landscape characteristics confirm that the *Mushk Budji* genome assembly is both structurally robust and biologically representative of cultivated rice genomes, while retaining unique features expected from an aromatic landrace adapted to temperate agro-ecological conditions.

### Genome-wide variation

Genome-wide variant calling of the *Mushk Budji* genome against the *Nipponbare* reference (IRGSP 1.0) revealed substantial genetic divergence, summing up to 705,359 single nucleotide polymorphisms (SNPs) and 84,861 insertions and deletions (InDels) across the 12 chromosomes. SNP counts ranged from 28,303 (Chr3) to 100,970 (Chr4). The overall genome-wide SNP density was 1,890 SNPs/Mb, with per-chromosome densities fluctuating between 777 SNPs/Mb (Chr3) and 4,025 SNPs/Mb (Chr10). The transition-to-transversion (Ti/Tv) ratio was 2.37 genome-wide ranging from 2.125 (Chr4) to 2.611 (Chr5) indicating a characteristic transition predominance (**Table 4**; **Supplementary Table 1**). Furthermore, the overall InDel density was 227 InDels/Mb, with chromosomal counts varying from 4,674 (Chr9) to 10,352 (Chr4) (**Table 5**; **Supplementary Table 2**). Insertions (41,550) and deletions (43,311) were relatively balanced, with mean lengths of 3.2 bp and 4.0 bp, respectively. Technical reliability was underscored by high sequencing depths, averaging 117.3× for SNPs and 97.5× for InDel positions (**Figure 2**; **Supplementary Figure 3**).

**Table 4:** SNP phase, depth and distribution in *Mushk Budji* genome.

Chromosome	1	2	3	4	5	6	7	8	9	10	11	12	Genome total
Total SNPs	45081	38411	28303	100970	40577	70028	72172	42985	38824	93405	67593	67010	705359
Transitions (Ti)	31875	27187	20134	68657	29341	49590	50656	30753	27661	66681	46397	46830	495762
Transversions (Tv)	13206	11224	8169	32313	11236	20438	21516	12232	11163	26724	21196	20180	209597
Ti/Tv	2.414	2.422	2.465	2.125	2.611	2.426	2.354	2.514	2.478	2.495	2.189	2.321	2.365
Avg depth (×)	118.6	121.5	129.8	114.6	123.6	115.5	109.5	122.3	120.8	117.1	114.2	117	117.3
Variant density (per Mb)	1041.83	1068.84	777.26	2844.01	1354.44	2240.98	2430.23	1511.27	1687.07	4024.81	2329.1	2433.91	1889.8
A>C	1768	1443	988	4196	1435	2581	2754	1536	1403	3396	2642	2533	26675
A>G	7773	6579	4663	16425	7028	11645	12323	7247	6595	16088	11196	11331	118893
A>T	1888	1631	1167	4509	1550	2900	3012	1711	1534	3677	2973	2840	29392
C>A	1748	1497	1102	4136	1470	2768	2911	1569	1489	3587	2756	2561	27594
C>G	1229	1026	827	3400	1080	2002	2124	1212	1143	2679	2159	2090	20971
C>T	8129	7053	5368	18030	7561	13116	12910	7961	7347	17120	11865	12181	128641
G>A	8091	7128	5372	17825	7762	13207	12974	8115	7180	17230	11927	12164	128975
G>C	1176	1093	720	3218	1123	1921	2070	1217	1145	2761	2248	2113	20805
G>T	1749	1509	1133	4439	1565	2694	2853	1694	1468	3511	2755	2618	27988
T>A	1908	1624	1169	4522	1565	2897	3001	1732	1587	3708	2992	2913	29618
T>C	7882	6427	4731	16377	6990	11622	12449	7430	6539	16243	11409	11154	119253
T>G	1740	1401	1063	3893	1448	2675	2791	1561	1394	3405	2671	2512	26554

**Table 5:** InDel polymorphism and distribution in *Mushk Budji* genome.

Chromosome	Total InDels	Insertions	Avg ins length (bp)	Deletions	Avg del length (bp)	Avg depth (×)	Variant density (per Mb)
1	7832	3870	3.0	3962	3.6	97.2	181.0
2	6351	3033	3.0	3318	3.7	96.5	176.7
3	5279	2613	2.8	2666	3.7	100.6	145.0
4	10352	5088	3.1	5264	4.0	97.7	291.6
5	5569	2729	3.0	2840	3.8	101.1	185.9
6	7840	3855	3.3	3985	4.0	96.5	250.9
7	8486	4184	3.5	4302	4.3	96.1	285.8
8	5385	2620	3.2	2765	4.0	97.1	189.3
9	4674	2211	3.0	2463	4.1	98.9	203.1
10	8971	4418	3.5	4553	4.6	98.4	386.6
11	7373	3635	3.4	3738	4.1	94.1	254.1
12	6749	3294	3.3	3455	4.0	97.7	245.1
Genome total	84861	41550	3.2	43311	4.0	97.5	227.4

**Figure 2:**
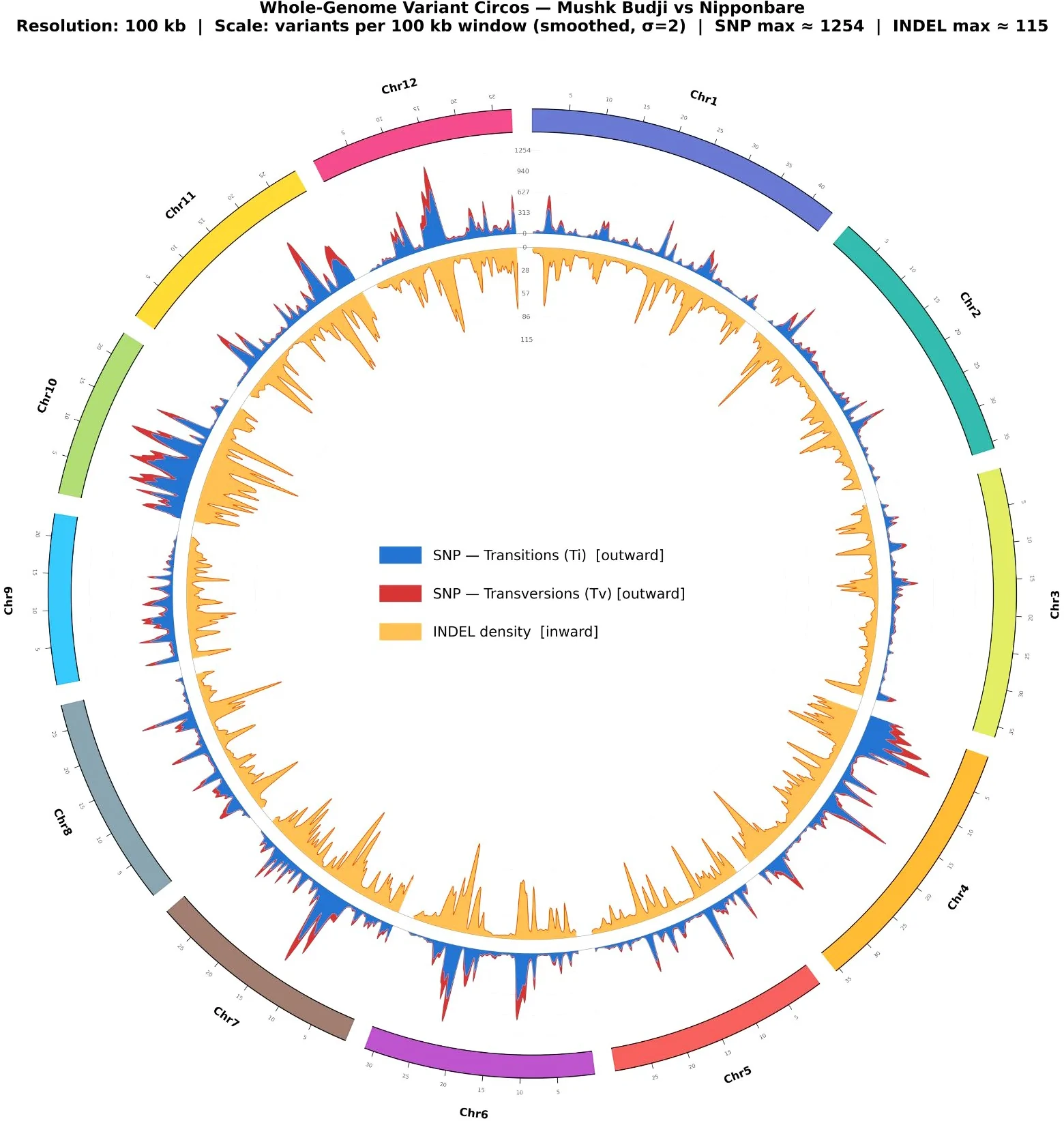
Chromosome-scale Circos map of genomic variation highlighting SNP and InDel landscapes between *Mushk Budji* and Nipponbare genomes. Variant frequencies were calculated using non-overlapping 100 kb genomic windows and smoothed using a Gaussian filter (σ = 2). Peaks indicate genomic regions with elevated sequence divergence, highlighting chromosomal hotspots of genetic variation across the Mushk Budji genome relative to Nipponbare.

### Correlation between SNP and InDel densities

To analyze the correlation between the SNPs and InDels, each chromosome was partitioned into consecutive, non-overlapping 100 kb windows. The relationship between SNP and InDel frequencies was assessed using Pearson correlation coefficients calculated for each chromosome. All 12 chromosomes exhibited strong, positive correlations, with *r* values ranging from 0.739 (Chr4) to 0.879 (Chr1). These correlations were highly significant with the strongest associations observed on Chr1 (*r* = 0.879), Chr5 (*r* = 0.872), and Chr7 (*r* = 0.874). The strong correlation prevailed up to the SNP: InDel count of 750:75 per 100kb. The consistency of these high correlation coefficients across the genome demonstrates a robust co-occurrence of SNPs and InDels (**Figure 3**; **Supplementary Table 3**).

**Figure 3:**
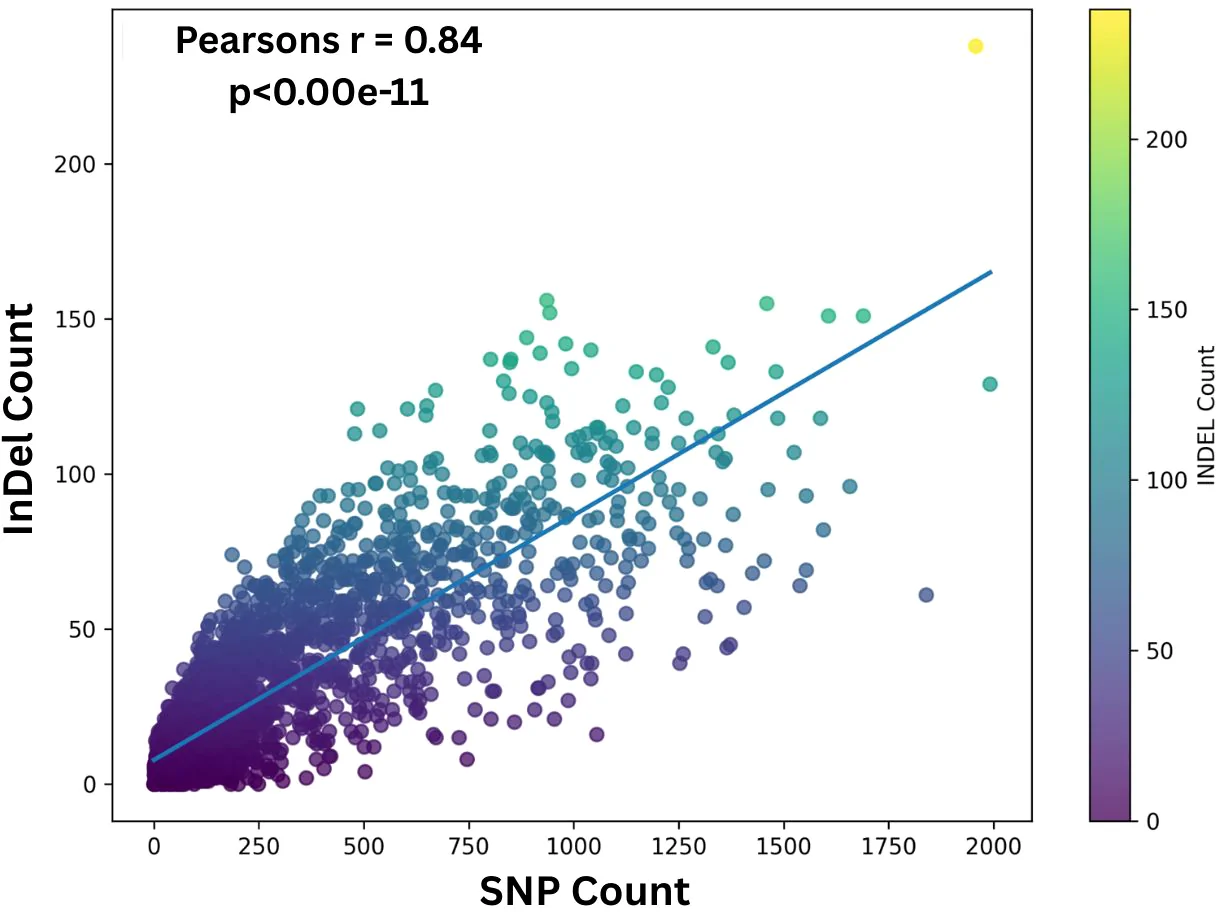
Association between SNP abundance and INDEL frequency across genomic regions of the *Mushk Budji* rice genome.

### Functional annotation of *Mushk Budji* genome

Functional annotation was performed for the 43,434 non-redundant predicted genes in the *Mushk Budji* genome using multiple public databases, including Gene Ontology (GO) and KEGG. Of the total predicted genes 35,864 (82.57%) were successfully assigned GO: cellular components, 11201 (25.78%) with GO: molecular function, 8698 (20.02%) to GO: biological processes. KEGG pathway annotation assigned 4,691 genes (10.8%) to known metabolic and signaling pathways.

Gene ontology (GO) classification: Total of 14,363 genes were annotated as rice genes of which 14,100 corresponded to *Oryza sativa* subsp. *japonica* and 263 were annotated as *Oryza sativa*. A subset of genes (11,202) possessing complete functional annotation, including Gene Ontology classification (biological process, cellular component, and molecular function), enzyme commission (EC) numbers, and KEGG pathway assignments, were identified from the *Mushk Budji* genome dataset. Within the biological process category, 8699 genes were associated with metabolic processes, cellular processes and biological regulation. The cellular component category was dominated by 7569 genes localized to membranes, organelles and intracellular structures, while the molecular function category was enriched for binding and catalytic activity terms.

KEGG pathway annotation: KEGG pathway analysis assigned 4,692 genes to diverse metabolic and signaling pathways. Genes involved in carbohydrate metabolism, energy metabolism, amino acid biosynthesis, and signal transduction pathways were well represented. The presence of genes across major metabolic pathways suggested a complete and functional metabolic repertoire typical of cultivated rice, while also providing scope for identifying unique pathway variants associated with aroma, stress tolerance and local adaptation. Furthermore, 3,221 genes were assigned EC numbers, suggesting that these genes encode enzymes responsible for catalyzing metabolic reactions. From the annotated dataset, 134 genes were shortlisted as functionally important based on EC numbers, pathway association and functional relevance. These genes were primarily associated with metabolic pathways, secondary metabolite biosynthesis, grain quality traits and stress response mechanisms (**Figure 4**; **Supplementary Table 4**).

**Figure 4:**
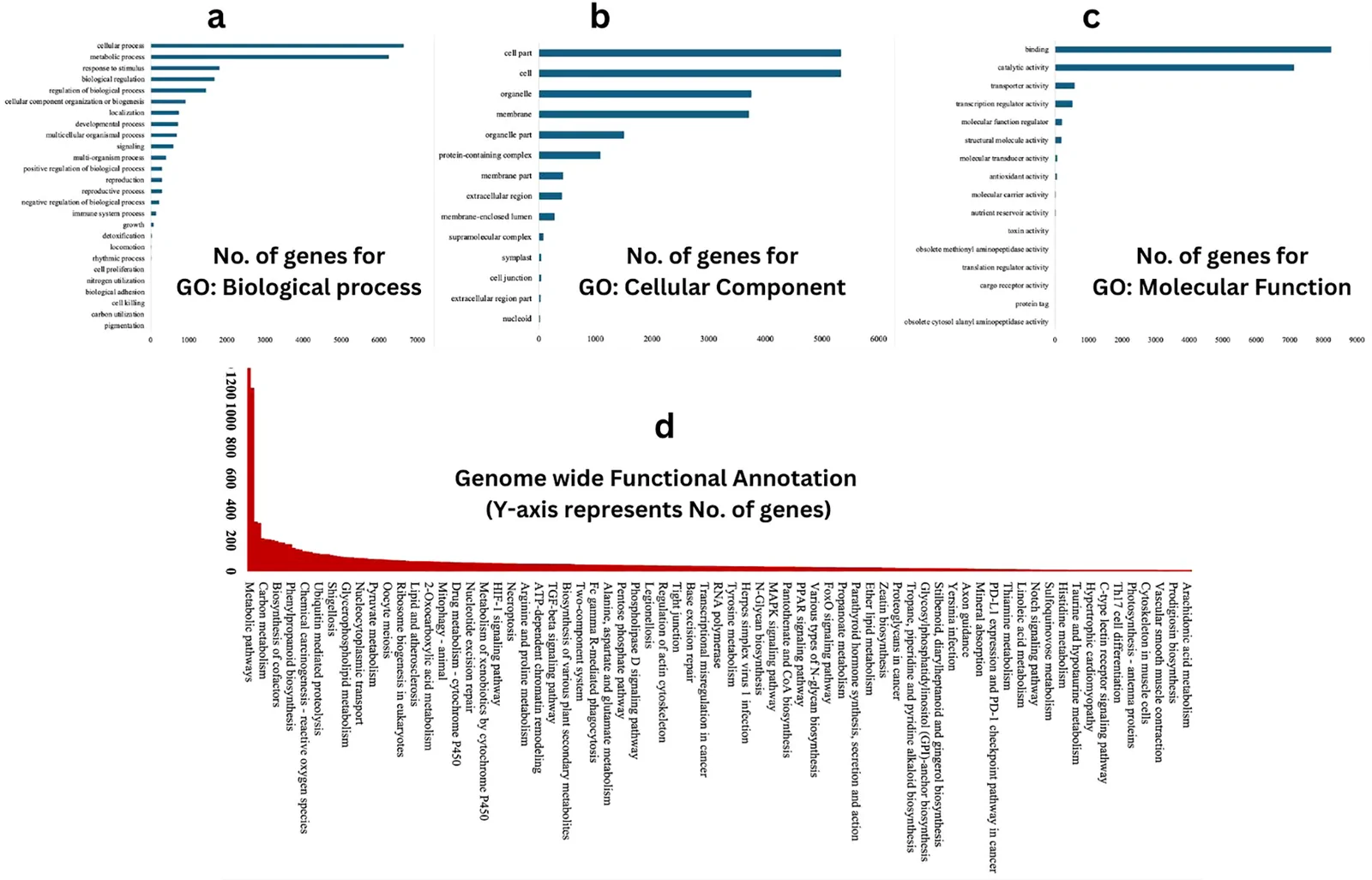
Genome-wide functional characterization of annotated genes based on Gene Ontology and pathway enrichment analyses. **(A–C)** Gene Ontology (GO) classification displaying the distribution and number of genes enriched across the three main categories: **(A)** Biological Process; **(B)** Cellular Component, and **(C)** Molecular Function; **(D)** Genome-wide pathway annotation mapping the total number of genes associated with specific metabolic and functional pathways.

### Gene distribution for major quality and adaptability traits

The specific filtering of the data was carried out for few of the quality related traits as given below:

Starch: A total of 135 candidate genes associated with starch metabolism were identified from the annotated dataset based on functional annotation, EC numbers, Gene Ontology terms and KEGG pathway information. Among these, 29 genes were directly involved in starch biosynthesis and degradation pathways, including enzymes responsible for key reactions in starch metabolism such as starch synthesis, carbohydrate conversion and polysaccharide modification. These genes encode enzymes that directly regulate the formation and structural organization of starch components such as amylose and amylopectin, which are critical determinants of grain quality and cooking properties in rice. In addition, 106 genes were indirectly associated with starch metabolism, primarily involved in broader carbohydrate metabolic processes, sugar interconversion and energy metabolism that provide substrates or regulatory support for starch biosynthesis and accumulation during grain development. The identification of both direct and indirect starch-related genes highlights the complex metabolic network controlling starch synthesis and grain quality traits in rice.

Aroma: A total of 232 genes associated with aroma-related metabolic pathways were identified in the annotated genome dataset. Among these, 16 genes encoding aldehyde dehydrogenase family proteins were directly related to aroma biosynthesis. These genes play a crucial role in the metabolic pathway responsible in producing 2-acetyl-1-pyrroline (2AP), the major compound contributing to the characteristic fragrance of aromatic rice. Functional annotation based on GO terms and KEGG pathway analysis indicated that these genes participate in aldehyde metabolic processes and oxidation-reduction reactions, which are closely associated with the biochemical pathway leading to aroma formation in rice grains. The remaining 216 genes were indirectly associated with aroma formation through their roles in amino acid metabolism, secondary metabolite biosynthesis, lipid metabolism, and oxidation-reduction processes. These genes are mainly associated with amino acid metabolism, lipid metabolism, secondary metabolite biosynthesis, carbohydrate metabolism and oxidation-reduction processes. Such pathways contribute to the formation of intermediate metabolites and biochemical precursors that can affect the synthesis and accumulation of volatile aroma compounds in rice. Therefore, although these genes do not directly catalyze the formation of aroma molecules, their involvement in broader metabolic and regulatory processes suggests an indirect role in determining the aromatic characteristics and grain quality of rice.

Secondary metabolites: A total of 43 genes associated with secondary metabolite biosynthesis were identified in the annotated dataset and govern flavonoid biosynthesis, phenylpropanoid metabolism, carotenoid biosynthesis and terpenoid biosynthesis. These pathways produce a diverse group of secondary metabolites that play crucial roles in plant defense, pigmentation, antioxidant activity and stress tolerance. Several enzymes encoded by these genes, such as chalcone synthase, flavonoid monooxygenase, flavanone dioxygenase, cytochrome P450 proteins, terpene synthases and 4-coumarate-CoA ligases, were identified as key components of these pathways.

Abiotic stress responsive genes: A total of 28 genes associated with abiotic stress response were identified based on Gene Ontology (GO) annotations. These genes are involved in important biological processes such as response to heat (GO:0009408), response to cold (GO:0009409), response to salt stress (GO:0009651/GO:1902074), and response to water deprivation (GO:0009414). The identified genes encode several classes of stress-related proteins including small heat shock proteins (sHSPs), heat shock protein 70 (HSP70), annexins, receptor-like cytoplasmic kinases, trehalose-6-phosphate phosphatases, ABC transporters, calcium-transporting ATPases, BTB domain-containing proteins, and thioredoxin domain proteins. Heat shock proteins function as molecular chaperones that stabilize proteins and prevent aggregation during heat stress, while annexins participate in calcium-mediated signaling and membrane stability under stress conditions. Receptor-like kinases and BTB domain proteins are involved in signal transduction and regulation of stress-responsive pathways, whereas ABC transporters and calcium-transporting ATPases regulate ion transport and cellular homeostasis during salt and drought stress. Additionally, thioredoxin proteins contribute to redox balance and protection against oxidative damage. Collectively, these genes play essential roles in maintaining cellular stability, metabolic balance, and adaptive responses under adverse environmental conditions, thereby contributing to stress tolerance and overall plant resilience in rice.

Defense-related genes: A total of 145 genes associated with biotic stress response were identified based on functional annotation and GO analysis. These genes pertained to biological processes related to pathogen (bacteria, fungi and other microorganisms) defense response, as well as innate immune signaling mechanisms, systemic acquired resistance and plant-type hypersensitive response. The annotated genes belonged to various classes of defense-related proteins such as NBS-LRR disease resistance proteins, receptor-like kinases, calcium-dependent protein kinases, MAP kinase kinases, lectin receptor kinases, ubiquitin transferases, thaumatin-like proteins and lipid transfer proteins. These proteins play important roles in pathogen recognition, signal transduction, activation of immune responses and regulation of defense-related pathways **Supplementary Figure 2**).

### Allele mining at genes related to rice quality

The distinct functional haplotypes at quality related loci were delineated through a custom LD-aware imputation pipeline run in NumPy libraries (**Supplementary Tables 5**, **6**). The genetic profile, with respect to the primary quality related genes (*Wx*, *Alk*, *SSI, RAmy1A, GS3* and *Badh2*) in *Mushk Budji*, was compared against the cultivars representing distinct categories: short-grained aromatic, long-grained aromatic (Basmati) and short-grained non-aromatic. *Wx* locus serves as the primary determinant of amylose content (AC) in rice, directly influencing its sensory attributes. While several functional SNPs contribute to AC variation, the G/T SNP at the Intron 1 splicing site accounts for the significant amount of phenotype variation. *Mushk Budji* carried G at Intron 1 and belonged to Hap_3 (**Figure 5A**; **Supplementary Table 7**). This haplotype corresponds to the well characterized *Wx^in^* allele that is also present in premium varieties or landraces, including, Koshihikari, IR-64, Pusa Basmati 1121, Karnal Local and Gobindobhog. In comparison, the *Wx^b^* allele was observed in K-332, Ram Hall and Sant Andrea, whereas the *Wx^lv^* allele was represented by Bansali, Katarni Rice and Begum, besides, *Wx^a^* allele carried by Shalimar Sugandh 1. *Alk* serves as the major controller of Gelatinization Temperature (GT) and cooking time in rice. *Mushk Budji* was found to be the carrier of Hap_1, a version of the *Alk^c^* allele characterized by SNPs at Exon 8 (733, 864, and 865 bp) (**Figure 5B**). This haplotype is also shared by local landraces, Mushkandi I and Ram Hall and other cultivars like, Shalimar Sugandh 1 and Katarni Rice. Although Basmati types also possess the *Alk^c^* allele, they are categorized under Hap_3 due to differentiating variants at Exon 1 (C→G), Intron 4 (G→A), and Exon 8 (A→G). Therefore, *Mushk Budji* shares a distinct *Alk* signature that differentiates it from the long-grained Basmati (*Alk^c^*). In contrast, K-332, Koshihikari and Sant Andrea carried Hap_2, corresponding to the *Alk^b^* allele. *SSI* encodes Starch Synthase I, a core enzyme in the starch biosynthetic pathway of the rice endosperm, responsible for the elongation of short-chain amylopectin. The functional haplotype, Hap_1, was found in *Mushk Budji*, a genetic signature shared with varieties such as IR-64, Koshihikari and Sant Andrea. This haplotype was also prevalent among local cultivars, Begum, K-332, and Mushkandi I, while most of the Basmati-type cultivars, such as Pusa Basmati 1121 and Karnal Local, and short-grained aromatic cultivars, including Gobindobhog and Sonasal, carried Hap_2 as the predominant haplotype. *GS3* is the primary determinant of grain size and weight in rice. *Mushk Budji* carried Hap_1, characterized by the C allele at the Exon 2 stop-codon (**Figure 5C**; **Supplementary Table 8**). This functional allele maintains the short-grain phenotype, distinguishing the landrace from long-grain varieties (such as IR64, Karnal Local and Pusa Basmati 1121) that possess A allele (causing truncated protein) associated with Hap_2. Koshihikari and local landraces such as Ram Hall and K-332, carried *Mushk Budji* allele. *RAmy1A* gene, which encodes endosperm α-amylase, determines the Rapid Visco Analyzer (RVA) profile. Pasting behavior is significantly modulated by a functional T/G single nucleotide polymorphism (SNP) located at -1947 bp within the *RAmy1A* promoter. *Mushk Budji* harbored desirable ‘T’ allele, that confers it a specific taste and textural properties. *Badh2* locus serves as the primary genetic determinant of aroma in rice, directly regulating the concentration of 2-acetyl-1-pyrroline (2AP), the compound responsible for the characteristic fragrance. Of the six major haplotypes, *Mushk Budji* carried Hap_4. This specific haplotype comprises of well-known 8-bp deletion in Exon 7, a loss-of-function mutation that confers fragrance in most of the aromatic varieties. This haplotype is also present in Pusa Basmati 1121, Karnal Local, Sadri Rice 1, Ranbir Basmati, Taraori Basmati and Gobindobhog, etc. (**Figure 5D**; **Supplementary Table 9**).

**Figure 5:**
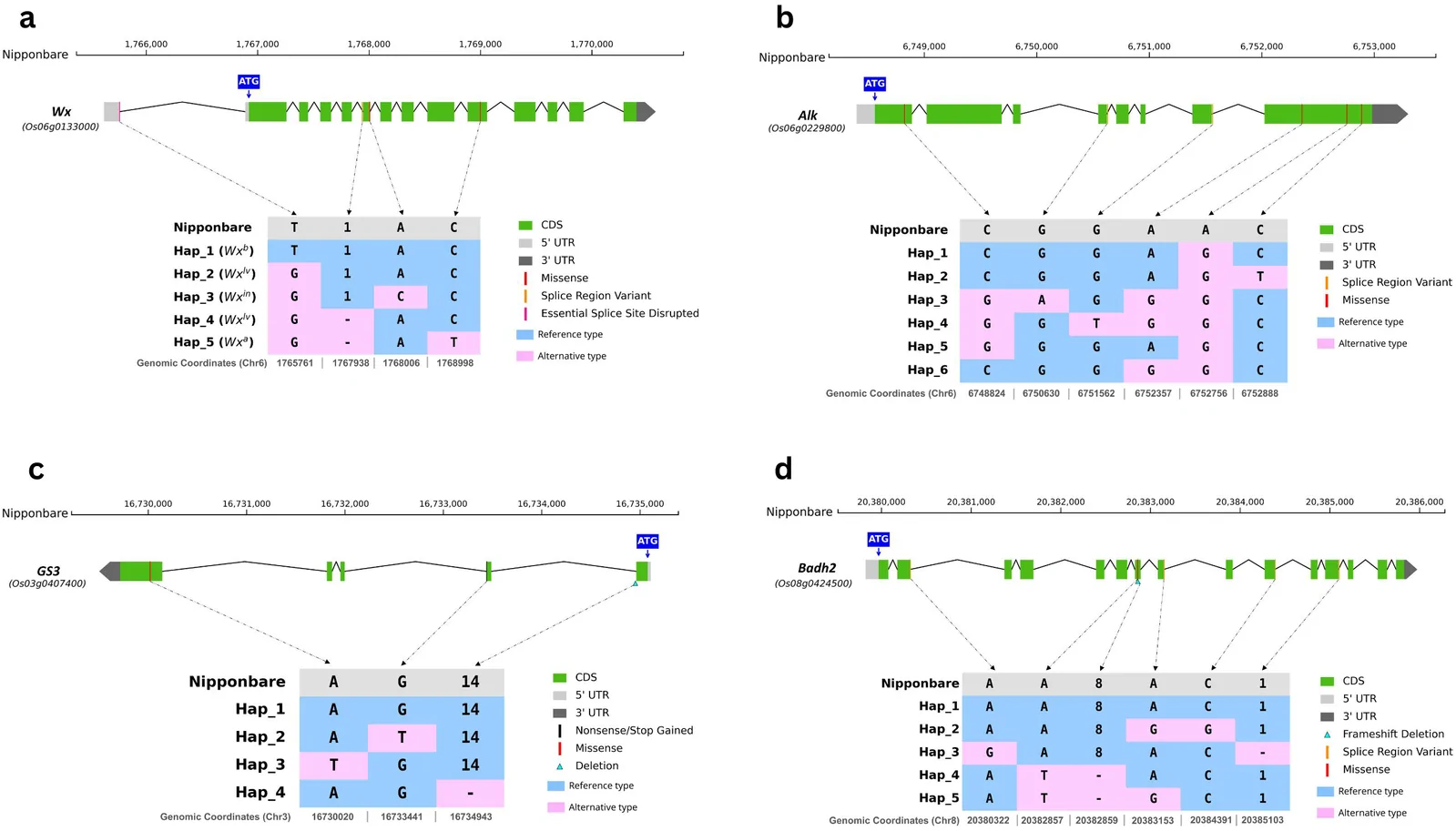
Gene structure and haplotype analysis of key grain quality genes across *Mushk Budji* and reference genotypes. The structure and haplotype variations of key grain-quality genes are shown for **(A)** *Wx (Os06g0133000)*; **(B)** *Alk (Os06g0229800)*; **(C)** *GS3 (Os03g0407400)*; and **(D)** *Badh2 (Os08g0424000)*. Exons are represented by green bars (coding sequences, CDS), while untranslated regions (5’ UTR and 3’ UTR) are shown in light and dark gray, respectively. Functional and structural variants (mis-sense mutations, truncation, splice site variants and frameshift/InDels) are represented along the genome ruler based on the Nipponbare reference genome (IRGSP-1.0). Haplotypes are delineated for *Mushk Budji* and reference genotypes (refer text).

As regards the evolutionary signatures, *SS1* showed relatively high nucleotide diversity (π = 0.004962) and a strongly positive Tajima’s D value (3.575900). In contrast, *GS3* and *Badh2*, controlling grain length and aroma, exhibited near-neutral to negative Tajima’s D values and absence of synonymous substitutions (Ks = 0), indicating fixation of favorable alleles through intensive human selection. The *Alk* and *Wx* genes displayed Ka/Ks ratios of 0.319860 and 0.300901, respectively (**Supplementary Table 10**).

## Discussion

The present study is the first-hand report of *de novo* Genome Assembly of a popular short-grained aromatic landrace *Mushk Budji*. The total assembly size of 378.29 Mb was achieved through WGS performed using Illumina NovaSeq 6000 platform. The contiguous assembly with relatively low number of sequences and high N50 value suggested the effective resolution of repetitive regions and long-range genomic structure, which is often challenging in *de novo* assemblies of plant genomes. The shortest scaffold length of 200 bp reflected stringent quality filtering, reducing fragmentation and assembly artefacts.

The initially higher number of predicted protein-coding genes in *Mushk Budji* signified the underlying genome complexity relative to reference *japonica* cultivars, likely arising from landrace-specific evolutionary processes such as gene duplication, paralog expansion and retention of allelic diversity. The reduction to 43,434 non-redundant genes after clustering pronounced that a proportion of the initial predictions represented a common feature of *de novo* annotations, yet retaining a gene count consistent with *Oryza sativa*. This pattern reflects the possible expansion of gene families linked to environmental adaptation and stress responsiveness, aligning with the ecological and traditional cultivation context of *Mushk Budji*. Furthermore, gene structure metrics, including total CDS length, average gene length, and CDS N50, fall within expected ranges for rice genomes. The high number of CDS (201,003) and exons (211,732) pointed to complex gene architectures with extensive exon-intron organization and were consistent with the previously characterized rice genomes (Ma et al., 2020). The exon and CDS numbers were comparable to those reported for indica cultivars, suggesting conservation of core gene structures across subspecies. These values closely match to those observed in high-quality rice reference genomes, supporting the accuracy of gene model prediction (Fuentes et al., 2019). The presence of long coding sequences (maximum length 16.9 kb) further indicated successful annotation of full-length genes rather than fragmented models. The GO distribution pattern observed in *Mushk Budji* closely mirrors those reported for reference rice genomes, indicating conservation of core biological functions despite the landrace origin of this genome. The presence of genes across essential metabolic and regulatory pathways confirms that the *Mushk Budji* genome retains a complete functional repertoire characteristic of cultivated rice, while providing a foundation for identifying unique variants associated with aroma, grain quality, and temperate adaptation. Collectively, the assembly and annotation metrics demonstrates a high-quality annotation that captures both conserved genomic features and distinctive attributes of this landrace, underscoring its potential significance for functional genomics and crop improvement.

The high proportion of complete and predominantly single-copy BUSCOs indicates excellent structural integrity and minimal gene space loss. The very low fraction of fragmented and missing orthologs further supports the accuracy and contiguity of the assembly. Overall, the BUSCO metrics confirm that the *Mushk Budji* genome assembly is highly complete and comparable to reference-quality *Oryza sativa* genomes (>95% completeness), providing a robust foundation for downstream functional, comparative and evolutionary genomic analyses. The dominance of LTR retrotransposons mirrors the repeat landscape observed in *japonica* and *indica* reference genomes. DNA transposons, including CACTA, hAT, and Mutator superfamilies, were also detected, along with smaller proportions of LINEs, SINEs and simple sequence repeats.

Whole-genome alignments against the Nipponbare baseline typically have yielded the predictable levels of variation for standard *japonica* lines, featured by 132,462 SNPs, 16,448 insertions and 19,318 deletions in the *japonica* landrace *Omachi* (Arai-Kichise et al., 2011) and the 420,475 SNPs and 95,624 InDels observed in the high-yielding *japonica* variety *Longdao 24* (Jiang et al., 2017). In the present context, *Mushk Budji* exhibited higher polymorphisms, although that a portion of this increased diversity can be attributed to the deep sequencing depth employed here to that of the aforementioned studies. The higher sequencing depth inherently increases variant detection sensitivity but cannot solely account for high genomic divergence. Instead, high variation indicates that *Mushk Budji* is not a typical *japonica*. Phenotypically, the landrace presented classical temperate *japonica* signatures, such as cold tolerance, short and bold grain dimensions and moderately sticky cooked rice, yet it simultaneously displayed distinct non-*japonica* features. Contextually, the high magnitude of structural and nucleotide variation identified for *Mushk Budji* shall serve as a genomic fingerprint, where it can be concluded that the landrace is evolutionary distinct compared to that of canonical *japonica* reference. This was also revealed in some of our previous studies on comparative genomics for a temperate germplasm panel (Shikari et al., 2021).

Functional characterization and annotation helped to shortlist the genes that participate in several important pathways related to grain quality, aroma, metabolism and stress responses in *Mushk Budji* rice. A substantial proportion of predicted genes were successfully annotated using GO, KEGG and EC classifications, indicating the functional richness of the genome. Similar large-scale functional annotations have been reported in rice and other plant genomes, emphasizing the importance of integrative databases in deciphering gene functions (Kanehisa et al., 2021; UniProt Consortium, 2023).

The dominance of genes involved in primary metabolic processes, including carbohydrate, amino acid and lipid metabolism, underscores their central role in determining grain quality traits in *Mushk Budji*. In particular, the identification of starch biosynthesis-related enzymes such as starch synthase confirms the genetic basis of grain texture and cooking quality, amylose content and gelatinization behavior (Smith et al., 1997; Tetlow, 2004). Lycopene beta cyclase is involved in carotenoid biosynthesis by converting lycopene into β-carotene, contributing to pigment formation and nutritional quality (Cunningham and Gantt, 1998; Hirschberg, 2001). Aspartate kinase functions as a key regulatory enzyme in lysine and threonine biosynthesis, influencing amino acid composition and nutritional value (Azevedo et al., 1997; Curien et al., 2009). Triosephosphate isomerase genes identified here are expected to play a central role in glycolysis by facilitating the interconversion of dihydroxyacetone phosphate and glyceraldehyde-3-phosphate, thereby contributing to cellular energy production and carbon metabolism (Plaut and Knowles, 1972). Ribokinase participates in the pentose phosphate pathway by phosphorylating ribose and providing intermediates for nucleotide synthesis (Sigrell et al., 1998). Aroma in aromatic rice varieties such as *Mushk Budji* is a complex trait regulated by multiple biochemical pathways. The present findings are consistent with previous studies reporting that rice aroma is governed by a wide range of volatile organic compounds (VOCs), including aldehydes, alcohols, hydrocarbons, esters and heterocyclic compounds (Fayaz et al., 2024). More than 200 volatile compounds have been reported to contribute to rice aroma, with 2-acetyl-1-pyrroline (2-AP) being the principal compound responsible for fragrance (Bradbury et al., 2005; Fitzgerald et al., 2008. In the current study, the identification of genes such as linalool synthase and lipoxygenase further supports the role of terpenoid and lipid-derived pathways in the formation of the volatile terpene compound linalool, influencing the aroma (Wakte et al., 2017). Lipid oxidation pathways are known to generate aldehydes and alcohols such as hexanal and nonanal, which contribute significantly to rice flavor. The presence of these compounds has been detected recently in *Mushk Budji* (Fayaz et al., 2024) and was validated through functional annotation during the present investigation. This supports the idea that aroma is a polygenic and environmentally regulated trait. In addition, E3 ubiquitin protein ligase regulates ubiquitin-mediated protein degradation and stress signaling pathways (Cutler et al., 2010). Sucrose synthase also plays a key role in starch and sucrose metabolism, contributing to carbon partitioning and grain filling during seed development Smith et al., 1997: Tetlow, 2004). Collectively, these fully characterized genes highlight the diverse metabolic and regulatory pathways underlying grain quality, aroma formation and stress adaptation in *Mushk Budji* rice. In addition to quality traits, several genes identified in this study are involved in secondary metabolite biosynthesis, including phenylpropanoid, carotenoid, and flavonoid pathways. Enzymes such as phenylalanine ammonia-lyase plays a central role in the phenylpropanoid pathway responsible for the synthesis of lignin and defense compounds (Vogt, 2010). Chalcone synthase play key roles in the production of compounds associated with plant defense, pigmentation, and stress tolerance (Dixon and Paiva, 1995). Lipoxygenase is involved in lipid oxidation and generates signaling molecules associated with plant defense and stress responses. Hormonal pathways, particularly those involving abscisic acid, play critical roles in stress tolerance and grain development (Cutler et al., 2010). Furthermore, catalase detoxifies reactive oxygen species by decomposing hydrogen peroxide, protecting cells from oxidative stress, while MAP kinase functions in signaling pathways regulating plant responses to various biotic and abiotic stresses.

The integration of metabolic and regulatory genes highlights the complexity of gene networks underlying agronomically important traits. The shortlisting of 134 functionally important genes with complete annotation provides a valuable resource for future functional and breeding studies. These genes represent key components of metabolic and regulatory pathways and may serve as potential targets for improving rice quality traits. Overall, this study demonstrates that the *Mushk Budji* genome harbors a diverse set of genes involved in aroma formation, starch biosynthesis, and stress adaptation, providing a strong foundation for future genomic research and crop improvement.

Allele mining and haplotype analyses helped to explain the genetic architecture that contextually places *Mushk Budji* with respect to both long and short-grained traditional aromatic germplasm of India. Fundamentally, it shares the 8-bp deletion in the *Badh2* gene found in both long-grained Basmati and short-grained aromatic types like, Gobindobhog. It harbors *Wx^in^* and *GS3* Hap_1 allele, which corresponds to starch property and short-grained phenotype. However, at *SSI* locus, *Mushk Budji* (Hap_1) resembles to that of temperate japonica variety, *Koshihikari* and differs from aromatic rice of India (Hap_2). Interestingly, the haplotype distinguishes *Mushk Budji* from short-grained aromatic rice, Gobindobhog, Katarni Rice and Sonasal and long-grained Pusa Basmati 1121, Karnal Local and Type 3 Basmati (with Hap_2). The locus relates to starch biosynthesis and thermal cooking properties and starch fine structure. A similar genetic pattern was observed for *RAmy1A* locus, where *Mushk Budji* possessed the favorable ‘T’ allele at -1947 bp promoter position (as that of *Koshihikari*) but largely absent in the broader pool of traditional Indian aromatic rice. The locus systematically reduces Peak and Setback Viscosity in the RVA profile (Cai et al., 2025). This translates directly to a significantly higher Taste Value (TV), yielding a softer, more cohesive cooked grain. The pasting properties (Peak Viscosity and Taste Value) of *Mushk Budji* seemed to be dependent on favorable ‘T’ allele at *RAmy1A* l). The *Wx^in^*, *Alk^c^*, universal *Badh2*, *SSI* and *RAmy1*, collectively confer a premium Eating and Cooking Quality (ECQ) (Cai et al., 2025) in a premium quality*, Mushk Budji*.

The predominantly positive Tajima’s D values suggest deviations from neutral evolution. While the observed patterns may be consistent with balancing selection, population structure, or demographic history, they do not provide conclusive evidence for these processes for the current study. The varying Tajima’s D and Ka/Ks ratios broadly suggest the influence of multiple functional and evolutionary processes shaping diversity in rice quality-related genes (**Supplementary Table 10**). However, these inferences should be interpreted cautiously because of the relatively limited sample size. Validation using larger and more diverse populations to strengthen these inferences, would be beyond the scope of this work as our focus moves around characterization of landrace, *Mushk Budji*.

The in-depth genome assembly, functional annotation and comparative genomics analyses revealed distinct allelic signatures associated with aroma, grain quality, starch metabolism and environmental adaptation. The identification of key quality-related haplotypes at loci such as *Badh2*, *Wx, Alk, SSI*, and *RAmy1A* highlight the genetic basis underlying the premium eating and cooking quality of *Mushk Budji*. Furthermore, the observed evolutionary divergence from canonical *japonica* references emphasizes its unique genomic identity and valuable breeding potential. Overall, the genomics resource generated in this study provides an important foundation for conservation, functional genomics, molecular breeding and the future genetic improvement of high-value aromatic rice adapted to temperate agro-ecological conditions.

## Data Availability

The original contributions presented in the study are publicly available. This data can be found here: NCBI GenBank, accession JCEPHQ000000000.1.
