# Supplementary material for: Genome sequencing and de novo assembly elucidate the genetic basis of grain quality and fragrance in heirloom rice cultivar Mushk Budji
Source: Front Plant Sci. 2026 Oct 14;17:1896265. doi: 10.3389/fpls.2026.1896265 (PMC13713480; doi:10.3389/fpls.2026.1896265)
Supplement: Supplementary file 1 [file Table3.docx]

**Genome sequencing and *de novo* assembly elucidate the genetic basis of grain quality and fragrance in heirloom** **rice cultivar *Mushk Budji***

**Raheel Shafeeq Khan^1^, Noor-ul-Ain^1^, V. Shasi Kiran Reddy^1^, Ishrat Rafiq^1^, Gazala H. Khan^2^, Fayaz Mohiddin^2^, Sajad M. Zargar^3^, Syed Zameer Hussain^4^, Bashir A. Alie^5^, Najeeb-ul-Rehman Sofi^2^, Asif Bashir Shikari^1^**

^1^Division of Genetics & Plant Breeding, Sher-e-Kashmir University of Agricultural Sciences & Technology of Kashmir, Wadura Campus, J&K, India, 193201

^2^Mountain Research Centre for Field Crops, Sher-e Kashmir University of Agricultural Sciences and Technology of Kashmir, Khudwani Campus, J&K, India, 192101

^3^Division of Biotechnology, Sher-e Kashmir University of Agricultural Sciences and Technology of Kashmir, Shalimar Campus, J&K, India, 190025

^4^Division of Food Science and Technology, Sher-e Kashmir University of Agricultural Sciences and Technology of Kashmir, Shalimar Campus, J&K, India, 190025

^5^Division of Agronomy, Sher-e Kashmir University of Agricultural Sciences and Technology of Kashmir, Wadura Campus, J&K, India, 193201

Corresponding author: [asifshikari@skuastkashmir.ac.in](mailto:asifshikari@skuastkashmir.ac.in)

**Supplementary Table S3. Chromosome-wise Pearsons correlation between number of SNPs and number of InDels per 100 kb window**

| **chromosome** | **pearson_r** | **pearson_p** |
| --- | --- | --- |
| **Chr1** | 0.878782554 | 3.1E-140 |
| **Chr2** | 0.834927509 | 1.3E-94 |
| **Chr3** | 0.83521686 | 4.88E-96 |
| **Chr4** | 0.738569987 | 2.07E-62 |
| **Chr5** | 0.872002652 | 3.81E-94 |
| **Chr6** | 0.856582972 | 4.43E-91 |
| **Chr7** | 0.874405571 | 2.43E-94 |
| **Chr8** | 0.866790687 | 3.33E-87 |
| **Chr9** | 0.803129535 | 3.47E-53 |
| **Chr10** | 0.859868545 | 4.24E-69 |
| **Chr11** | 0.858856643 | 1.15E-85 |
| **Chr12** | 0.866618236 | 2.09E-84 |
