# Supplementary material for: Genome sequencing and de novo assembly elucidate the genetic basis of grain quality and fragrance in heirloom rice cultivar Mushk Budji
Source: Front Plant Sci. 2026 Oct 14;17:1896265. doi: 10.3389/fpls.2026.1896265 (PMC13713480; doi:10.3389/fpls.2026.1896265)
Supplement: Supplementary file 2 [file Table4.docx]

**Genome sequencing and *de novo* assembly elucidate the genetic basis of grain quality and fragrance in heirloom** **rice cultivar *Mushk Budji***

**Raheel Shafeeq Khan^1^, Noor-ul-Ain^1^, V. Shasi Kiran Reddy^1^, Ishrat Rafiq^1^, Gazala H. Khan^2^, Fayaz Mohiddin^2^, Sajad M. Zargar^3^, Syed Zameer Hussain^4^, Bashir A. Alie^5^, Najeeb-ul-Rehman Sofi^2^, Asif Bashir Shikari^1^**

^1^Division of Genetics & Plant Breeding, Sher-e-Kashmir University of Agricultural Sciences & Technology of Kashmir, Wadura Campus, J&K, India, 193201

^2^Mountain Research Centre for Field Crops, Sher-e Kashmir University of Agricultural Sciences and Technology of Kashmir, Khudwani Campus, J&K, India, 192101

^3^Division of Biotechnology, Sher-e Kashmir University of Agricultural Sciences and Technology of Kashmir, Shalimar Campus, J&K, India, 190025

^4^Division of Food Science and Technology, Sher-e Kashmir University of Agricultural Sciences and Technology of Kashmir, Shalimar Campus, J&K, India, 190025

^5^Division of Agronomy, Sher-e Kashmir University of Agricultural Sciences and Technology of Kashmir, Wadura Campus, J&K, India, 193201

Corresponding author: [asifshikari@skuastkashmir.ac.in](mailto:asifshikari@skuastkashmir.ac.in)

**Supplementary Table S4. Functionally annotated genes with complete Gene Ontology classification, EC numbers and KEGG pathway assignments in the Mushk Budji genome**

| **genes** | **Refseq ID** | **Entry** | **Protein names** | **EC number** | **Gene Ontology (biological process)** | **Gene Ontology (cellular component)** | **Gene Ontology (molecular function)** | **Gene Ontology IDs** | **KEGG_Pathways** |
| --- | --- | --- | --- | --- | --- | --- | --- | --- | --- |
| Chr1.g26.t1 | NP_001384925.1 | Q9FTZ2 | Probable 3-beta-hydroxysteroid-Delta(8),Delta(7)-isomerase (EC 5.3.3.5) (Cholestenol Delta-isomerase) (Delta(8)-Delta(7) sterol isomerase) (D8-D7 sterol isomerase) | 5.3.3.5 | sterol biosynthetic process [GO:0016126] | endoplasmic reticulum [GO:0005783]; endoplasmic reticulum membrane [GO:0005789] | C-8 sterol isomerase activity [GO:0000247]; cholestenol delta-isomerase activity [GO:0047750]; steroid Delta-isomerase activity [GO:0004769] | GO:0000247; GO:0004769; GO:0005783; GO:0005789; GO:0016126; GO:0047750 | Metabolic pathways; Biosynthesis of secondary metabolites; Steroid biosynthesis; |
| Chr1.g359.t1 | NP_001393157.1 | P48494 | Triosephosphate isomerase, cytosolic (TIM) (Triose-phosphate isomerase) (EC 5.3.1.1) | 5.3.1.1 | gluconeogenesis [GO:0006094]; glyceraldehyde-3-phosphate biosynthetic process [GO:0046166]; glycerol catabolic process [GO:0019563]; glycolytic process [GO:0006096] | cytosol [GO:0005829] | triose-phosphate isomerase activity [GO:0004807] | GO:0004807; GO:0005829; GO:0006094; GO:0006096; GO:0019563; GO:0046166 | Metabolic pathways; Biosynthesis of secondary metabolites; Microbial metabolism in diverse environments; Carbon metabolism; Biosynthesis of amino acids; Glycolysis_Gluconeogenesis; Fructose and mannose metabolism; Inositol phosphate metabolism; Carbon fixation by Calvin cycle; |
| Chr1.g1270.t1 | XP_015610620.1 | B9EV56 | NEDD8-activating enzyme E1 catalytic subunit (EC 6.2.1.64) | 6.2.1.64 | protein neddylation [GO:0045116] | cytoplasm [GO:0005737]; nucleus [GO:0005634] | ATP binding [GO:0005524]; NEDD8 activating enzyme activity [GO:0019781] | GO:0005524; GO:0005634; GO:0005737; GO:0019781; GO:0045116 | Ubiquitin mediated proteolysis; |
| Chr1.g1332.t1 | XP_015622109.1 | Q9SDJ2 | Magnesium-protoporphyrin IX monomethyl ester [oxidative] cyclase (Mg-protoporphyrin IX monomethyl ester oxidative cyclase) (EC 1.14.13.81) | 1.14.13.81 | chlorophyll biosynthetic process [GO:0015995]; chloroplast organization [GO:0009658]; light-independent chlorophyll biosynthetic process [GO:0036068]; photosynthesis [GO:0015979]; regulation of tetrapyrrole metabolic process [GO:1901401] | chloroplast inner membrane [GO:0009706]; chloroplast thylakoid membrane [GO:0009535] | iron ion binding [GO:0005506]; magnesium-protoporphyrin IX monomethyl ester (oxidative) cyclase activity [GO:0048529]; mRNA binding [GO:0003729] | GO:0003729; GO:0005506; GO:0009535; GO:0009658; GO:0009706; GO:0015979; GO:0015995; GO:0036068; GO:0048529; GO:1901401 | Metabolic pathways; Biosynthesis of secondary metabolites; Porphyrin metabolism; |
| Chr1.g1394.t1 | BAD81569.1 | Q9AR38 | Protoporphyrinogen oxidase, chloroplastic (PPO) (EC 1.3.3.4) (Protoporphyrinogen oxidase 1) | 1.3.3.4 | chlorophyll biosynthetic process [GO:0015995]; protoporphyrinogen IX biosynthetic process [GO:0006782] | chloroplast [GO:0009507] | flavin adenine dinucleotide binding [GO:0050660]; oxygen-dependent protoporphyrinogen oxidase activity [GO:0004729] | GO:0004729; GO:0006782; GO:0009507; GO:0015995; GO:0050660 | Metabolic pathways; Biosynthesis of secondary metabolites; Biosynthesis of cofactors; Porphyrin metabolism; |
| Chr1.g2356.t1 | XP_015642682.1 | Q8LQT7 | 3-ketoacyl-CoA synthase (EC 2.3.1.-) | 2.3.1.- | fatty acid biosynthetic process [GO:0006633] | membrane [GO:0016020] | acyltransferase activity, transferring groups other than amino-acyl groups [GO:0016747] | GO:0006633; GO:0016020; GO:0016747 | Metabolic pathways; Biosynthesis of secondary metabolites; Fatty acid elongation; Plant-pathogen interaction; |
| Chr1.g2743.t1 | XP_015622198.1 | Q8LJ81 | lycopene beta-cyclase (EC 5.5.1.19) | 5.5.1.19 | carotene biosynthetic process [GO:0016120]; xanthophyll biosynthetic process [GO:0016123] | membrane [GO:0016020]; plastid [GO:0009536] | lycopene epsilon cyclase activity [GO:0045435]; oxidoreductase activity, acting on paired donors, with incorporation or reduction of molecular oxygen [GO:0016705] | GO:0009536; GO:0016020; GO:0016120; GO:0016123; GO:0016705; GO:0045435 | Metabolic pathways; Biosynthesis of secondary metabolites; Carotenoid biosynthesis; |
| Chr1.g3306.t1 | XP_015619662.1 | Q0JKL8 | Ribokinase (RK) (EC 2.7.1.15) | 2.7.1.15 | D-ribose catabolic process [GO:0019303]; nucleoside metabolic process [GO:0009116] | cytosol [GO:0005829]; nucleus [GO:0005634]; plastid nucleoid [GO:0042646] | ATP binding [GO:0005524]; metal ion binding [GO:0046872]; ribokinase activity [GO:0004747] | GO:0004747; GO:0005524; GO:0005634; GO:0005829; GO:0009116; GO:0019303; GO:0042646; GO:0046872 | Metabolic pathways; Pentose phosphate pathway; |
| Chr1.g3492.t1 | XP_015621950.1 | Q8LJJ2 | threonine synthase (EC 4.2.3.1) | 4.2.3.1 | cysteine biosynthetic process [GO:0019344]; threonine biosynthetic process [GO:0009088] | chloroplast [GO:0009507]; cytoplasm [GO:0005737] | pyridoxal phosphate binding [GO:0030170]; threonine synthase activity [GO:0004795] | GO:0004795; GO:0005737; GO:0009088; GO:0009507; GO:0019344; GO:0030170 | Metabolic pathways; Biosynthesis of secondary metabolites; Microbial metabolism in diverse environments; Biosynthesis of amino acids; Glycine, serine and threonine metabolism; Vitamin B6 metabolism; |
| Chr1.g4394.t1 | XP_015651015.1 | Q5QLT9 | E3 ubiquitin-protein ligase RMA (EC 2.3.2.27) (Protein RING membrane-anchor) (RING-type E3 ubiquitin transferase RMA) | 2.3.2.27 | ERAD pathway [GO:0036503]; protein ubiquitination [GO:0016567]; ubiquitin-dependent protein catabolic process [GO:0006511] | endoplasmic reticulum membrane [GO:0005789] | ubiquitin protein ligase activity [GO:0061630]; ubiquitin-like protein conjugating enzyme binding [GO:0044390]; zinc ion binding [GO:0008270] | GO:0005789; GO:0006511; GO:0008270; GO:0016567; GO:0036503; GO:0044390; GO:0061630 | Protein processing in endoplasmic reticulum; |
| Chr1.g4441.t1 | BAD73406.1 | Q5QMG6 | UDP-glucuronate decarboxylase (EC 4.1.1.35) | 4.1.1.35 | D-xylose metabolic process [GO:0042732]; UDP-D-xylose biosynthetic process [GO:0033320] | Golgi cisterna membrane [GO:0032580] | NAD+ binding [GO:0070403]; UDP-glucuronate decarboxylase activity [GO:0048040] | GO:0032580; GO:0033320; GO:0042732; GO:0048040; GO:0070403 | Metabolic pathways; Biosynthesis of nucleotide sugars; Amino sugar and nucleotide sugar metabolism; |
| Chr1.g4510.t1 | NP_001393274.1 | Q941T9 | Probable mannose-1-phosphate guanylyltransferase 2 (EC 2.7.7.13) | 2.7.7.13 | GDP-mannose biosynthetic process [GO:0009298]; protein glycosylation [GO:0006486] | cytoplasm [GO:0005737] | GTP binding [GO:0005525]; mannose-1-phosphate guanylyltransferase (GTP) activity [GO:0004475] | GO:0004475; GO:0005525; GO:0005737; GO:0006486; GO:0009298 | Metabolic pathways; Biosynthesis of secondary metabolites; Biosynthesis of nucleotide sugars; Biosynthesis of cofactors; Fructose and mannose metabolism; Amino sugar and nucleotide sugar metabolism; Biosynthesis of various nucleotide sugars; |
| Chr1.g4517.t1 | XP_015622203.1 | Q941T1 | Delta-1-pyrroline-5-carboxylate synthase 2 (OsP5CS2) [Includes: Glutamate 5-kinase (GK) (EC 2.7.2.11) (Gamma-glutamyl kinase); Gamma-glutamyl phosphate reductase (GPR) (EC 1.2.1.41) (Glutamate-5-semialdehyde dehydrogenase) (Glutamyl-gamma-semialdehyde dehydrogenase)] | 1.2.1.41; 2.7.2.11 | L-proline biosynthetic process [GO:0055129]; proline biosynthetic process [GO:0006561] | cytoplasm [GO:0005737] | ATP binding [GO:0005524]; glutamate 5-kinase activity [GO:0004349]; glutamate-5-semialdehyde dehydrogenase activity [GO:0004350] | GO:0004349; GO:0004350; GO:0005524; GO:0005737; GO:0006561; GO:0055129 | Metabolic pathways; Biosynthesis of secondary metabolites; Biosynthesis of amino acids; Arginine and proline metabolism; |
| Chr1.g5072.t1 | XP_015650977.1 | Q5JK45 | aspartate kinase (EC 2.7.2.4) | 2.7.2.4 | lysine biosynthetic process via diaminopimelate [GO:0009089]; threonine biosynthetic process [GO:0009088] | chloroplast [GO:0009507] | aspartate kinase activity [GO:0004072]; ATP binding [GO:0005524] | GO:0004072; GO:0005524; GO:0009088; GO:0009089; GO:0009507 | Metabolic pathways; Biosynthesis of secondary metabolites; Microbial metabolism in diverse environments; 2-Oxocarboxylic acid metabolism; Biosynthesis of amino acids; Glycine, serine and threonine metabolism; Cysteine and methionine metabolism; Lysine biosynthesis; Monobactam biosynthesis; |
| Chr1.g5077.t1 | NP_001395947.1 | Q8RYL1 | Long chain base biosynthesis protein 2b (EC 2.3.1.50) | 2.3.1.50 | ceramide biosynthetic process [GO:0046513]; sphingosine biosynthetic process [GO:0046512] | endoplasmic reticulum membrane [GO:0005789]; serine palmitoyltransferase complex [GO:0017059] | pyridoxal phosphate binding [GO:0030170]; serine C-palmitoyltransferase activity [GO:0004758] | GO:0004758; GO:0005789; GO:0017059; GO:0030170; GO:0046512; GO:0046513 | Metabolic pathways; Sphingolipid metabolism; Sphingolipid signaling pathway; Autophagy - yeast; Cornified envelope formation; |
| Chr1.g5397.t1 | XP_015641142.1 | Q5JNC0 | 2-methoxy-6-polyprenyl-1,4-benzoquinol methylase, mitochondrial (EC 2.1.1.201) (Ubiquinone biosynthesis methyltransferase COQ5) | 2.1.1.201 | methylation [GO:0032259]; ubiquinone biosynthetic process [GO:0006744] | extrinsic component of mitochondrial inner membrane [GO:0031314] | 2-methoxy-6-polyprenyl-1,4-benzoquinol methyltransferase activity [GO:0008425] | GO:0006744; GO:0008425; GO:0031314; GO:0032259 | Metabolic pathways; Biosynthesis of secondary metabolites; Biosynthesis of cofactors; Ubiquinone and other terpenoid-quinone biosynthesis; |
| Chr2.g5423.t1 | XP_015625167.1 | Q6YU90 | glycerate dehydrogenase (EC 1.1.1.29) | 1.1.1.29 | oxidative photosynthetic carbon pathway [GO:0009854] | cytosol [GO:0005829]; peroxisome [GO:0005777] | glyoxylate reductase (NADPH) activity [GO:0030267]; hydroxypyruvate reductase (NADH) activity [GO:0008465]; NAD binding [GO:0051287] | GO:0005777; GO:0005829; GO:0008465; GO:0009854; GO:0030267; GO:0051287 | Metabolic pathways; Biosynthesis of secondary metabolites; Microbial metabolism in diverse environments; Carbon metabolism; Glyoxylate and dicarboxylate metabolism; Glycine, serine and threonine metabolism; |
| Chr2.g5575.t1 | NP_001396182.1 | Q6ZH94 | S-(+)-linalool synthase, chloroplastic (OsLIS) (EC 4.2.3.25) | 4.2.3.25 | defense response to bacterium [GO:0042742]; monoterpene biosynthetic process [GO:0043693]; terpenoid biosynthetic process [GO:0016114] | chloroplast [GO:0009507] | magnesium ion binding [GO:0000287]; S-linalool synthase activity [GO:0034007]; terpene synthase activity [GO:0010333] | GO:0000287; GO:0009507; GO:0010333; GO:0016114; GO:0034007; GO:0042742; GO:0043693 | Biosynthesis of secondary metabolites; Monoterpenoid biosynthesis; |
| Chr2.g5597.t1 | XP_015624834.1 | Q0E4D5 | L-gulonolactone oxidase (EC 1.1.3.8) | 1.1.3.8 | L-ascorbic acid biosynthetic process [GO:0019853] | membrane [GO:0016020] | D-arabinono-1,4-lactone oxidase activity [GO:0003885]; FAD binding [GO:0071949]; L-gulonolactone oxidase activity [GO:0050105] | GO:0003885; GO:0016020; GO:0019853; GO:0050105; GO:0071949 | Metabolic pathways; Biosynthesis of cofactors; Ascorbate and aldarate metabolism; |
| Chr2.g6081.t1 | XP_015626775.1 | Q6H7Q6 | Lipoxygenase (EC 1.13.11.-) | 1.13.11.- | fatty acid biosynthetic process [GO:0006633]; green leaf volatile biosynthetic process [GO:0010597]; lipid oxidation [GO:0034440]; oxylipin biosynthetic process [GO:0031408] | chloroplast [GO:0009507] | linoleate 13S-lipoxygenase activity [GO:0016165]; metal ion binding [GO:0046872]; oxidoreductase activity, acting on single donors with incorporation of molecular oxygen, incorporation of two atoms of oxygen [GO:0016702] | GO:0006633; GO:0009507; GO:0010597; GO:0016165; GO:0016702; GO:0031408; GO:0034440; GO:0046872 | Metabolic pathways; Biosynthesis of secondary metabolites; Linoleic acid metabolism; alpha-Linolenic acid metabolism; |
| Chr2.g7440.t1 | BAD22913.1 | Q6K666 | 3-oxoacyl-[acyl-carrier-protein] reductase (EC 1.1.1.100) | 1.1.1.100 | fatty acid biosynthetic process [GO:0006633] | chloroplast [GO:0009507] | oxidoreductase activity [GO:0016491] | GO:0006633; GO:0009507; GO:0016491 | Metabolic pathways; Biosynthesis of secondary metabolites; Fatty acid metabolism; Biosynthesis of cofactors; Fatty acid biosynthesis; Biotin metabolism; Prodigiosin biosynthesis; |
| Chr2.g7943.t1 | NP_001403620.1 | Q69S81 | Phosphopantothenate--cysteine ligase 1 (EC 6.3.2.5) (Phosphopantothenoylcysteine synthetase 1) (PPC synthetase 1) | 6.3.2.5 | coenzyme A biosynthetic process [GO:0015937] | cytoplasm [GO:0005737]; nucleus [GO:0005634] | phosphopantothenate--cysteine ligase activity [GO:0004632] | GO:0004632; GO:0005634; GO:0005737; GO:0015937 | Metabolic pathways; Biosynthesis of cofactors; Pantothenate and CoA biosynthesis; |
| Chr2.g8217.t1 | XP_015623852.1 | Q6K5Y7 | adenine phosphoribosyltransferase (EC 2.4.2.7) | 2.4.2.7 | adenine salvage [GO:0006168]; AMP salvage [GO:0044209]; purine ribonucleoside salvage [GO:0006166] | cytosol [GO:0005829] | adenine phosphoribosyltransferase activity [GO:0003999] | GO:0003999; GO:0005829; GO:0006166; GO:0006168; GO:0044209 | Metabolic pathways; Nucleotide metabolism; Purine metabolism; |
| Chr2.g8489.t1 | AAQ67235.1 | Q6URQ0 | 3-isopropylmalate dehydratase (EC 4.2.1.33) | 4.2.1.33 | glucosinolate biosynthetic process [GO:0019761] | chloroplast stroma [GO:0009570] | 3-isopropylmalate dehydratase activity [GO:0003861] | GO:0003861; GO:0009570; GO:0019761 | Metabolic pathways; Biosynthesis of secondary metabolites; 2-Oxocarboxylic acid metabolism; Biosynthesis of amino acids; C5-Branched dibasic acid metabolism; Valine, leucine and isoleucine biosynthesis; |
| Chr2.g8795.t1 | NP_001411232.1 | Q05JG2 | Abscisic acid 8'-hydroxylase 1 (ABA 8'-hydroxylase 1) (EC 1.14.14.137) (Cytochrome P450 707A5) (OsABA8ox1) | 1.14.14.137 | abscisic acid catabolic process [GO:0046345]; abscisic acid metabolic process [GO:0009687] | endoplasmic reticulum membrane [GO:0005789] | (+)-abscisic acid 8'-hydroxylase activity [GO:0010295]; heme binding [GO:0020037]; iron ion binding [GO:0005506]; oxidoreductase activity, acting on paired donors, with incorporation or reduction of molecular oxygen, NAD(P)H as one donor, and incorporation of one atom of oxygen [GO:0016709] | GO:0005506; GO:0005789; GO:0009687; GO:0010295; GO:0016709; GO:0020037; GO:0046345 | Biosynthesis of secondary metabolites; Carotenoid biosynthesis; |
| Chr2.g9111.t1 | ACY56190.1 | B8AIG0 | starch synthase (EC 2.4.1.21) | 2.4.1.21 | amylopectin biosynthetic process [GO:0010021]; starch biosynthetic process [GO:0019252] | amyloplast [GO:0009501]; chloroplast [GO:0009507] | alpha-1,4-glucan glucosyltransferase (UDP-glucose donor) activity [GO:0004373] | GO:0004373; GO:0009501; GO:0009507; GO:0010021; GO:0019252 | Metabolic pathways; Biosynthesis of secondary metabolites; Starch and sucrose metabolism; Biofilm formation - Escherichia coli; |
| Chr2.g9112.t1 | BAF10020.2 | Q6Z2T6 | Geranylgeranyl diphosphate reductase, chloroplastic (EC 1.3.1.83) (Geranylgeranyl reductase) | 1.3.1.83 | chlorophyll biosynthetic process [GO:0015995]; photosynthesis [GO:0015979]; vitamin E biosynthetic process [GO:0010189] | chloroplast [GO:0009507] | FAD binding [GO:0071949]; geranylgeranyl diphosphate reductase activity [GO:0102067]; geranylgeranyl reductase activity [GO:0045550] | GO:0009507; GO:0010189; GO:0015979; GO:0015995; GO:0045550; GO:0071949; GO:0102067 | Metabolic pathways; Biosynthesis of secondary metabolites; Porphyrin metabolism; Terpenoid backbone biosynthesis; |
| Chr3.g10170.t1 | XP_015629925.1 | Q8H7Z0 | 3-ketoacyl-CoA synthase (EC 2.3.1.-) | 2.3.1.- | fatty acid biosynthetic process [GO:0006633] | membrane [GO:0016020] | acyltransferase activity, transferring groups other than amino-acyl groups [GO:0016747] | GO:0006633; GO:0016020; GO:0016747 | Metabolic pathways; Biosynthesis of secondary metabolites; Fatty acid elongation; Plant-pathogen interaction; |
| Chr3.g10215.t1 | NP_001388982.1 | Q9ZTP5 | Ribulose-phosphate 3-epimerase, chloroplastic (EC 5.1.3.1) (Pentose-5-phosphate 3-epimerase) (PPE) (R5P3E) (RPE) | 5.1.3.1 | carbohydrate metabolic process [GO:0005975]; pentose-phosphate shunt, non-oxidative branch [GO:0009052]; reductive pentose-phosphate cycle [GO:0019253]; response to cold [GO:0009409]; response to nematode [GO:0009624] | chloroplast thylakoid membrane [GO:0009535]; cytosol [GO:0005829]; stromule [GO:0010319] | D-ribulose-phosphate 3-epimerase activity [GO:0004750]; metal ion binding [GO:0046872] | GO:0004750; GO:0005829; GO:0005975; GO:0009052; GO:0009409; GO:0009535; GO:0009624; GO:0010319; GO:0019253; GO:0046872 | Metabolic pathways; Biosynthesis of secondary metabolites; Microbial metabolism in diverse environments; Carbon metabolism; Biosynthesis of amino acids; Pentose phosphate pathway; Pentose and glucuronate interconversions; Carbon fixation by Calvin cycle; |
| Chr3.g10298.t1 | AAN65442.1 | Q8H7L2 | 3-ketoacyl-CoA synthase (EC 2.3.1.-) | 2.3.1.- | fatty acid biosynthetic process [GO:0006633] | membrane [GO:0016020] | acyltransferase activity, transferring groups other than amino-acyl groups [GO:0016747] | GO:0006633; GO:0016020; GO:0016747 | Metabolic pathways; Biosynthesis of secondary metabolites; Fatty acid elongation; Plant-pathogen interaction; |
| Chr3.g10525.t1 | NP_001404339.1 | B9F676 | Probable glucan 1,3-alpha-glucosidase (EC 3.2.1.207) (Glucosidase II subunit alpha) | 3.2.1.207 | defense response [GO:0006952]; N-glycan processing [GO:0006491]; polysaccharide catabolic process [GO:0000272] | endoplasmic reticulum [GO:0005783] | alpha-glucosidase activity [GO:0090599]; carbohydrate binding [GO:0030246]; Glc2Man9GlcNAc2 oligosaccharide glucosidase activity [GO:0106407] | GO:0000272; GO:0005783; GO:0006491; GO:0006952; GO:0030246; GO:0090599; GO:0106407 | Metabolic pathways; N-Glycan biosynthesis; Protein processing in endoplasmic reticulum; |
| Chr3.g10788.t1 | XP_015628225.1 | Q10NW0 | Imidazole glycerol phosphate synthase hisHF [Includes: Glutaminase (EC 3.5.1.2); Cyclase] | 3.5.1.2 | L-histidine biosynthetic process [GO:0000105] | chloroplast [GO:0009507] | glutaminase activity [GO:0004359]; imidazoleglycerol-phosphate synthase activity [GO:0000107]; lyase activity [GO:0016829] | GO:0000105; GO:0000107; GO:0004359; GO:0009507; GO:0016829 | Metabolic pathways; Biosynthesis of secondary metabolites; Biosynthesis of amino acids; Histidine metabolism; |
| Chr3.g10843.t1 | NP_001389055.1 | Q9XJ29 | Anthranilate synthase alpha subunit 2, chloroplastic (OsASA2) (EC 4.1.3.27) | 4.1.3.27 | L-tryptophan biosynthetic process [GO:0000162] | anthranilate synthase complex [GO:0005950]; chloroplast [GO:0009507] | anthranilate synthase activity [GO:0004049] | GO:0000162; GO:0004049; GO:0005950; GO:0009507 | Metabolic pathways; Biosynthesis of secondary metabolites; Biosynthesis of amino acids; Phenylalanine, tyrosine and tryptophan biosynthesis; Phenazine biosynthesis; Quorum sensing; Biofilm formation - Pseudomonas aeruginosa; |
| Chr3.g10859.t1 | XP_015630469.1 | X5IB44 | phosphopyruvate hydratase (EC 4.2.1.11) | 4.2.1.11 | glycolytic process [GO:0006096] | phosphopyruvate hydratase complex [GO:0000015] | magnesium ion binding [GO:0000287]; phosphopyruvate hydratase activity [GO:0004634] | GO:0000015; GO:0000287; GO:0004634; GO:0006096 | Metabolic pathways; Biosynthesis of secondary metabolites; Microbial metabolism in diverse environments; Carbon metabolism; Biosynthesis of amino acids; Glycolysis_Gluconeogenesis; RNA degradation; HIF-1 signaling pathway; Cytoskeleton in muscle cells; |
| Chr3.g10925.t1 | XP_015632795.1 | Q10NB2 | U-box domain-containing protein 12 (EC 2.3.2.27) (Plant U-box protein 12) (RING-type E3 ubiquitin transferase PUB12) | 2.3.2.27 | cell surface receptor signaling pathway [GO:0007166]; protein ubiquitination [GO:0016567] | cytoplasm [GO:0005737]; nucleus [GO:0005634] | ubiquitin protein ligase activity [GO:0061630] | GO:0005634; GO:0005737; GO:0007166; GO:0016567; GO:0061630 | Plant hormone signal transduction; |
| Chr3.g10953.t1 | NP_001404429.1 | Q10N79 | Arginine biosynthesis bifunctional protein ArgJ, chloroplastic [Cleaved into: Arginine biosynthesis bifunctional protein ArgJ alpha chain; Arginine biosynthesis bifunctional protein ArgJ beta chain] [Includes: Glutamate N-acetyltransferase (GAT) (EC 2.3.1.35) (Ornithine acetyltransferase) (OATase) (Ornithine transacetylase); Amino-acid acetyltransferase (EC 2.3.1.1) (N-acetylglutamate synthase) (AGS)] | 2.3.1.1; 2.3.1.35 | L-arginine biosynthetic process [GO:0006526]; ornithine biosynthetic process [GO:0006592] | chloroplast [GO:0009507] | glutamate N-acetyltransferase activity [GO:0004358]; L-glutamate N-acetyltransferase activity [GO:0004042] | GO:0004042; GO:0004358; GO:0006526; GO:0006592; GO:0009507 | Metabolic pathways; Biosynthesis of secondary metabolites; 2-Oxocarboxylic acid metabolism; Biosynthesis of amino acids; Arginine biosynthesis; |
| Chr3.g11092.t1 | AAR01225.1 | Q10MQ2 | Probable LL-diaminopimelate aminotransferase, chloroplastic (DAP-AT) (DAP-aminotransferase) (LL-DAP-aminotransferase) (EC 2.6.1.83) | 2.6.1.83 | lysine biosynthetic process via diaminopimelate [GO:0009089] | chloroplast [GO:0009507] | L,L-diaminopimelate aminotransferase activity [GO:0010285]; pyridoxal phosphate binding [GO:0030170] | GO:0009089; GO:0009507; GO:0010285; GO:0030170 | Metabolic pathways; Biosynthesis of secondary metabolites; Biosynthesis of amino acids; Lysine biosynthesis; |
| Chr3.g11272.t1 | XP_015629340.1 | Q10LY9 | phosphoglycerate mutase (2,3-diphosphoglycerate-independent) (EC 5.4.2.12) | 5.4.2.12 | carbohydrate metabolic process [GO:0005975]; glucose catabolic process [GO:0006007]; glycolytic process [GO:0006096]; response to blue light [GO:0009637]; response to carbon dioxide [GO:0010037]; stomatal movement [GO:0010118] | cytoplasm [GO:0005737] | manganese ion binding [GO:0030145]; phosphoglycerate mutase activity [GO:0004619] | GO:0004619; GO:0005737; GO:0005975; GO:0006007; GO:0006096; GO:0009637; GO:0010037; GO:0010118; GO:0030145 | Metabolic pathways; Biosynthesis of secondary metabolites; Microbial metabolism in diverse environments; Carbon metabolism; Biosynthesis of amino acids; Glycolysis_Gluconeogenesis; Glycine, serine and threonine metabolism; |
| Chr3.g11895.t1 | NP_001404616.1 | Q84MF1 | Acyl-[acyl-carrier-protein] desaturase 4, chloroplastic (EC 1.14.19.-) | 1.14.19.- | fatty acid biosynthetic process [GO:0006633]; fatty acid metabolic process [GO:0006631] | chloroplast [GO:0009507] | metal ion binding [GO:0046872]; stearoyl-[ACP] desaturase activity [GO:0045300] | GO:0006631; GO:0006633; GO:0009507; GO:0045300; GO:0046872 | Metabolic pathways; Fatty acid metabolism; Fatty acid biosynthesis; Mycolic acid biosynthesis; Biosynthesis of unsaturated fatty acids; |
| Chr3.g12154.t1 | XP_015629041.1 | Q10I64 | L-gulonolactone oxidase (EC 1.1.3.8) | 1.1.3.8 | L-ascorbic acid biosynthetic process [GO:0019853] | membrane [GO:0016020] | D-arabinono-1,4-lactone oxidase activity [GO:0003885]; FAD binding [GO:0071949]; L-gulonolactone oxidase activity [GO:0050105]; oxidoreductase activity [GO:0016491] | GO:0003885; GO:0016020; GO:0016491; GO:0019853; GO:0050105; GO:0071949 | Metabolic pathways; Biosynthesis of cofactors; Ascorbate and aldarate metabolism; |
| Chr3.g13051.t1 | NP_001405434.1 | Q851S8 | Adenylosuccinate synthetase 2, chloroplastic (AMPSase 2) (AdSS 2) (EC 6.3.4.4) (IMP--aspartate ligase 2) | 6.3.4.4 | 'de novo' AMP biosynthetic process [GO:0044208]; IMP metabolic process [GO:0046040] | chloroplast [GO:0009507]; cytoplasm [GO:0005737] | adenylosuccinate synthase activity [GO:0004019]; GTP binding [GO:0005525]; magnesium ion binding [GO:0000287] | GO:0000287; GO:0004019; GO:0005525; GO:0005737; GO:0009507; GO:0044208; GO:0046040 | Metabolic pathways; Nucleotide metabolism; Biosynthesis of cofactors; Purine metabolism; Alanine, aspartate and glutamate metabolism; |
| Chr3.g13154.t1 | ABF98530.1 | Q10DZ5 | Threonine dehydratase (EC 4.3.1.19) (Threonine deaminase) | 4.3.1.19 | isoleucine biosynthetic process [GO:0009097]; L-serine metabolic process [GO:0006563] | chloroplast [GO:0009507] | L-serine ammonia-lyase activity [GO:0003941]; pyridoxal phosphate binding [GO:0030170]; threonine deaminase activity [GO:0004794] | GO:0003941; GO:0004794; GO:0006563; GO:0009097; GO:0009507; GO:0030170 | Metabolic pathways; Biosynthesis of secondary metabolites; Carbon metabolism; Biosynthesis of amino acids; Glycine, serine and threonine metabolism; Valine, leucine and isoleucine biosynthesis; |
| Chr3.g13753.t1 | AAO72379.1 | Q10BX9 | FAD synthetase, chloroplastic (EC 2.7.7.2) (FAD pyrophosphorylase) (FMN adenylyltransferase) (Flavin adenine dinucleotide synthase) | 2.7.7.2 | FAD biosynthetic process [GO:0006747]; riboflavin biosynthetic process [GO:0009231] | chloroplast [GO:0009507] | ATP binding [GO:0005524]; FMN adenylyltransferase activity [GO:0003919] | GO:0003919; GO:0005524; GO:0006747; GO:0009231; GO:0009507 | Metabolic pathways; Biosynthesis of secondary metabolites; Biosynthesis of cofactors; Riboflavin metabolism; |
| Chr3.g13986.t1 | XP_015631709.1 | Q850Z8 | phosphoribosylformylglycinamidine cyclo-ligase (EC 6.3.3.1) (AIR synthase) (Phosphoribosyl-aminoimidazole synthetase) | 6.3.3.1 | 'de novo' IMP biosynthetic process [GO:0006189]; adenine biosynthetic process [GO:0046084]; purine nucleotide biosynthetic process [GO:0006164] | cytosol [GO:0005829] | ATP binding [GO:0005524]; phosphoribosylamine-glycine ligase activity [GO:0004637]; phosphoribosylformylglycinamidine cyclo-ligase activity [GO:0004641] | GO:0004637; GO:0004641; GO:0005524; GO:0005829; GO:0006164; GO:0006189; GO:0046084 | Metabolic pathways; Biosynthesis of secondary metabolites; Purine metabolism; |
| Chr3.g14045.t1 | XP_015632145.1 | Q10AW2 | Ubiquinone biosynthesis monooxygenase COQ6, mitochondrial (EC 1.14.15.45) (2-methoxy-6-polyprenolphenol 4-hydroxylase) (EC 1.14.15.46) | 1.14.15.45; 1.14.15.46 | ubiquinone biosynthetic process [GO:0006744] | extrinsic component of mitochondrial inner membrane [GO:0031314] | 4-hydroxy-3-all-trans-polyprenylbenzoate oxygenase activity [GO:0106364]; FAD binding [GO:0071949]; oxidoreductase activity, acting on paired donors, with incorporation or reduction of molecular oxygen, reduced flavin or flavoprotein as one donor, and incorporation of one atom of oxygen [GO:0016712] | GO:0006744; GO:0016712; GO:0031314; GO:0071949; GO:0106364 | Metabolic pathways; Biosynthesis of secondary metabolites; Biosynthesis of cofactors; Ubiquinone and other terpenoid-quinone biosynthesis; |
| Chr4.g14647.t1 | AAV48542.1 | Q5UJF9 | Cysteine synthase (EC 2.5.1.47) | 2.5.1.47 | cysteine biosynthetic process from serine [GO:0006535] | cytoplasm [GO:0005737] | cysteine synthase activity [GO:0004124] | GO:0004124; GO:0005737; GO:0006535 | Metabolic pathways; Biosynthesis of secondary metabolites; Microbial metabolism in diverse environments; Carbon metabolism; Biosynthesis of amino acids; Sulfur cycle; Sulfur metabolism; Cysteine and methionine metabolism; Cyanoamino acid metabolism; |
| Chr4.g15029.t1 | XP_015637157.1 | Q01LW8 | Glycerol-3-phosphate dehydrogenase (EC 1.1.5.3) | 1.1.5.3 | glycerol-3-phosphate metabolic process [GO:0006072] | mitochondrion [GO:0005739] | glycerol-3-phosphate dehydrogenase (quinone) activity [GO:0004368] | GO:0004368; GO:0005739; GO:0006072 | Metabolic pathways; Biosynthesis of secondary metabolites; Glycerophospholipid metabolism; |
| Chr4.g15113.t1 | XP_015637059.1 | Q7XW88 | Prostaglandin E synthase 2 (EC 5.3.99.3) (Microsomal prostaglandin E synthase 2) | 5.3.99.3 | prostaglandin biosynthetic process [GO:0001516] | endomembrane system [GO:0012505]; membrane [GO:0016020]; mitochondrion [GO:0005739] | prostaglandin-E synthase activity [GO:0050220] | GO:0001516; GO:0005739; GO:0012505; GO:0016020; GO:0050220 | Metabolic pathways; Arachidonic acid metabolism; |
| Chr4.g15971.t1 | XP_015635921.1 | Q7XMI8 | 3-oxoacyl-[acyl-carrier-protein] reductase (EC 1.1.1.100) | 1.1.1.100 | fatty acid biosynthetic process [GO:0006633] | chloroplast [GO:0009507] | 3-oxoacyl-[acyl-carrier-protein] reductase (NADPH) activity [GO:0004316]; NAD binding [GO:0051287] | GO:0004316; GO:0006633; GO:0009507; GO:0051287 | Metabolic pathways; Biosynthesis of secondary metabolites; Fatty acid metabolism; Biosynthesis of cofactors; Fatty acid biosynthesis; Biotin metabolism; Prodigiosin biosynthesis; |
| Chr4.g16174.t1 | XP_015637196.1 | Q0JDF7 | Arogenate dehydratase (EC 4.2.1.91) | 4.2.1.91 | L-phenylalanine biosynthetic process [GO:0009094] | chloroplast [GO:0009507]; chloroplast stroma [GO:0009570]; cytoplasm [GO:0005737] | arogenate dehydratase activity [GO:0047769]; prephenate dehydratase activity [GO:0004664] | GO:0004664; GO:0005737; GO:0009094; GO:0009507; GO:0009570; GO:0047769 | Metabolic pathways; Biosynthesis of secondary metabolites; Biosynthesis of amino acids; Phenylalanine, tyrosine and tryptophan biosynthesis; |
| Chr4.g16303.t1 | XP_015635933.1 | Q01LG0 | Nicotinate phosphoribosyltransferase (EC 6.3.4.21) | 6.3.4.21 | NAD+ biosynthetic process via the salvage pathway [GO:0034355] | cytosol [GO:0005829] | metal ion binding [GO:0046872]; nicotinamide phosphoribosyltransferase activity [GO:0047280]; nicotinate phosphoribosyltransferase activity [GO:0004516] | GO:0004516; GO:0005829; GO:0034355; GO:0046872; GO:0047280 | Metabolic pathways; Biosynthesis of cofactors; |
| Chr4.g16437.t1 | XP_015636353.1 | Q0JCU7 | Zeaxanthin epoxidase, chloroplastic (OsZEP1) (EC 1.14.15.21) (Protein ABA DEFICIENT 1) (OsABA1) | 1.14.15.21 | abscisic acid biosynthetic process [GO:0009688]; multicellular organismal-level water homeostasis [GO:0050891]; response to water deprivation [GO:0009414]; xanthophyll biosynthetic process [GO:0016123] | chloroplast membrane [GO:0031969]; chloroplast thylakoid membrane [GO:0009535] | FAD binding [GO:0071949]; zeaxanthin epoxidase activity [GO:0052662] | GO:0009414; GO:0009535; GO:0009688; GO:0016123; GO:0031969; GO:0050891; GO:0052662; GO:0071949 | Metabolic pathways; Biosynthesis of secondary metabolites; Carotenoid biosynthesis; |
| Chr4.g16495.t1 | XP_015633796.1 | Q01JQ0 | Glutathione hydrolase (EC 2.3.2.2) (EC 3.4.19.13) (Gamma-glutamyltransferase) (Gamma-glutamyltranspeptidase) | 2.3.2.2; 3.4.19.13 | glutathione catabolic process [GO:0006751] | plasma membrane [GO:0005886] | glutathione gamma-glutamylcysteinyltransferase activity [GO:0016756]; glutathione hydrolase activity [GO:0036374] | GO:0005886; GO:0006751; GO:0016756; GO:0036374 | Metabolic pathways; Arachidonic acid metabolism; Taurine and hypotaurine metabolism; Cyanoamino acid metabolism; Glutathione metabolism; |
| Chr4.g16532.t1 | NP_001406332.1 | Q25A90 | anthranilate synthase (EC 4.1.3.27) | 4.1.3.27 | L-tryptophan biosynthetic process [GO:0000162] | anthranilate synthase complex [GO:0005950]; chloroplast [GO:0009507]; cytosol [GO:0005829] | anthranilate synthase activity [GO:0004049] | GO:0000162; GO:0004049; GO:0005829; GO:0005950; GO:0009507 | Metabolic pathways; Biosynthesis of secondary metabolites; Biosynthesis of amino acids; Phenylalanine, tyrosine and tryptophan biosynthesis; Phenazine biosynthesis; Quorum sensing; Biofilm formation - Pseudomonas aeruginosa; |
| Chr4.g16908.t1 | XP_015633843.1 | Q01J56 | low-specificity L-threonine aldolase (EC 4.1.2.48) | 4.1.2.48 | glycine biosynthetic process [GO:0006545]; threonine catabolic process [GO:0006567] | cytosol [GO:0005829] | L-allo-threonine aldolase activity [GO:0008732] | GO:0005829; GO:0006545; GO:0006567; GO:0008732 | Metabolic pathways; Biosynthesis of secondary metabolites; Microbial metabolism in diverse environments; Biosynthesis of amino acids; Glycine, serine and threonine metabolism; |
| Chr4.g16918.t1 | XP_015633952.1 | Q01J47 | Adenosine kinase (AK) (EC 2.7.1.20) (Adenosine 5'-phosphotransferase) | 2.7.1.20 | adenosine salvage [GO:0006169]; AMP salvage [GO:0044209]; purine nucleobase metabolic process [GO:0006144] | cytosol [GO:0005829]; nucleus [GO:0005634] | adenosine kinase activity [GO:0004001]; ATP binding [GO:0005524] | GO:0004001; GO:0005524; GO:0005634; GO:0005829; GO:0006144; GO:0006169; GO:0044209 | Metabolic pathways; Nucleotide metabolism; Purine metabolism; |
| Chr4.g16922.t1 | XP_015633749.1 | Q01J43 | Phenylalanine ammonia-lyase (EC 4.3.1.24) | 4.3.1.24 | cinnamic acid biosynthetic process [GO:0009800]; L-phenylalanine catabolic process [GO:0006559] | cytoplasm [GO:0005737] | phenylalanine ammonia-lyase activity [GO:0045548] | GO:0005737; GO:0006559; GO:0009800; GO:0045548 | Metabolic pathways; Biosynthesis of secondary metabolites; Phenylalanine metabolism; Phenylpropanoid biosynthesis; |
| Chr4.g17099.t1 | XP_015633405.1 | Q01J26 | Mannosyltransferase (EC 2.4.1.-) | 2.4.1.- | protein N-linked glycosylation [GO:0006487] | endoplasmic reticulum membrane [GO:0005789] | dolichyl-P-Man:Man(7)GlcNAc(2)-PP-dolichol alpha-1,6-mannosyltransferase [GO:0052917] | GO:0005789; GO:0006487; GO:0052917 | Metabolic pathways; N-Glycan biosynthesis; Various types of N-glycan biosynthesis; |
| Chr4.g17196.t1 | XP_015637047.1 | Q01HA0 | acyl-CoA hydrolase (EC 3.1.2.20) | 3.1.2.20 | acyl-CoA metabolic process [GO:0006637]; fatty acid catabolic process [GO:0009062] | peroxisomal matrix [GO:0005782] | fatty acyl-CoA hydrolase activity [GO:0047617] | GO:0005782; GO:0006637; GO:0009062; GO:0047617 | Metabolic pathways; Biosynthesis of secondary metabolites; Fatty acid elongation; Biosynthesis of unsaturated fatty acids; Ovarian steroidogenesis; |
| Chr4.g17646.t1 | ACY56214.1 | D0TZS8 | starch synthase (EC 2.4.1.21) | 2.4.1.21 | amylopectin biosynthetic process [GO:0010021]; starch biosynthetic process [GO:0019252] | amyloplast [GO:0009501]; chloroplast [GO:0009507] | alpha-1,4-glucan glucosyltransferase (UDP-glucose donor) activity [GO:0004373]; starch binding [GO:2001070] | GO:0004373; GO:0009501; GO:0009507; GO:0010021; GO:0019252; GO:2001070 | Metabolic pathways; Biosynthesis of secondary metabolites; Starch and sucrose metabolism; Biofilm formation - Escherichia coli; |
| Chr4.g17755.t1 | NP_001389370.1 | Q01HY3 | shikimate kinase (EC 2.7.1.71) | 2.7.1.71 | amino acid biosynthetic process [GO:0008652]; aromatic amino acid family biosynthetic process [GO:0009073]; chorismate biosynthetic process [GO:0009423]; shikimate metabolic process [GO:0019632] | chloroplast [GO:0009507]; cytosol [GO:0005829] | ATP binding [GO:0005524]; metal ion binding [GO:0046872]; shikimate kinase activity [GO:0004765] | GO:0004765; GO:0005524; GO:0005829; GO:0008652; GO:0009073; GO:0009423; GO:0009507; GO:0019632; GO:0046872 | Metabolic pathways; Biosynthesis of secondary metabolites; Biosynthesis of amino acids; Phenylalanine, tyrosine and tryptophan biosynthesis; |
| Chr4.g18013.t1 | BAF16169.2 | Q7XKC8 | Dihydroorotate dehydrogenase (quinone), mitochondrial (DHOdehase) (EC 1.3.5.2) (Dihydroorotate oxidase) | 1.3.5.2 | 'de novo' pyrimidine nucleobase biosynthetic process [GO:0006207]; 'de novo' UMP biosynthetic process [GO:0044205]; pyrimidine ribonucleotide biosynthetic process [GO:0009220] | mitochondrial inner membrane [GO:0005743] | dihydroorotate dehydrogenase (quinone) activity [GO:0106430]; dihydroorotate dehydrogenase activity [GO:0004152] | GO:0004152; GO:0005743; GO:0006207; GO:0009220; GO:0044205; GO:0106430 | Metabolic pathways; Biosynthesis of cofactors; Pyrimidine metabolism; |
| Chr5.g18324.t1 | NP_001407009.1 | Q75IM9 | Isovaleryl-CoA dehydrogenase, mitochondrial (IVD) (EC 1.3.8.4) | 1.3.8.4 | L-leucine catabolic process [GO:0006552] | mitochondrion [GO:0005739] | 3-methylbutanoyl-CoA dehydrogenase activity [GO:0008470]; flavin adenine dinucleotide binding [GO:0050660] | GO:0005739; GO:0006552; GO:0008470; GO:0050660 | Metabolic pathways; Valine, leucine and isoleucine degradation; |
| Chr5.g18890.t1 | XP_015639934.1 | Q0DK00 | alpha-1,2-Mannosidase (EC 3.2.1.-) | 3.2.1.- | carbohydrate metabolic process [GO:0005975]; ERAD pathway [GO:0036503] | endoplasmic reticulum [GO:0005783]; membrane [GO:0016020] | calcium ion binding [GO:0005509]; mannosyl-oligosaccharide 1,2-alpha-mannosidase activity [GO:0004571] | GO:0004571; GO:0005509; GO:0005783; GO:0005975; GO:0016020; GO:0036503 | Metabolic pathways; N-Glycan biosynthesis; Various types of N-glycan biosynthesis; Protein processing in endoplasmic reticulum; |
| Chr5.g18902.t1 | AAT44229.1 | A0A0P0WJ81 | Obtusifoliol 14-alpha demethylase (EC 1.14.14.154) (CYPLI) (Cytochrome P450 51) (Cytochrome P450-LIA1) | 1.14.14.154 | methylation [GO:0032259]; sterol biosynthetic process [GO:0016126] | membrane [GO:0016020] | heme binding [GO:0020037]; iron ion binding [GO:0005506]; methyltransferase activity [GO:0008168]; monooxygenase activity [GO:0004497]; oxidoreductase activity, acting on paired donors, with incorporation or reduction of molecular oxygen [GO:0016705] | GO:0004497; GO:0005506; GO:0008168; GO:0016020; GO:0016126; GO:0016705; GO:0020037; GO:0032259 | Metabolic pathways; Biosynthesis of secondary metabolites; Steroid biosynthesis; |
| Chr5.g19073.t1 | XP_015640384.1 | Q60EU0 | RING-type E3 ubiquitin transferase (EC 2.3.2.27) | 2.3.2.27 | protein ubiquitination [GO:0016567]; ubiquitin-dependent protein catabolic process [GO:0006511] | cytoplasm [GO:0005737] | ubiquitin protein ligase activity [GO:0061630]; zinc ion binding [GO:0008270] | GO:0005737; GO:0006511; GO:0008270; GO:0016567; GO:0061630 | Ubiquitin mediated proteolysis; Wnt signaling pathway; Mitophagy - animal; p53 signaling pathway; |
| Chr5.g19534.t1 | NP_001407229.1 | B9FK36 | Acetyl-CoA carboxylase 2 (EC 6.4.1.2) [Includes: Biotin carboxylase (EC 6.3.4.14)] | 6.3.4.14; 6.4.1.2 | fatty acid biosynthetic process [GO:0006633]; malonyl-CoA biosynthetic process [GO:2001295] | cytosol [GO:0005829] | acetyl-CoA carboxylase activity [GO:0003989]; ATP binding [GO:0005524]; biotin carboxylase activity [GO:0004075]; metal ion binding [GO:0046872] | GO:0003989; GO:0004075; GO:0005524; GO:0005829; GO:0006633; GO:0046872; GO:2001295 | Metabolic pathways; Biosynthesis of secondary metabolites; Microbial metabolism in diverse environments; Fatty acid metabolism; Pyruvate metabolism; Propanoate metabolism; Fatty acid biosynthesis; Aflatoxin biosynthesis; AMPK signaling pathway; Insulin signaling pathway; Glucagon signaling pathway; |
| Chr5.g19725.t1 | XP_015639656.1 | Q5W6F1 | Trans-cinnamate 4-monooxygenase (EC 1.14.14.91) (Cinnamic acid 4-hydroxylase) (C4H) (CA4H) (Cinnamic acid 4-hydroxylase-like protein) (C4H-like) (OsC4HL) (Cytochrome P450 73A33) (Cytochrome P450C4H) | 1.14.14.91 | lignin metabolic process [GO:0009808] | membrane [GO:0016020] | heme binding [GO:0020037]; iron ion binding [GO:0005506]; trans-cinnamate 4-monooxygenase activity [GO:0016710] | GO:0005506; GO:0009808; GO:0016020; GO:0016710; GO:0020037 | Metabolic pathways; Biosynthesis of secondary metabolites; Degradation of aromatic compounds; Ubiquinone and other terpenoid-quinone biosynthesis; Phenylpropanoid biosynthesis; Stilbenoid, diarylheptanoid and gingerol biosynthesis; Flavonoid biosynthesis; |
| Chr5.g19989.t1 | AAV31405.1 | Q60E78 | UDP-glucuronate decarboxylase (EC 4.1.1.35) | 4.1.1.35 | D-xylose metabolic process [GO:0042732]; UDP-D-xylose biosynthetic process [GO:0033320] | Golgi cisterna membrane [GO:0032580] | NAD+ binding [GO:0070403]; UDP-glucuronate decarboxylase activity [GO:0048040] | GO:0032580; GO:0033320; GO:0042732; GO:0048040; GO:0070403 | Metabolic pathways; Biosynthesis of nucleotide sugars; Amino sugar and nucleotide sugar metabolism; |
| Chr5.g20089.t1 | AAT01343.1 | Q2KNB5 | Hexokinase-10 (EC 2.7.1.1) (Hexokinase-7) | 2.7.1.1 | glycolytic process [GO:0006096]; hexose metabolic process [GO:0019318]; intracellular glucose homeostasis [GO:0001678] | chloroplast outer membrane [GO:0009707]; cytosol [GO:0005829]; mitochondrion [GO:0005739] | ATP binding [GO:0005524]; D-glucose binding [GO:0005536]; fructokinase activity [GO:0008865]; glucokinase activity [GO:0004340] | GO:0001678; GO:0004340; GO:0005524; GO:0005536; GO:0005739; GO:0005829; GO:0006096; GO:0008865; GO:0009707; GO:0019318 | Metabolic pathways; Biosynthesis of secondary metabolites; Microbial metabolism in diverse environments; Carbon metabolism; Biosynthesis of nucleotide sugars; Glycolysis_Gluconeogenesis; Fructose and mannose metabolism; Galactose metabolism; Starch and sucrose metabolism; Amino sugar and nucleotide sugar metabolism; Streptomycin biosynthesis; Neomycin, kanamycin and gentamicin biosynthesis; HIF-1 signaling pathway; Insulin signaling pathway; Carbohydrate digestion and absorption; Central carbon metabolism in cancer; Shigellosis; |
| Chr5.g20281.t1 | NP_001389453.1 | P17784 | Fructose-bisphosphate aldolase 1, cytoplasmic (EC 4.1.2.13) (Cytoplasmic aldolase) (cALD) (Gravity-specific protein GSC 233) | 4.1.2.13 | fructose 1,6-bisphosphate metabolic process [GO:0030388]; gluconeogenesis [GO:0006094]; glycolytic process [GO:0006096]; root development [GO:0048364] | cytoplasm [GO:0005737]; cytosol [GO:0005829] | fructose-bisphosphate aldolase activity [GO:0004332] | GO:0004332; GO:0005737; GO:0005829; GO:0006094; GO:0006096; GO:0030388; GO:0048364 | Metabolic pathways; Biosynthesis of secondary metabolites; Microbial metabolism in diverse environments; Carbon metabolism; Biosynthesis of amino acids; Glycolysis_Gluconeogenesis; Pentose phosphate pathway; Fructose and mannose metabolism; Carbon fixation by Calvin cycle; HIF-1 signaling pathway; |
| Chr5.g20618.t1 | NP_001389494.1 | O04226 | Delta-1-pyrroline-5-carboxylate synthase 1 (OsP5CS1) [Includes: Glutamate 5-kinase (GK) (EC 2.7.2.11) (Gamma-glutamyl kinase); Gamma-glutamyl phosphate reductase (GPR) (EC 1.2.1.41) (Glutamate-5-semialdehyde dehydrogenase) (Glutamyl-gamma-semialdehyde dehydrogenase)] | 1.2.1.41; 2.7.2.11 | L-proline biosynthetic process [GO:0055129]; proline biosynthetic process [GO:0006561] | cytoplasm [GO:0005737] | ATP binding [GO:0005524]; glutamate 5-kinase activity [GO:0004349]; glutamate-5-semialdehyde dehydrogenase activity [GO:0004350] | GO:0004349; GO:0004350; GO:0005524; GO:0005737; GO:0006561; GO:0055129 | Metabolic pathways; Biosynthesis of secondary metabolites; Biosynthesis of amino acids; Arginine and proline metabolism; |
| Chr5.g20716.t1 | XP_015639660.1 | Q0DHF0 | E3 ubiquitin-protein ligase RMA (EC 2.3.2.27) (Protein RING membrane-anchor) (RING-type E3 ubiquitin transferase RMA) | 2.3.2.27 | ERAD pathway [GO:0036503]; protein ubiquitination [GO:0016567]; ubiquitin-dependent protein catabolic process [GO:0006511] | endoplasmic reticulum membrane [GO:0005789] | ubiquitin protein ligase activity [GO:0061630]; ubiquitin-like protein conjugating enzyme binding [GO:0044390]; zinc ion binding [GO:0008270] | GO:0005789; GO:0006511; GO:0008270; GO:0016567; GO:0036503; GO:0044390; GO:0061630 | Protein processing in endoplasmic reticulum; |
| Chr5.g21099.t1 | NP_001407676.1 | Q5W676 | Hexokinase-5 (EC 2.7.1.1) (Hexokinase I) | 2.7.1.1 | glucose 6-phosphate metabolic process [GO:0051156]; glucose metabolic process [GO:0006006]; glycolytic process [GO:0006096]; intracellular glucose homeostasis [GO:0001678]; response to glucose [GO:0009749] | chloroplast outer membrane [GO:0009707]; cytosol [GO:0005829]; mitochondrion [GO:0005739] | ATP binding [GO:0005524]; D-glucose binding [GO:0005536]; fructokinase activity [GO:0008865]; glucokinase activity [GO:0004340] | GO:0001678; GO:0004340; GO:0005524; GO:0005536; GO:0005739; GO:0005829; GO:0006006; GO:0006096; GO:0008865; GO:0009707; GO:0009749; GO:0051156 | Metabolic pathways; Biosynthesis of secondary metabolites; Microbial metabolism in diverse environments; Carbon metabolism; Biosynthesis of nucleotide sugars; Glycolysis_Gluconeogenesis; Fructose and mannose metabolism; Galactose metabolism; Starch and sucrose metabolism; Amino sugar and nucleotide sugar metabolism; Streptomycin biosynthesis; Neomycin, kanamycin and gentamicin biosynthesis; HIF-1 signaling pathway; Insulin signaling pathway; Carbohydrate digestion and absorption; Central carbon metabolism in cancer; Shigellosis; |
| Chr5.g21110.t1 | XP_015639708.1 | Q65X97 | ATP-dependent 6-phosphofructokinase (ATP-PFK) (Phosphofructokinase) (EC 2.7.1.11) (Phosphohexokinase) | 2.7.1.11 | fructose 6-phosphate metabolic process [GO:0006002] | cytoplasm [GO:0005737]; cytosol [GO:0005829] | 6-phosphofructokinase activity [GO:0003872]; ATP binding [GO:0005524]; metal ion binding [GO:0046872] | GO:0003872; GO:0005524; GO:0005737; GO:0005829; GO:0006002; GO:0046872 | Metabolic pathways; Biosynthesis of secondary metabolites; Microbial metabolism in diverse environments; Carbon metabolism; Biosynthesis of amino acids; Glycolysis_Gluconeogenesis; Pentose phosphate pathway; Fructose and mannose metabolism; Galactose metabolism; RNA degradation; HIF-1 signaling pathway; AMPK signaling pathway; Glucagon signaling pathway; Central carbon metabolism in cancer; |
| Chr5.g21321.t1 | NP_001389554.1 | Q0DG35 | Glutamate synthase 2 [NADH], chloroplastic (EC 1.4.1.14) (NADH-dependent glutamate synthase 2) (NADH-GOGAT 2) | 1.4.1.14 | ammonia assimilation cycle [GO:0019676]; glutamate biosynthetic process [GO:0006537]; L-glutamate biosynthetic process [GO:0097054] | chloroplast [GO:0009507] | 3 iron, 4 sulfur cluster binding [GO:0051538]; flavin adenine dinucleotide binding [GO:0050660]; FMN binding [GO:0010181]; glutamate synthase (NADH) activity [GO:0016040]; iron ion binding [GO:0005506]; oxidoreductase activity, acting on the CH-NH2 group of donors, NAD or NADP as acceptor [GO:0016639] | GO:0005506; GO:0006537; GO:0009507; GO:0010181; GO:0016040; GO:0016639; GO:0019676; GO:0050660; GO:0051538; GO:0097054 | Metabolic pathways; Biosynthesis of secondary metabolites; Microbial metabolism in diverse environments; Biosynthesis of amino acids; Nitrogen metabolism; Alanine, aspartate and glutamate metabolism; |
| Chr5.g21364.t1 | XP_015637518.1 | Q688Y2 | 3-deoxy-manno-octulosonate cytidylyltransferase (EC 2.7.7.38) (CMP-2-keto-3-deoxyoctulosonic acid synthase) | 2.7.7.38 | CMP-keto-3-deoxy-D-manno-octulosonic acid biosynthetic process [GO:0033468] | membrane [GO:0016020]; mitochondrion [GO:0005739] | 3-deoxy-manno-octulosonate cytidylyltransferase activity [GO:0008690] | GO:0005739; GO:0008690; GO:0016020; GO:0033468 | Metabolic pathways; Biosynthesis of nucleotide sugars; Biosynthesis of various nucleotide sugars; |
| Chr5.g21427.t1 | XP_015633062.1 | Q851Z6 | aspartate kinase (EC 2.7.2.4) | 2.7.2.4 | lysine biosynthetic process via diaminopimelate [GO:0009089]; threonine biosynthetic process [GO:0009088] | chloroplast [GO:0009507] | aspartate kinase activity [GO:0004072]; ATP binding [GO:0005524] | GO:0004072; GO:0005524; GO:0009088; GO:0009089; GO:0009507 | Metabolic pathways; Biosynthesis of secondary metabolites; Microbial metabolism in diverse environments; 2-Oxocarboxylic acid metabolism; Biosynthesis of amino acids; Glycine, serine and threonine metabolism; Cysteine and methionine metabolism; Lysine biosynthesis; Monobactam biosynthesis; |
| Chr6.g21652.t1 | NP_001389605.1 | Q5VRH9 | U-box domain-containing protein 12 (EC 2.3.2.27) (Plant U-box protein 12) (OsPUB12) (RING-type E3 ubiquitin transferase PUB12) | 2.3.2.27 | protein ubiquitination [GO:0016567] | cytoplasm [GO:0005737]; nucleus [GO:0005634] | ubiquitin-protein transferase activity [GO:0004842] | GO:0004842; GO:0005634; GO:0005737; GO:0016567 | Plant hormone signal transduction; |
| Chr6.g21699.t1 | NP_001389608.1 | P41344 | Ferredoxin--NADP reductase, leaf isozyme 1, chloroplastic (FNR) (EC 1.18.1.2) (Leaf FNR 1) (FNR-1) (Os-LFNR1) | 1.18.1.2 | electron transport chain [GO:0022900]; photosynthesis [GO:0015979] | chloroplast stroma [GO:0009570]; chloroplast thylakoid membrane protein complex [GO:0098807] | electron transfer activity [GO:0009055]; ferredoxin-NADP+ reductase activity [GO:0004324] | GO:0004324; GO:0009055; GO:0009570; GO:0015979; GO:0022900; GO:0098807 | Metabolic pathways; Photosynthesis; |
| Chr6.g22366.t1 | NP_001408302.1 | Q69TF4 | Pantothenate kinase 1 (EC 2.7.1.33) (Pantothenic acid kinase 1) | 2.7.1.33 | coenzyme A biosynthetic process [GO:0015937] | cytosol [GO:0005829]; nucleus [GO:0005634] | ATP binding [GO:0005524]; pantothenate kinase activity [GO:0004594] | GO:0004594; GO:0005524; GO:0005634; GO:0005829; GO:0015937 | Metabolic pathways; Biosynthesis of cofactors; Pantothenate and CoA biosynthesis; |
| Chr6.g22535.t1 | BAF19140.2 | Q0DDD3 | fructokinase (EC 2.7.1.4) | 2.7.1.4 | fructose metabolic process [GO:0006000] | cytoplasm [GO:0005737] | fructokinase activity [GO:0008865] | GO:0005737; GO:0006000; GO:0008865 | Metabolic pathways; Biosynthesis of secondary metabolites; Biosynthesis of nucleotide sugars; Fructose and mannose metabolism; Starch and sucrose metabolism; Amino sugar and nucleotide sugar metabolism; |
| Chr6.g22612.t1 | BAD45807.1 | Q654G0 | Alpha-mannosidase (EC 3.2.1.-) | 3.2.1.- | glycoprotein metabolic process [GO:0009100]; mannose metabolic process [GO:0006013]; polysaccharide catabolic process [GO:0000272] | Golgi membrane [GO:0000139] | carbohydrate binding [GO:0030246]; mannosyl-oligosaccharide 1,3-1,6-alpha-mannosidase activity [GO:0004572]; metal ion binding [GO:0046872] | GO:0000139; GO:0000272; GO:0004572; GO:0006013; GO:0009100; GO:0030246; GO:0046872 | Metabolic pathways; N-Glycan biosynthesis; Various types of N-glycan biosynthesis; |
| Chr6.g23371.t1 | XP_015644349.1 | Q5VN31 | 1,4-alpha-glucan branching enzyme (EC 2.4.1.18) | 2.4.1.18 | carbohydrate metabolic process [GO:0005975]; glycogen biosynthetic process [GO:0005978]; starch biosynthetic process [GO:0019252]; starch catabolic process [GO:0005983] | amyloplast [GO:0009501]; cytoplasm [GO:0005737] | 1,4-alpha-glucan branching enzyme activity [GO:0003844]; cation binding [GO:0043169]; hydrolase activity, hydrolyzing O-glycosyl compounds [GO:0004553] | GO:0003844; GO:0004553; GO:0005737; GO:0005975; GO:0005978; GO:0005983; GO:0009501; GO:0019252; GO:0043169 | Metabolic pathways; Biosynthesis of secondary metabolites; Starch and sucrose metabolism; |
| Chr6.g23978.t1 | NP_001408551.1 | Q0DBI8 | Inositol oxygenase (EC 1.13.99.1) (Myo-inositol oxygenase) | 1.13.99.1 | inositol catabolic process [GO:0019310]; L-ascorbic acid biosynthetic process [GO:0019853] | cytoplasm [GO:0005737] | inositol oxygenase activity [GO:0050113]; iron ion binding [GO:0005506] | GO:0005506; GO:0005737; GO:0019310; GO:0019853; GO:0050113 | Metabolic pathways; Biosynthesis of nucleotide sugars; Ascorbate and aldarate metabolism; Inositol phosphate metabolism; |
| Chr6.g24161.t1 | XP_015643955.1 | Q69UF2 | 3-hydroxyisobutyryl-CoA hydrolase (HIB-CoA hydrolase) (HIBYL-CoA-H) (EC 3.1.2.4) (3-hydroxyisobutyryl-coenzyme A hydrolase) | 3.1.2.4 | L-valine catabolic process [GO:0006574] | mitochondrion [GO:0005739] | 3-hydroxyisobutyryl-CoA hydrolase activity [GO:0003860] | GO:0003860; GO:0005739; GO:0006574 | Metabolic pathways; Carbon metabolism; Propanoate metabolism; Valine, leucine and isoleucine degradation; beta-Alanine metabolism; |
| Chr6.g24561.t1 | NP_001408603.1 | Q67W82 | 4-coumarate--CoA ligase 4 (4CL 4) (Os4CL4) (EC 6.2.1.12) ((E)-ferulate--CoA ligase) (EC 6.2.1.34) (4-coumaroyl-CoA synthase 4) (Protein RESISTANCE TO ALUMINUM 1) | 6.2.1.12; 6.2.1.34 | lignin biosynthetic process [GO:0009809]; phenylpropanoid metabolic process [GO:0009698]; response to aluminum ion [GO:0010044] | cytoplasm [GO:0005737] | (E)-caffeate-CoA ligase activity [GO:0106286]; 4-coumarate-CoA ligase activity [GO:0016207]; ATP binding [GO:0005524]; CoA-ligase activity [GO:0016405]; trans-cinnamate-CoA ligase activity [GO:0106290]; trans-feruloyl-CoA synthase activity [GO:0050563] | GO:0005524; GO:0005737; GO:0009698; GO:0009809; GO:0010044; GO:0016207; GO:0016405; GO:0050563; GO:0106286; GO:0106290 | Metabolic pathways; Biosynthesis of secondary metabolites; Ubiquinone and other terpenoid-quinone biosynthesis; Phenylpropanoid biosynthesis; |
| Chr7.g25115.t1 | XP_015646972.1 | Q69UU3 | Alanine aminotransferase 2 (EC 2.6.1.2) (EC 2.6.1.4) (EC 2.6.1.44) | 2.6.1.2; 2.6.1.4; 2.6.1.44 | L-alanine catabolic process [GO:0042853]; photorespiration [GO:0009853] | peroxisome [GO:0005777] | alanine-glyoxylate transaminase activity [GO:0008453]; glycine:2-oxoglutarate aminotransferase activity [GO:0047958]; L-alanine:2-oxoglutarate aminotransferase activity [GO:0004021]; pyridoxal phosphate binding [GO:0030170] | GO:0004021; GO:0005777; GO:0008453; GO:0009853; GO:0030170; GO:0042853; GO:0047958 | Metabolic pathways; Biosynthesis of secondary metabolites; Microbial metabolism in diverse environments; Carbon metabolism; 2-Oxocarboxylic acid metabolism; Biosynthesis of amino acids; Glyoxylate and dicarboxylate metabolism; Carbon fixation by Calvin cycle; Alanine, aspartate and glutamate metabolism; Glycine, serine and threonine metabolism; Arginine biosynthesis; |
| Chr7.g25659.t1 | NP_001408943.1 | Q6YU51 | Probable 1-deoxy-D-xylulose-5-phosphate synthase 2, chloroplastic (1-deoxyxylulose-5-phosphate synthase) (DXP synthase) (DXPS) (EC 2.2.1.7) | 2.2.1.7 | 1-deoxy-D-xylulose 5-phosphate biosynthetic process [GO:0052865]; glyceraldehyde-3-phosphate metabolic process [GO:0019682]; terpenoid biosynthetic process [GO:0016114]; thiamine biosynthetic process [GO:0009228] | chloroplast [GO:0009507] | 1-deoxy-D-xylulose-5-phosphate synthase activity [GO:0008661]; metal ion binding [GO:0046872]; transketolase or transaldolase activity [GO:0016744] | GO:0008661; GO:0009228; GO:0009507; GO:0016114; GO:0016744; GO:0019682; GO:0046872; GO:0052865 | Metabolic pathways; Biosynthesis of secondary metabolites; Thiamine metabolism; Terpenoid backbone biosynthesis; |
| Chr7.g25733.t1 | XP_015644684.1 | Q6Z368 | Squalene synthase (EC 2.5.1.21) | 2.5.1.21 | farnesyl diphosphate metabolic process [GO:0045338] | endoplasmic reticulum membrane [GO:0005789]; plastoglobule [GO:0010287] | 15-cis-phytoene synthase activity [GO:0046905]; D-glucose transmembrane transporter activity [GO:0055056]; squalene synthase [NAD(P)H] activity [GO:0051996] | GO:0005789; GO:0010287; GO:0045338; GO:0046905; GO:0051996; GO:0055056 | Metabolic pathways; Biosynthesis of secondary metabolites; Steroid biosynthesis; Sesquiterpenoid and triterpenoid biosynthesis; |
| Chr7.g25905.t1 | NP_001408997.1 | Q8H2J9 | Glycerol-3-phosphate dehydrogenase [NAD(+)], chloroplastic (EC 1.1.1.8) | 1.1.1.8 | carbohydrate metabolic process [GO:0005975]; glycerol-3-phosphate catabolic process [GO:0046168]; glycerol-3-phosphate metabolic process [GO:0006072]; glycerophospholipid metabolic process [GO:0006650]; phospholipid biosynthetic process [GO:0008654] | chloroplast [GO:0009507]; cytosol [GO:0005829] | glycerol-3-phosphate dehydrogenase (NAD+) activity [GO:0141152]; NAD binding [GO:0051287] | GO:0005829; GO:0005975; GO:0006072; GO:0006650; GO:0008654; GO:0009507; GO:0046168; GO:0051287; GO:0141152 | Metabolic pathways; Biosynthesis of secondary metabolites; Glycerophospholipid metabolism; MAPK signaling pathway - yeast; |
| Chr7.g26235.t1 | XP_015645149.1 | B7F9D1 | phosphatidylinositol N-acetylglucosaminyltransferase (EC 2.4.1.198) (GlcNAc-PI synthesis protein) | 2.4.1.198 | GPI anchor biosynthetic process [GO:0006506]; pollen germination [GO:0009846]; pollen tube growth [GO:0009860] | glycosylphosphatidylinositol-N-acetylglucosaminyltransferase (GPI-GnT) complex [GO:0000506] | phosphatidylinositol N-acetylglucosaminyltransferase activity [GO:0017176] | GO:0000506; GO:0006506; GO:0009846; GO:0009860; GO:0017176 | Metabolic pathways; Glycosylphosphatidylinositol (GPI)-anchor biosynthesis; |
| Chr7.g26740.t1 | NP_001409054.1 | Q8LH82 | Hexokinase-1 (EC 2.7.1.1) | 2.7.1.1 | glucose 6-phosphate metabolic process [GO:0051156]; glucose metabolic process [GO:0006006]; glycolytic process [GO:0006096]; intracellular glucose homeostasis [GO:0001678] | cytosol [GO:0005829]; mitochondrion [GO:0005739] | ATP binding [GO:0005524]; D-glucose binding [GO:0005536]; fructokinase activity [GO:0008865]; glucokinase activity [GO:0004340] | GO:0001678; GO:0004340; GO:0005524; GO:0005536; GO:0005739; GO:0005829; GO:0006006; GO:0006096; GO:0008865; GO:0051156 | Metabolic pathways; Biosynthesis of secondary metabolites; Microbial metabolism in diverse environments; Carbon metabolism; Biosynthesis of nucleotide sugars; Glycolysis_Gluconeogenesis; Fructose and mannose metabolism; Galactose metabolism; Starch and sucrose metabolism; Amino sugar and nucleotide sugar metabolism; Streptomycin biosynthesis; Neomycin, kanamycin and gentamicin biosynthesis; HIF-1 signaling pathway; Insulin signaling pathway; Carbohydrate digestion and absorption; Central carbon metabolism in cancer; Shigellosis; |
| Chr7.g27007.t1 | NP_001409086.1 | Q69PX0 | RING-type E3 ubiquitin transferase (EC 2.3.2.27) | 2.3.2.27 | plant-type hypersensitive response [GO:0009626]; positive regulation of plant-type hypersensitive response [GO:0034052]; protein ubiquitination [GO:0016567]; ubiquitin-dependent protein catabolic process [GO:0006511] | cytosol [GO:0005829]; plasma membrane [GO:0005886]; ubiquitin ligase complex [GO:0000151] | ubiquitin binding [GO:0043130]; ubiquitin protein ligase activity [GO:0061630]; zinc ion binding [GO:0008270] | GO:0000151; GO:0005829; GO:0005886; GO:0006511; GO:0008270; GO:0009626; GO:0016567; GO:0034052; GO:0043130; GO:0061630 | Protein processing in endoplasmic reticulum; Mitophagy - animal; |
| Chr7.g27011.t1 | XP_015644980.1 | Q8H534 | adenine phosphoribosyltransferase (EC 2.4.2.7) | 2.4.2.7 | adenine salvage [GO:0006168]; AMP salvage [GO:0044209]; purine ribonucleoside salvage [GO:0006166] | cytosol [GO:0005829] | adenine phosphoribosyltransferase activity [GO:0003999] | GO:0003999; GO:0005829; GO:0006166; GO:0006168; GO:0044209 | Metabolic pathways; Nucleotide metabolism; Purine metabolism; |
| Chr7.g27880.t1 | AAK55784.1 | Q94LL6 | RING-type E3 ubiquitin transferase (EC 2.3.2.27) | 2.3.2.27 | protein import into peroxisome matrix [GO:0016558] | peroxisomal membrane [GO:0005778] | transferase activity [GO:0016740]; zinc ion binding [GO:0008270] | GO:0005778; GO:0008270; GO:0016558; GO:0016740 | Peroxisome; |
| Chr7.g27968.t1 | NP_001390095.1 | Q0D4J5 | Phospho-2-dehydro-3-deoxyheptonate aldolase (EC 2.5.1.54) | 2.5.1.54 | amino acid biosynthetic process [GO:0008652]; aromatic amino acid family biosynthetic process [GO:0009073]; chorismate biosynthetic process [GO:0009423] | chloroplast [GO:0009507] | 3-deoxy-7-phosphoheptulonate synthase activity [GO:0003849] | GO:0003849; GO:0008652; GO:0009073; GO:0009423; GO:0009507 | Metabolic pathways; Biosynthesis of secondary metabolites; Biosynthesis of amino acids; Phenylalanine, tyrosine and tryptophan biosynthesis; Quorum sensing; |
| Chr7.g28208.t1 | NP_001390160.1 | Q69RJ0 | Ferredoxin-dependent glutamate synthase, chloroplastic (EC 1.4.7.1) (Fd-GOGAT) | 1.4.7.1 | ammonia assimilation cycle [GO:0019676]; glutamate biosynthetic process [GO:0006537]; L-glutamate biosynthetic process [GO:0097054] | chloroplast [GO:0009507] | 3 iron, 4 sulfur cluster binding [GO:0051538]; glutamate synthase (ferredoxin) activity [GO:0016041]; glutamate synthase activity [GO:0015930]; metal ion binding [GO:0046872] | GO:0006537; GO:0009507; GO:0015930; GO:0016041; GO:0019676; GO:0046872; GO:0051538; GO:0097054 | Microbial metabolism in diverse environments; Glyoxylate and dicarboxylate metabolism; Nitrogen metabolism; |
| Chr7.g28225.t1 | XP_015647599.1 | Q7EZZ0 | UDP-N-acetylglucosamine--dolichyl-phosphate N-acetylglucosaminephosphotransferase (EC 2.7.8.15) (GlcNAc-1-P transferase) (N-acetylglucosamine-1-phosphate transferase) | 2.7.8.15 | dolichol-linked oligosaccharide biosynthetic process [GO:0006488] | endoplasmic reticulum membrane [GO:0005789]; membrane [GO:0016020] | glycosyltransferase activity [GO:0016757]; UDP-N-acetylglucosamine-dolichyl-phosphate N-acetylglucosaminephosphotransferase activity [GO:0003975] | GO:0003975; GO:0005789; GO:0006488; GO:0016020; GO:0016757 | Metabolic pathways; N-Glycan biosynthesis; |
| Chr7.g28364.t1 | XP_015644968.1 | B9FUX5 | Hexosyltransferase (EC 2.4.1.-) | 2.4.1.- | cell wall organization [GO:0071555]; pectin biosynthetic process [GO:0045489] | Golgi membrane [GO:0000139] | polygalacturonate 4-alpha-galacturonosyltransferase activity [GO:0047262] | GO:0000139; GO:0045489; GO:0047262; GO:0071555 | Metabolic pathways; Amino sugar and nucleotide sugar metabolism; |
| Chr8.g28593.t1 | NP_001390297.1 | Q9S827 | Succinate dehydrogenase [ubiquinone] iron-sulfur subunit 1, mitochondrial (EC 1.3.5.1) (Iron-sulfur subunit of complex II) (Ip) | 1.3.5.1 | aerobic respiration [GO:0009060]; respiratory electron transport chain [GO:0022904]; tricarboxylic acid cycle [GO:0006099] | mitochondrial inner membrane [GO:0005743]; mitochondrion [GO:0005739]; respiratory chain complex II (succinate dehydrogenase) [GO:0045273] | 2 iron, 2 sulfur cluster binding [GO:0051537]; 3 iron, 4 sulfur cluster binding [GO:0051538]; 4 iron, 4 sulfur cluster binding [GO:0051539]; electron transfer activity [GO:0009055]; metal ion binding [GO:0046872]; succinate dehydrogenase (quinone) activity [GO:0008177] | GO:0005739; GO:0005743; GO:0006099; GO:0008177; GO:0009055; GO:0009060; GO:0022904; GO:0045273; GO:0046872; GO:0051537; GO:0051538; GO:0051539 | Metabolic pathways; Biosynthesis of secondary metabolites; Microbial metabolism in diverse environments; Carbon metabolism; Citrate cycle (TCA cycle); Oxidative phosphorylation; Thermogenesis; Chemical carcinogenesis - reactive oxygen species; Amyotrophic lateral sclerosis; Pathways of neurodegeneration - multiple diseases; |
| Chr8.g29293.t1 | XP_015650484.1 | A0A0P0XD69 | ethanolamine-phosphate cytidylyltransferase (EC 2.7.7.14) (CTP:phosphoethanolamine cytidylyltransferase) | 2.7.7.14 | phosphatidylethanolamine biosynthetic process [GO:0006646] | cytoplasm [GO:0005737]; membrane [GO:0016020] | ethanolamine-phosphate cytidylyltransferase activity [GO:0004306] | GO:0004306; GO:0005737; GO:0006646; GO:0016020 | Metabolic pathways; Glycerophospholipid metabolism; Phosphonate and phosphinate metabolism; |
| Chr8.g29899.t1 | XP_015648591.1 | Q6ZAH2 | 4-hydroxybenzoate polyprenyltransferase, mitochondrial (4-HB polyprenyltransferase) (EC 2.5.1.39) (Para-hydroxybenzoate--polyprenyltransferase) (PHB:PPT) (PHB:polyprenyltransferase) | 2.5.1.39 | isoprenoid biosynthetic process [GO:0008299]; ubiquinone biosynthetic process [GO:0006744] | mitochondrial inner membrane [GO:0005743] | 4-hydroxybenzoate polyprenyltransferase activity [GO:0008412]; transferase activity, transferring alkyl or aryl (other than methyl) groups [GO:0016765] | GO:0005743; GO:0006744; GO:0008299; GO:0008412; GO:0016765 | Metabolic pathways; Biosynthesis of secondary metabolites; Biosynthesis of cofactors; Ubiquinone and other terpenoid-quinone biosynthesis; |
| Chr8.g30041.t1 | NP_001390460.1 | P15280 | Glucose-1-phosphate adenylyltransferase small subunit 2, chloroplastic/amyloplastic/cytosolic (OsAGPS2) (OsAPS2) (EC 2.7.7.27) (ADP-glucose pyrophosphorylase AGPS2) (ADP-glucose synthase AGPS2) (AGPase B) (Alpha-D-glucose-1-phosphate adenyl transferase) (OsAGPS2a) (OsAGPS2b) | 2.7.7.27 | glycogen biosynthetic process [GO:0005978]; starch biosynthetic process [GO:0019252]; starch metabolic process [GO:0005982] | amyloplast [GO:0009501]; chloroplast [GO:0009507]; cytosol [GO:0005829] | ATP binding [GO:0005524]; glucose-1-phosphate adenylyltransferase activity [GO:0008878] | GO:0005524; GO:0005829; GO:0005978; GO:0005982; GO:0008878; GO:0009501; GO:0009507; GO:0019252 | Metabolic pathways; Biosynthesis of secondary metabolites; Biosynthesis of nucleotide sugars; Starch and sucrose metabolism; Amino sugar and nucleotide sugar metabolism; Biofilm formation - Escherichia coli; |
| Chr8.g30873.t1 | XP_015650153.1 | Q6YTT3 | Phospho-2-dehydro-3-deoxyheptonate aldolase (EC 2.5.1.54) | 2.5.1.54 | amino acid biosynthetic process [GO:0008652]; aromatic amino acid family biosynthetic process [GO:0009073]; chorismate biosynthetic process [GO:0009423] | chloroplast [GO:0009507] | 3-deoxy-7-phosphoheptulonate synthase activity [GO:0003849] | GO:0003849; GO:0008652; GO:0009073; GO:0009423; GO:0009507 | Metabolic pathways; Biosynthesis of secondary metabolites; Biosynthesis of amino acids; Phenylalanine, tyrosine and tryptophan biosynthesis; Quorum sensing; |
| Chr8.g31394.t1 | NP_001409613.1 | Q6YZI0 | Probable UDP-N-acetylglucosamine--peptide N-acetylglucosaminyltransferase SPINDLY (EC 2.4.1.255) | 2.4.1.255 | cytokinin-activated signaling pathway [GO:0009736]; gibberellic acid mediated signaling pathway [GO:0009740]; negative regulation of gibberellic acid mediated signaling pathway [GO:0009938]; regulation of reactive oxygen species metabolic process [GO:2000377] | cytoplasm [GO:0005737]; nucleus [GO:0005634] | peptide-O-fucosyltransferase activity [GO:0046922]; protein O-acetylglucosaminyltransferase activity [GO:0097363] | GO:0005634; GO:0005737; GO:0009736; GO:0009740; GO:0009938; GO:0046922; GO:0097363; GO:2000377 | Other types of O-glycan biosynthesis; Polycomb repressive complex; Insulin resistance; |
| Chr9.g32342.t1 | NP_001407865.1 | Q6K2E1 | UDP-glucose 4-epimerase 4 (OsUGE-4) (EC 5.1.3.2) (UDP-galactose 4-epimerase 4) | 5.1.3.2 | galactose metabolic process [GO:0006012]; monosaccharide metabolic process [GO:0005996] | cytosol [GO:0005829] | UDP-glucose 4-epimerase activity [GO:0003978] | GO:0003978; GO:0005829; GO:0005996; GO:0006012 | Metabolic pathways; Biosynthesis of nucleotide sugars; Galactose metabolism; Amino sugar and nucleotide sugar metabolism; |
| Chr9.g33566.t1 | NP_001390857.1 | A0A0B4U155 | UDP-glucose 4-epimerase (EC 5.1.3.-) | 5.1.3.- | galactose metabolic process [GO:0006012] | cytosol [GO:0005829] | UDP-glucose 4-epimerase activity [GO:0003978] | GO:0003978; GO:0005829; GO:0006012 | Metabolic pathways; Biosynthesis of nucleotide sugars; Galactose metabolism; Amino sugar and nucleotide sugar metabolism; |
| Chr9.g33842.t1 | BAF25866.1 | Q0IZJ9 | Arogenate dehydratase (EC 4.2.1.91) | 4.2.1.91 | L-phenylalanine biosynthetic process [GO:0009094] | chloroplast stroma [GO:0009570] | arogenate dehydratase activity [GO:0047769]; prephenate dehydratase activity [GO:0004664] | GO:0004664; GO:0009094; GO:0009570; GO:0047769 | Metabolic pathways; Biosynthesis of secondary metabolites; Biosynthesis of amino acids; Phenylalanine, tyrosine and tryptophan biosynthesis; |
| Chr10.g34368.t1 | AAN04181.1 | Q42971 | Enolase (EC 4.2.1.11) (2-phospho-D-glycerate hydro-lyase) (2-phosphoglycerate dehydratase) (OSE1) | 4.2.1.11 | glycolytic process [GO:0006096] | phosphopyruvate hydratase complex [GO:0000015] | magnesium ion binding [GO:0000287]; phosphopyruvate hydratase activity [GO:0004634] | GO:0000015; GO:0000287; GO:0004634; GO:0006096 | Metabolic pathways; Biosynthesis of secondary metabolites; Microbial metabolism in diverse environments; Carbon metabolism; Biosynthesis of amino acids; Glycolysis_Gluconeogenesis; RNA degradation; HIF-1 signaling pathway; Cytoskeleton in muscle cells; |
| Chr10.g34788.t1 | XP_015612980.1 | Q8S601 | Phosphatidate cytidylyltransferase (EC 2.7.7.41) | 2.7.7.41 | CDP-diacylglycerol biosynthetic process [GO:0016024] | endoplasmic reticulum membrane [GO:0005789] | phosphatidate cytidylyltransferase activity [GO:0004605] | GO:0004605; GO:0005789; GO:0016024 | Metabolic pathways; Biosynthesis of secondary metabolites; Glycerophospholipid metabolism; Phosphatidylinositol signaling system; |
| Chr10.g34992.t1 | AAM18739.1 | Q8S6N7 | Hexosyltransferase (EC 2.4.1.-) | 2.4.1.- | cell wall organization [GO:0071555]; pectin biosynthetic process [GO:0045489] | Golgi membrane [GO:0000139] | polygalacturonate 4-alpha-galacturonosyltransferase activity [GO:0047262] | GO:0000139; GO:0045489; GO:0047262; GO:0071555 | Metabolic pathways; Amino sugar and nucleotide sugar metabolism; |
| Chr10.g35033.t1 | NP_001410865.1 | Q8S5N2 | Inositol-3-phosphate synthase 1 (MIP synthase 1) (EC 5.5.1.4) (Myo-inositol 1-phosphate synthase) (IPS) (MI-1-P synthase) (OsINO1-2) | 5.5.1.4 | inositol biosynthetic process [GO:0006021]; phospholipid biosynthetic process [GO:0008654] | cytoplasm [GO:0005737] | inositol-3-phosphate synthase activity [GO:0004512] | GO:0004512; GO:0005737; GO:0006021; GO:0008654 | Metabolic pathways; Biosynthesis of secondary metabolites; Inositol phosphate metabolism; Streptomycin biosynthesis; |
| Chr10.g35380.t1 | NP_001390947.1 | Q7XEJ5 | Acireductone dioxygenase 4 (Acireductone dioxygenase (Fe(2+)-requiring) 4) (ARD' 4) (Fe-ARD 4) (EC 1.13.11.54) (Acireductone dioxygenase (Ni(2+)-requiring) 4) (ARD 4) (Ni-ARD 4) (EC 1.13.11.53) | 1.13.11.53; 1.13.11.54 | L-methionine salvage from methylthioadenosine [GO:0019509]; methionine metabolic process [GO:0006555] | cytoplasm [GO:0005737]; nucleus [GO:0005634] | acireductone dioxygenase (Ni2+-requiring) activity [GO:0010308]; acireductone dioxygenase [iron(II)-requiring] activity [GO:0010309]; iron ion binding [GO:0005506]; nickel cation binding [GO:0016151] | GO:0005506; GO:0005634; GO:0005737; GO:0006555; GO:0010308; GO:0010309; GO:0016151; GO:0019509 | Metabolic pathways; Cysteine and methionine metabolism; |
| Chr10.g35381.t1 | XP_015613138.1 | A0A0P0XUB0 | chlorophyllase (EC 3.1.1.14) | 3.1.1.14 | chlorophyll catabolic process [GO:0015996] | cytosol [GO:0005829] | chlorophyllase activity [GO:0047746] | GO:0005829; GO:0015996; GO:0047746 | Metabolic pathways; Biosynthesis of secondary metabolites; Porphyrin metabolism; |
| Chr10.g35732.t1 | XP_015613433.1 | Q9FW59 | 3-ketoacyl-CoA synthase (EC 2.3.1.-) | 2.3.1.- | fatty acid biosynthetic process [GO:0006633] | membrane [GO:0016020] | acyltransferase activity, transferring groups other than amino-acyl groups [GO:0016747] | GO:0006633; GO:0016020; GO:0016747 | Metabolic pathways; Biosynthesis of secondary metabolites; Fatty acid elongation; Plant-pathogen interaction; |
| Chr10.g36328.t1 | AAK31282.1 | Q9AUT8 | serine C-palmitoyltransferase (EC 2.3.1.50) | 2.3.1.50 | sphingolipid metabolic process [GO:0006665] | endoplasmic reticulum membrane [GO:0005789] | pyridoxal phosphate binding [GO:0030170]; transaminase activity [GO:0008483] | GO:0005789; GO:0006665; GO:0008483; GO:0030170 | Metabolic pathways; Biosynthesis of cofactors; Biotin metabolism; |
| Chr10.g36358.t1 | AAG60192.1 | Q9AY42 | Phospho-2-dehydro-3-deoxyheptonate aldolase (EC 2.5.1.54) | 2.5.1.54 | amino acid biosynthetic process [GO:0008652]; aromatic amino acid family biosynthetic process [GO:0009073]; chorismate biosynthetic process [GO:0009423] | chloroplast [GO:0009507] | 3-deoxy-7-phosphoheptulonate synthase activity [GO:0003849] | GO:0003849; GO:0008652; GO:0009073; GO:0009423; GO:0009507 | Metabolic pathways; Biosynthesis of secondary metabolites; Biosynthesis of amino acids; Phenylalanine, tyrosine and tryptophan biosynthesis; Quorum sensing; |
| Chr10.g36454.t1 | XP_015615005.1 | Q8W3G2 | Glycerol-3-phosphate acyltransferase, chloroplastic (GPAT) (EC 2.3.1.15) | 2.3.1.15 | CDP-diacylglycerol biosynthetic process [GO:0016024]; phosphatidylglycerol biosynthetic process [GO:0006655] | chloroplast stroma [GO:0009570] | glycerol-3-phosphate O-acyltransferase activity [GO:0004366] | GO:0004366; GO:0006655; GO:0009570; GO:0016024 | Metabolic pathways; Glycerolipid metabolism; Glycerophospholipid metabolism; |
| Chr11.g36754.t1 | NP_001410085.1 | Q2RAP0 | L-galactono-1,4-lactone dehydrogenase 1, mitochondrial (EC 1.3.2.3) | 1.3.2.3 | L-ascorbic acid biosynthetic process [GO:0019853] | mitochondrial membrane [GO:0031966] | D-arabinono-1,4-lactone oxidase activity [GO:0003885]; FAD binding [GO:0071949]; galactonolactone dehydrogenase activity [GO:0016633]; oxidoreductase activity [GO:0016491] | GO:0003885; GO:0016491; GO:0016633; GO:0019853; GO:0031966; GO:0071949 | Metabolic pathways; Biosynthesis of secondary metabolites; Biosynthesis of cofactors; Ascorbate and aldarate metabolism; |
| Chr11.g38162.t1 | NP_001391393.1 | Q2R480 | 6-phosphogluconate dehydrogenase, decarboxylating 2, chloroplastic (OsG6PGH2) (EC 1.1.1.44) | 1.1.1.44 | D-gluconate metabolic process [GO:0019521]; pentose-phosphate shunt, oxidative branch [GO:0009051]; response to abscisic acid [GO:0009737]; response to cold [GO:0009409]; response to salt stress [GO:0009651]; response to water deprivation [GO:0009414] | chloroplast [GO:0009507]; cytosol [GO:0005829] | NADP binding [GO:0050661]; phosphogluconate 2-dehydrogenase activity [GO:0008114]; phosphogluconate dehydrogenase (decarboxylating) activity [GO:0004616] | GO:0004616; GO:0005829; GO:0008114; GO:0009051; GO:0009409; GO:0009414; GO:0009507; GO:0009651; GO:0009737; GO:0019521; GO:0050661 | Metabolic pathways; Biosynthesis of secondary metabolites; Microbial metabolism in diverse environments; Carbon metabolism; Pentose phosphate pathway; Glutathione metabolism; |
| Chr11.g38976.t1 | XP_015617905.1 | Q2R0R8 | Adenylyl-sulfate kinase (EC 2.7.1.25) | 2.7.1.25 | cysteine biosynthetic process [GO:0019344]; hydrogen sulfide biosynthetic process [GO:0070814]; sulfate assimilation [GO:0000103] | cytoplasm [GO:0005737] | adenylylsulfate kinase activity [GO:0004020]; ATP binding [GO:0005524] | GO:0000103; GO:0004020; GO:0005524; GO:0005737; GO:0019344; GO:0070814 | Metabolic pathways; Microbial metabolism in diverse environments; Sulfur cycle; Sulfur metabolism; Purine metabolism; |
| Chr12.g39909.t1 | NP_001391582.1 | Q2QWK9 | Probable thiamine biosynthetic bifunctional enzyme, chloroplastic [Includes: Thiamine-phosphate synthase (TP synthase) (TPS) (EC 2.5.1.3) (Thiamine-phosphate pyrophosphorylase) (TMP pyrophosphorylase) (TMP-PPase); Hydroxymethylpyrimidine kinase (HMP kinase) (EC 2.7.1.49)] | 2.5.1.3; 2.7.1.49 | thiamine biosynthetic process [GO:0009228]; thiamine diphosphate biosynthetic process [GO:0009229] | chloroplast [GO:0009507] | ATP binding [GO:0005524]; hydroxymethylpyrimidine kinase activity [GO:0008902]; metal ion binding [GO:0046872]; phosphomethylpyrimidine kinase activity [GO:0008972]; thiamine-phosphate diphosphorylase activity [GO:0004789] | GO:0004789; GO:0005524; GO:0008902; GO:0008972; GO:0009228; GO:0009229; GO:0009507; GO:0046872 | Metabolic pathways; Biosynthesis of cofactors; Thiamine metabolism; |
| Chr12.g40179.t1 | XP_015618949.1 | Q2QV59 | 3-oxoacyl-[acyl-carrier-protein] reductase (EC 1.1.1.100) | 1.1.1.100 | fatty acid elongation [GO:0030497] | chloroplast [GO:0009507]; mitochondrion [GO:0005739] | 3-oxoacyl-[acyl-carrier-protein] reductase (NADPH) activity [GO:0004316]; NAD binding [GO:0051287]; oxidoreductase activity, acting on the CH-OH group of donors, NAD or NADP as acceptor [GO:0016616] | GO:0004316; GO:0005739; GO:0009507; GO:0016616; GO:0030497; GO:0051287 | Metabolic pathways; Biosynthesis of secondary metabolites; Fatty acid metabolism; Biosynthesis of cofactors; Fatty acid biosynthesis; Biotin metabolism; Prodigiosin biosynthesis; |
| Chr12.g40315.t1 | XP_015619694.1 | Q2QUG4 | 3-hydroxyisobutyryl-CoA hydrolase (HIB-CoA hydrolase) (HIBYL-CoA-H) (EC 3.1.2.4) (3-hydroxyisobutyryl-coenzyme A hydrolase) | 3.1.2.4 | L-valine catabolic process [GO:0006574] | membrane [GO:0016020] | 3-hydroxyisobutyryl-CoA hydrolase activity [GO:0003860] | GO:0003860; GO:0006574; GO:0016020 | Metabolic pathways; Carbon metabolism; Propanoate metabolism; Valine, leucine and isoleucine degradation; beta-Alanine metabolism; |
| Chr12.g40838.t1 | XP_015618537.1 | Q2QS20 | Sulfite oxidase (EC 1.8.3.1) | 1.8.3.1 | sulfur compound metabolic process [GO:0006790] | mitochondrion [GO:0005739] | heme binding [GO:0020037]; molybdenum ion binding [GO:0030151]; molybdopterin cofactor binding [GO:0043546]; sulfite oxidase activity [GO:0008482] | GO:0005739; GO:0006790; GO:0008482; GO:0020037; GO:0030151; GO:0043546 | Metabolic pathways; Microbial metabolism in diverse environments; Sulfur metabolism; |
| Chr12.g40845.t1 | NP_001410424.1 | A0A0B4U2C3 | UDP-glucose 6-dehydrogenase (EC 1.1.1.22) | 1.1.1.22 | glycosaminoglycan biosynthetic process [GO:0006024]; UDP-glucuronate biosynthetic process [GO:0006065] | nucleus [GO:0005634] | NAD binding [GO:0051287]; UDP-glucose 6-dehydrogenase activity [GO:0003979] | GO:0003979; GO:0005634; GO:0006024; GO:0006065; GO:0051287 | Metabolic pathways; Biosynthesis of nucleotide sugars; Biosynthesis of cofactors; Pentose and glucuronate interconversions; Ascorbate and aldarate metabolism; Amino sugar and nucleotide sugar metabolism; Biosynthesis of various nucleotide sugars; |
| Chr12.g41205.t1 | XP_015619367.1 | Q2QQ95 | phosphoserine phosphatase (EC 3.1.3.3) (O-phosphoserine phosphohydrolase) | 3.1.3.3 | L-serine biosynthetic process [GO:0006564] | chloroplast [GO:0009507]; cytoplasm [GO:0005737] | L-phosphoserine phosphatase activity [GO:0036424]; magnesium ion binding [GO:0000287] | GO:0000287; GO:0005737; GO:0006564; GO:0009507; GO:0036424 | Metabolic pathways; Biosynthesis of secondary metabolites; Microbial metabolism in diverse environments; Carbon metabolism; Biosynthesis of amino acids; Glycine, serine and threonine metabolism; |
| Chr12.g41325.t1 | XP_015620761.1 | Q2QPR0 | Phenylalanine ammonia-lyase (EC 4.3.1.24) | 4.3.1.24 | cinnamic acid biosynthetic process [GO:0009800]; L-phenylalanine catabolic process [GO:0006559] | cytoplasm [GO:0005737] | phenylalanine ammonia-lyase activity [GO:0045548] | GO:0005737; GO:0006559; GO:0009800; GO:0045548 | Metabolic pathways; Biosynthesis of secondary metabolites; Phenylalanine metabolism; Phenylpropanoid biosynthesis; |
| Chr12.g41542.t1 | XP_015620637.1 | Q9FSE5 | Lipoxygenase (EC 1.13.11.-) | 1.13.11.- | fatty acid biosynthetic process [GO:0006633]; lipid oxidation [GO:0034440]; oxylipin biosynthetic process [GO:0031408]; positive regulation of gene expression [GO:0010628] | chloroplast [GO:0009507] | linoleate 13S-lipoxygenase activity [GO:0016165]; metal ion binding [GO:0046872] | GO:0006633; GO:0009507; GO:0010628; GO:0016165; GO:0031408; GO:0034440; GO:0046872 | Metabolic pathways; Biosynthesis of secondary metabolites; Linoleic acid metabolism; alpha-Linolenic acid metabolism; |
| Chr12.g41726.t1 | XP_015620778.1 | Q2QMV8 | adenine phosphoribosyltransferase (EC 2.4.2.7) | 2.4.2.7 | adenine salvage [GO:0006168]; AMP salvage [GO:0044209]; purine ribonucleoside salvage [GO:0006166] | cytosol [GO:0005829] | adenine phosphoribosyltransferase activity [GO:0003999] | GO:0003999; GO:0005829; GO:0006166; GO:0006168; GO:0044209 | Metabolic pathways; Nucleotide metabolism; Purine metabolism; |
| Chr12.g41742.t1 | ABA99160.1 | Q2QMT1 | adenine phosphoribosyltransferase (EC 2.4.2.7) | 2.4.2.7 | adenine salvage [GO:0006168]; AMP salvage [GO:0044209]; purine ribonucleoside salvage [GO:0006166] | cytosol [GO:0005829] | adenine phosphoribosyltransferase activity [GO:0003999] | GO:0003999; GO:0005829; GO:0006166; GO:0006168; GO:0044209 | Metabolic pathways; Nucleotide metabolism; Purine metabolism; |
| Chr12.g41775.t1 | NP_001410539.1 | Q2QMN7 | Ureidoglycolate hydrolase (OsUAH) (EC 3.5.1.116) | 3.5.1.116 | allantoin catabolic process [GO:0000256]; purine nucleobase catabolic process [GO:0006145]; ureide catabolic process [GO:0010136] | endoplasmic reticulum [GO:0005783] | hydrolase activity, acting on carbon-nitrogen (but not peptide) bonds, in linear amidines [GO:0016813]; manganese ion binding [GO:0030145]; ureidoglycolate hydrolase activity [GO:0004848] | GO:0000256; GO:0004848; GO:0005783; GO:0006145; GO:0010136; GO:0016813; GO:0030145 | Metabolic pathways; Microbial metabolism in diverse environments; Purine metabolism; |
| Chr12.g41959.t1 | NP_001391690.1 | Q2QLX5 | Cysteine synthase (EC 2.5.1.47) | 2.5.1.47 | cysteine biosynthetic process from serine [GO:0006535] | cytoplasm [GO:0005737] | cysteine synthase activity [GO:0004124] | GO:0004124; GO:0005737; GO:0006535 | Metabolic pathways; Biosynthesis of secondary metabolites; Microbial metabolism in diverse environments; Carbon metabolism; Biosynthesis of amino acids; Sulfur cycle; Sulfur metabolism; Cysteine and methionine metabolism; |

**Table 2: Key Genes with Multiple Biological Functions**

| Genes | Refseq ID | Entry | Multifunction | Gene Ontology IDs | EC number |
| --- | --- | --- | --- | --- | --- |
| Chr1.g6.t1 | NP_001384927.1 | A0A0E0FEY0 | Lipid metabolism; Sphingolipid metabolism | GO:0005789; GO:0008117; GO:0019752; GO:0030149; GO:0030170 | 5.3.3.5 |
| Chr1.g26.t1 | NP_001384925.1 | Q9FTZ2 | Steroid biosynthesis; Sterol biosynthesis | GO:0000247; GO:0004769; GO:0005783; GO:0005789; GO:0016126; GO:0047750 | 5.3.3.5 |
| Chr1.g32.t1 | XP_015620624.1 | A0A0E0J8C8 | Amino-acid biosynthesis; L-arginine biosynthesis | GO:0000050; GO:0000053; GO:0004055; GO:0005524; GO:0005737; GO:0006526 | 6.3.4.5 |
| Chr1.g50.t1 | XP_015618780.1 | A0A0D3EIP9 | Protein modification; Protein ubiquitination | GO:0005634; GO:0005737; GO:0008270; GO:0009867 | - |
| Chr1.g51.t1 | NP_001359128.1 | A0A0E0FF26 | Isoprenoid biosynthesis; Isopentenyl diphosphate biosynthesis | GO:0009507; GO:0030145; GO:0030604; GO:0051484; GO:0070402 | 1.1.1.267 |
| Chr1.g182.t1 | XP_015640867.1 | Q9ART8 | Protein modification; Protein ubiquitination | GO:0008270; GO:0016740 | 2.3.2.27 |
| Chr1.g359.t1 | NP_001393157.1 | P48494 | Carbohydrate biosynthesis; Gluconeogenesis | GO:0004807; GO:0005829; GO:0006094; GO:0006096; GO:0019563; GO:0046166 | 5.3.1.1 |
| Chr1.g524.t1 | NP_001396345.1 | A0A0D3EK26 | Plant hormone metabolism; Auxin biosynthesis | GO:0006520; GO:0008483; GO:0009851; GO:0016020; GO:0016846 | 2.6.1.27 |
| Chr1.g691.t1 | XP_015621540.1 | A0A0D3EKJ9 | Carbohydrate degradation; Glycolysis | GO:0003872; GO:0005524; GO:0005829; GO:0006002; GO:0046872 | 2.7.1.11 |
| Chr1.g743.t1 | XP_052152324.1 | I1NL64 | Purine metabolism; IMP biosynthesis | GO:0004638; GO:0005524; GO:0006189; GO:0009507; GO:0046872 | 4.1.1.21 |
| Chr1.g934.t1 | XP_015645815.1 | I1NLG6 | Cofactor biosynthesis; Pantothenate biosynthesis | GO:0000287; GO:0003864; GO:0005739; GO:0015940 | 2.1.2.11 |
| Chr1.g986.t1 | NP_001393037.1 | A0A0E0FJ38 | Amino-acid biosynthesis; Histidine biosynthesis | GO:0000105; GO:0004399; GO:0005829; GO:0009570; GO:0046872; GO:0051287 | - |
| Chr1.g1001.t1 | XP_015633470.1 | C7IX66 | Glycan metabolism; Pectin degradation | GO:0004857; GO:0016020; GO:0030599; GO:0042545; GO:0045490 | 3.1.1.11 |
| Chr1.g1005.t1 | XP_015633666.1 | Q5NAX7 | Protein modification; Ubiquitin pathway | GO:0005737; GO:0006511; GO:0008270; GO:0016567; GO:0061630 | 2.3.2.27 |
| Chr1.g1226.t1 | XP_015611288.1 | A0A0E0FJS0 | Cofactor biosynthesis; Tocopherol biosynthesis | GO:0009535; GO:0015979; GO:0015995; GO:0045550; GO:0071949; GO:0102067 | 1.3.1.83 |
| Chr1.g1270.t1 | XP_015610620.1 | B9EV56 | Protein modification; Protein neddylation | GO:0005524; GO:0005634; GO:0005737; GO:0019781; GO:0045116 | 6.2.1.64 |
| Chr1.g1311.t1 | XP_015611206.1 | A0A0E0MUL5 | Amino-acid biosynthesis; Histidine pathway | GO:0000105; GO:0004635; GO:0004636; GO:0005524 | - |
| Chr1.g1332.t1 | XP_015622109.1 | Q9SDJ2 | Porphyrin metabolism; Tetrapyrrole biosynthesis | GO:0003729; GO:0005506; GO:0009535; GO:0009658; GO:0009706; GO:0015979; GO:0015995; GO:0036068; GO:0048529; GO:1901401 | 1.14.13.81 |
| Chr1.g1394.t1 | BAD81569.1 | Q9AR38 | Porphyrin metabolism; Chlorophyll biosynthesis | GO:0004729; GO:0006782; GO:0009507; GO:0015995; GO:0050660 | 1.3.3.4 |
| Chr1.g1435.t1 | NP_001396337.1 | A0A0E0FKG0 | Amino-acid biosynthesis; S-adenosyl-L-methionine pathway | GO:0004478; GO:0005524; GO:0005737; GO:0006556; GO:0006730; GO:0046872 | 2.5.1.6 |

**Key Genes Directly Involved in Starch Metabolism**

| **Gene ID** | **RefSeq ID** | **Protein Name** | **EC Number** | **GO Biological Process** | **KEGG Pathway** | **Entry** | **Gene ontology IDs** |
| --- | --- | --- | --- | --- | --- | --- | --- |
| Chr1.g1022.t1 | XP_015613032.1 | Beta-amylase | EC 3.2.1.2 | Starch catabolic process | Starch and sucrose metabolism | Q5NB81 | GO:0005737; GO:0005975; GO:0030246; GO:0047938 |
| Chr1.g4606.t1 | XP_052137320.1 | Glucan endo-1,3-β-D-glucosidase | EC 3.2.1.39 | Carbohydrate metabolic process | Starch and sucrose metabolism | A0A0D3EWK5 | GO:0004553; GO:0005975 |
| Chr1.g5152.t1 | XP_015627053.1 | Beta-1,3-glucanase | - | Polysaccharide catabolic process | Carbohydrate metabolism | A0A0D3EYI3 | GO:0005975; GO:0042973 |
| Chr1.g5153.t1 | XP_015621782.1 | Beta-1,3-glucanase | - | Glucan metabolic process | Carbohydrate metabolism | A0A0E0FYL8 | GO:0005975; GO:0042973 |
| Chr1.g5157.t1 | BAD87992.1 | Beta-1,3-glucanase | - | Glucan catabolic process | Carbohydrate metabolism | Q5JKL6 | GO:0005975; GO:0042973 |
| Chr2.g6131.t1 | XP_015624669.1 | Glucan endo-1,3-β-D-glucosidase | EC 3.2.1.39 | Carbohydrate metabolic process | Starch and sucrose metabolism | Q6Z7A0 | GO:0004553; GO:0005886; GO:0005975 |
| Chr2.g7618.t1 | XP_015627503.1 | 1,4-alpha-glucan branching enzyme | EC 2.4.1.18 | Starch biosynthetic process | Starch and sucrose metabolism | Q40663 | GO:0003844; GO:0004553; GO:0005978; GO:0005983; GO:0009501; GO:0019252; GO:0043169 |
| Chr2.g7651.t1 | XP_015626214.1 | Glucan endo-1,3-β-D-glucosidase | EC 3.2.1.39 | Glucan catabolic process | Carbohydrate metabolism | Q6ESE5 | GO:0004553; GO:0005886; GO:0005975 |
| Chr2.g7651.t1 | XP_015626214.1 | Glucan endo-1,3-β-D-glucosidase | EC 3.2.1.39 | Polysaccharide metabolic process | Carbohydrate metabolism | Q6ESE5 | GO:0004553; GO:0005886; GO:0005975 |
| Chr2.g9255.t1 | NP_001403849.1 | Alpha-amylase | EC 3.2.1.1 | Starch catabolic process | Starch and sucrose metabolism | Q0DXA4 | GO:0004556; GO:0005509; GO:0005983; GO:0005987 |
| Chr2.g9306.t1 | XP_015625926.1 | Glucan endo-1,3-β-D-glucosidase | EC 3.2.1.39 | Carbohydrate metabolic process | Carbohydrate metabolism | A0A0E0GDE3 | GO:0005886; GO:0005975; GO:0006952; GO:0009506; GO:0042973; GO:0098552 |
| Chr3.g10555.t1 | XP_015628658.1 | Glucan endo-1,3-β-D-glucosidase | EC 3.2.1.39 | Polysaccharide metabolic process | Carbohydrate metabolism | A0A0D3FFK7 | GO:0005886; GO:0005975; GO:0006952; GO:0009506; GO:0042973; GO:0098552 |
| Chr3.g12756.t1 | XP_015631362.1 | Glucan endo-1,3-β-D-glucosidase | EC 3.2.1.39 | Glucan metabolic process | Carbohydrate metabolism | A0A0D3FFK7 | GO:0005886; GO:0005975; GO:0006952; GO:0009506; GO:0042973; GO:0098552 |
| Chr3.g13684.t1 | XP_015630594.1 | Glucan endo-1,3-β-D-glucosidase | EC 3.2.1.39 | Carbohydrate metabolic process | Carbohydrate metabolism | A0A0E0GUT1 | GO:0005886; GO:0005975; GO:0006952; GO:0042973; GO:0098552 |
| Chr5.g20890.t1 | XP_015639411.1 | Glucan endo-1,3-β-glucosidase | – | Polysaccharide metabolic process | Carbohydrate metabolism | I1PWW8 | GO:0005975; GO:0042973 |
| Chr5.g21182.t1 | XP_015638033.1 | Glucan endo-1,3-β-D-glucosidase | EC 3.2.1.39 | Glucan catabolic process | Carbohydrate metabolism | A0A0E0PQC6 | GO:0005886; GO:0005975; GO:0006952; GO:0042973; GO:0098552 |
| Chr6.g23371.t1 | XP_015644349.1 | 1,4-alpha-glucan branching enzyme | EC 2.4.1.18 | Starch biosynthetic process | Starch and sucrose metabolism | Q5VN31 | GO:0003844; GO:0004553; GO:0005737; GO:0005975; GO:0005978; GO:0005983; GO:0009501; GO:0019252; GO:0043169 |
| Chr6.g23808.t1 | BAD54223.1 | Glucan endo-1,3-β-D-glucosidase | EC 3.2.1.39 | Carbohydrate metabolic process | Carbohydrate metabolism | Q5Z791 | GO:0004553; GO:0005975 |
| Chr6.g24141.t1 | XP_015642488.1 | Glucan endo-1,3-β-D-glucosidase | EC 3.2.1.39 | Polysaccharide catabolic process | Carbohydrate metabolism | A0A0E0GHT8 | GO:0005886; GO:0005975; GO:0006952; GO:0042973; GO:0098552 |
| Chr6.g24243.t1 | XP_015644481.1 | Glucan endo-1,3-β-D-glucosidase | EC 3.2.1.39 | Glucan metabolic process | Carbohydrate metabolism | Q0DB31 | GO:0004553; GO:0005975 |
| Chr7.g25507.t1 | NP_001408924.1 | Glucan endo-1,3-β-D-glucosidase | EC 3.2.1.39 | Carbohydrate metabolic process | Carbohydrate metabolism | Q69LE4 | GO:0005886; GO:0005975; GO:0006952; GO:0042973; GO:0098552 |
| Chr7.g25980.t1 | XP_015646239.1 | Glucan endo-1,3-β-D-glucosidase | EC 3.2.1.39 | Polysaccharide metabolic process | Carbohydrate metabolism | Q8GTK3 | GO:0004553; GO:0005886; GO:0005975 |
| Chr7.g27641.t1 | BAC83070.1 | Glucan endo-1,3-β-D-glucosidase | EC 3.2.1.39 | Glucan catabolic process | Carbohydrate metabolism | Q6ZL97 | GO:0005975; GO:0006952; GO:0042973 |
| Chr8.g30041.t1 | NP_001390460.1 | ADP-glucose pyrophosphorylase | EC 2.7.7.27 | Starch biosynthetic process | Starch and sucrose metabolism | P15280 | GO:0005524; GO:0005829; GO:0005978; GO:0005982; GO:0008878; GO:0009501; GO:0009507; GO:0019252 |
| Chr8.g30792.t1 | NP_001390565.1 | Alpha-amylase | EC 3.2.1.1 | Starch catabolic process | Starch and sucrose metabolism | A0A0D3H1A8 | GO:0004556; GO:0005509; GO:0005983; GO:0005987 |
| Chr9.g31948.t1 | XP_015651504.1 | Glucan endo-1,3-β-D-glucosidase | EC 3.2.1.39 | Carbohydrate metabolic process | Carbohydrate metabolism | A0A0E0IH87 | GO:0005886; GO:0005975; GO:0006952; GO:0042973; GO:0098552 |
| Chr9.g33422.t1 | XP_015610655.1 | Glucan endo-1,3-β-D-glucosidase | EC 3.2.1.39 | Polysaccharide metabolic process | Carbohydrate metabolism | A0A0E0QTD0 | GO:0005886; GO:0005975; GO:0006952; GO:0009506; GO:0042973; GO:0098552 |
| Chr10.g34287.t1 | AAN04212.1 | Glucan endo-1,3-β-D-glucosidase | EC 3.2.1.39 | Glucan catabolic process | Carbohydrate metabolism | Q8LMT5 | GO:0004553; GO:0005975 |
| Chr11.g38664.t1 | XP_015616306.1 | Glucan endo-1,3-β-D-glucosidase | EC 3.2.1.39 | Carbohydrate metabolic process | Carbohydrate metabolism | Q2R256 | GO:0005886; GO:0005975; GO:0006952; GO:0042973; GO:0098552 |

**Table : Genes for aroma**

| **Gene ID** | **RefSeq ID** | **Protein Name** | **Entry** | **EC Number** | **GO Biological Process** | **GO Reference ID** | **KEGG Pathway** |
| --- | --- | --- | --- | --- | --- | --- | --- |
| Chr1.g2811.t1 | XP_015618943.1 | Aldehyde dehydrogenase 1 | Q9LRE9 | - | - | GO:0004029 | Biosynthesis of secondary metabolites; Phenylpropanoid biosynthesis; |
| Chr1.g2812.t1 | XP_052141551.1 | Aldehyde dehydrogenase 1 | A0A0D3EQR1 | - | - | GO:0004030 | Biosynthesis of secondary metabolites; Phenylpropanoid biosynthesis; |
| Chr2.g8444.t1 | XP_052145385.1 | Aldehyde dehydrogenase | I1P2L4 | - | aldehyde metabolic process [GO:0006081]; response to abscisic acid | GO:0004029; GO:0005737; GO:0006081; GO:0009737 | - |
| Chr4.g17070.t1 | XP_015635925.1 | Aldehyde dehydrogenase | Q01IP6 | - | aldehyde metabolic process [GO:0006081]; response to abscisic acid [GO:0009737] | GO:0004029; GO:0005737; GO:0006081; GO:0009737 | Metabolic pathways; Biosynthesis of secondary metabolites; Microbial metabolism in diverse environments; Biosynthesis of cofactors; Glycolysis_Gluconeogenesis; Ascorbate and aldarate metabolism; Pyruvate metabolism; Fatty acid degradation; Glycerolipid metabolism; Valine, leucine and isoleucine degradation; Lysine degradation; Arginine and proline metabolism; Histidine metabolism; Tryptophan metabolism; beta-Alanine metabolism; Pantothenate and CoA biosynthesis; Insect hormone biosynthesis; Limonene degradation; Chloroalkane and chloroalkene degradation; |
| Chr9.g33035.t1 | XP_015611835.1 | aldehyde dehydrogenase (NAD(+)) (EC 1.2.1.3) | Q9FPK6 | 1.2.1.3 | - | GO:0004029 | Metabolic pathways; Biosynthesis of secondary metabolites; Microbial metabolism in diverse environments; Glycolysis_Gluconeogenesis; Ascorbate and aldarate metabolism; Pyruvate metabolism; Fatty acid degradation; Glycerolipid metabolism; Glycine, serine and threonine metabolism; Valine, leucine and isoleucine degradation; Lysine degradation; Arginine and proline metabolism; Histidine metabolism; Tryptophan metabolism; beta-Alanine metabolism; One carbon pool by folate; |
| Chr11.g37026.t1 | XP_015617047.1 | Aldehyde dehydrogenase | Q53NG8 | - | aldehyde metabolic process [GO:0006081]; response to abscisic acid [GO:0009737] | GO:0004029; GO:0005737; GO:0006081; GO:0009737; GO:0016020 | Metabolic pathways; Biosynthesis of secondary metabolites; Microbial metabolism in diverse environments; Biosynthesis of cofactors; Glycolysis_Gluconeogenesis; Ascorbate and aldarate metabolism; Pyruvate metabolism; Fatty acid degradation; Glycerolipid metabolism; Valine, leucine and isoleucine degradation; Lysine degradation; Arginine and proline metabolism; Histidine metabolism; Tryptophan metabolism; beta-Alanine metabolism; Pantothenate and CoA biosynthesis; Insect hormone biosynthesis; Limonene degradation; Chloroalkane and chloroalkene degradation; |
| Chr4.g16537.t1  BADH2 | NP_001406345.1 | betaine-aldehyde dehydrogenase (EC 1.2.1.8) | A0A8F9RXP4 | 1.2.1.8 | cellular response to anoxia [GO:0071454] | GO:0005777; GO:0008802; GO:0071454 | - |
| Chr1.g4517.t1 P5CS | XP_015622203.1 | Delta-1-pyrroline-5-carboxylate synthase 2 (OsP5CS2) [Includes: Glutamate 5-kinase (GK) (EC 2.7.2.11) (Gamma-glutamyl kinase); Gamma-glutamyl phosphate reductase (GPR) (EC 1.2.1.41) (Glutamate-5-semialdehyde dehydrogenase) (Glutamyl-gamma-semialdehyde dehydrogenase)] | Q941T1 | 1.2.1.41; 2.7.2.11 | L-proline biosynthetic process [GO:0055129]; proline biosynthetic process [GO:0006561] | GO:0004349; GO:0004350; GO:0005524; GO:0005737; GO:0006561; GO:0055129 | Metabolic pathways; Biosynthesis of secondary metabolites; Biosynthesis of amino acids; Arginine and proline metabolism; |
| Chr5.g20618.t1  P5CS | NP_001389494.1 | Delta-1-pyrroline-5-carboxylate synthase 1 (OsP5CS1) [Includes: Glutamate 5-kinase (GK) (EC 2.7.2.11) (Gamma-glutamyl kinase); Gamma-glutamyl phosphate reductase (GPR) (EC 1.2.1.41) (Glutamate-5-semialdehyde dehydrogenase) (Glutamyl-gamma-semialdehyde dehydrogenase)] | O04226 | 1.2.1.41; 2.7.2.11 | L-proline biosynthetic process [GO:0055129]; proline biosynthetic process [GO:0006561] | GO:0004349; GO:0004350; GO:0005524; GO:0005737; GO:0006561; GO:0055129 | Metabolic pathways; Biosynthesis of secondary metabolites; Biosynthesis of amino acids; Arginine and proline metabolism; |
| Chr3.g13115.t1 phospholipase  A2 | NP_001405447.1 | arachidonate secretion [GO:0050482]; lipid catabolic process [GO:0016042]; phospholipid metabolic process [GO:0006644] | I1PEV8 | 3.1.1.4 | arachidonate secretion [GO:0050482]; lipid catabolic process [GO:0016042]; phospholipid metabolic process [GO:0006644] | GO:0004623; GO:0005576; GO:0005794; GO:0006644; GO:0016042; GO:0050482 | Metabolic pathways; Biosynthesis of secondary metabolites; Glycerophospholipid metabolism; Ether lipid metabolism; Arachidonic acid metabolism; Linoleic acid metabolism; alpha-Linolenic acid metabolism; Ras signaling pathway; Vascular smooth muscle contraction; Fat digestion and absorption; |
| Chr2.g9593.t1  ALCOHOL DEHYDROGENASE | XP_052145580.1 | Alcohol dehydrogenase class-3 (EC 1.1.1.1) (Alcohol dehydrogenase class-III) (Glutathione-dependent formaldehyde dehydrogenase) (FALDH) (FDH) (GSH-FDH) (EC 1.1.1.-) (S-(hydroxymethyl)glutathione dehydrogenase) (EC 1.1.1.284) | A2XAZ3 | 1.1.1.-; 1.1.1.1; 1.1.1.284 | formaldehyde catabolic process [GO:0046294]; heat acclimation [GO:0010286]; seed development [GO:0048316] | GO:0004022; GO:0005829; GO:0008270; GO:0010286; GO:0046294; GO:0048316; GO:0080007; GO:0106321; GO:0106322 | Metabolic pathways; Biosynthesis of secondary metabolites; Microbial metabolism in diverse environments; Carbon metabolism; Degradation of aromatic compounds; Glycolysis_Gluconeogenesis; Pyruvate metabolism; Fatty acid degradation; Tyrosine metabolism; Retinol metabolism; Chloroalkane and chloroalkene degradation; Metabolism of xenobiotics by cytochrome P450; Drug metabolism - cytochrome P450; |
| Chr11.g37153.t1 | NP_001391382.1 | Alcohol dehydrogenase 1 (EC 1.1.1.1) | Q75ZX3 | 1.1.1.1 | formaldehyde catabolic process [GO:0046294] | GO:0005829; GO:0008270; GO:0046294; GO:0051903 | Metabolic pathways; Biosynthesis of secondary metabolites; Microbial metabolism in diverse environments; Glycolysis_Gluconeogenesis; Pyruvate metabolism; Fatty acid degradation; alpha-Linolenic acid metabolism; Tyrosine metabolism; |
| Chr11.g37154.t1 | NP_001391383.1 | alcohol dehydrogenase (EC 1.1.1.1) | A0A0R7VIL1 | 1.1.1.1 | - | GO:0005737; GO:0008270; GO:0016491 | Metabolic pathways; Biosynthesis of secondary metabolites; Microbial metabolism in diverse environments; Glycolysis_Gluconeogenesis; Pyruvate metabolism; Fatty acid degradation; alpha-Linolenic acid metabolism; Tyrosine metabolism; |
| Chr11.g37155.t1 | XP_015616852.1 | alcohol dehydrogenase (EC 1.1.1.1) | A0A0E0HCF3 | 1.1.1.1 | formaldehyde catabolic process [GO:0046294] | GO:0005829; GO:0008270; GO:0046294; GO:0051903 | Metabolic pathways; Biosynthesis of secondary metabolites; Microbial metabolism in diverse environments; Glycolysis_Gluconeogenesis; Pyruvate metabolism; Fatty acid degradation; alpha-Linolenic acid metabolism; Tyrosine metabolism; |
| Chr3.g13061.t1 | XP_052150013.1 | Lipoxygenase (EC 1.13.11.-) | I1PEN1 | 1.13.11.- | fatty acid biosynthetic process [GO:0006633]; lipid oxidation [GO:0034440]; oxylipin biosynthetic process [GO:0031408]; response to other organism [GO:0051707]; response to wounding [GO:0009611] | GO:0006633; GO:0009611; GO:0031408; GO:0034440; GO:0046872; GO:0051707; GO:1990136 | Linoleic acid metabolism; |
| Chr8.g31025.t1 | NP_001390579.1 | D1G597 | Lipoxygenase | 1.13.11 | fatty acid biosynthetic process [GO:0006633]; lipid oxidation [GO:0034440]; oxylipin biosynthetic process [GO:0031408]; response to other organism [GO:0051707]; response to wounding [GO:0009611] | GO:0006633; GO:0009507; GO:0009611; GO:0016702; GO:0031408; GO:0034440; GO:0046872; GO:0051707 | Metabolic pathways; Biosynthesis of secondary metabolites; Linoleic acid metabolism; alpha-Linolenic acid metabolism; |
|  |  |  |  |  |  |  |  |

**Table: secondary metabolite**

| **Gene ID** | **RefSeq ID** | **Protein Name** | **Entry** | **EC Number** | **GO Biological Process** | **GO Reference ID** | **KEGG Pathway** |
| --- | --- | --- | --- | --- | --- | --- | --- |
| Chr1.g3103.t1 | AAB58474.1 | Flavanone 4-reductase (EC 1.1.1.219) (EC 1.1.1.234) | P94105 | 1.1.1.219; 1.1.1.234 | flavonoid biosynthetic process [GO:0009813] | [GO:0009813] | - |
| Chr1.g3246.t1 | XP_015621380.1 | Long-chain-fatty-acid--CoA ligase (EC 6.2.1.3) | A0A0E0FQF6 | 6.2.1.3 | phenylpropanoid metabolic process [GO:0009698] | GO:0004467; GO:0005524; GO:0005783; GO:0009698; GO:0016020; GO:0016207; GO:0106290 | Metabolic pathways; Fatty acid metabolism; Fatty acid biosynthesis; Fatty acid degradation; Peroxisome; Ferroptosis; Quorum sensing; Adipocytokine signaling pathway; PPAR signaling pathway; Thermogenesis; |
| Chr1.g3415.t1 | XP_015621736.1 | Long-chain-fatty-acid--CoA ligase (EC 6.2.1.3) | B9EYM0 | 6.2.1.3 | phenylpropanoid metabolic process [GO:0009698] | GO:0004467; GO:0005524; GO:0009698; GO:0016207; GO:0106290 | Metabolic pathways; Fatty acid metabolism; Fatty acid biosynthesis; Fatty acid degradation; Peroxisome; Ferroptosis; Quorum sensing; Adipocytokine signaling pathway; PPAR signaling pathway; Thermogenesis; |
| Chr1.g4199.t1 | NP_001388670.1 | 4-(cytidine 5'-diphospho)-2-C-methyl-D-erythritol kinase (EC 2.7.1.148) (4-(cytidine-5'-diphospho)-2-C-methyl-D-erythritol kinase) | A0A0E0FUI8 | 2.7.1.148 | terpenoid biosynthetic process [GO:0016114] | GO:0005524; GO:0009507; GO:0016114; GO:0050515 | Metabolic pathways; Biosynthesis of secondary metabolites; Terpenoid backbone biosynthesis; |
| Chr2.g5884.t1 | NP_001396268.1 | 4-hydroxyphenylpyruvate dioxygenase | A0A0E0G1W2 | - | carotenoid biosynthetic process [GO:0016117]; L-phenylalanine catabolic process [GO:0006559]; plastoquinone biosynthetic process [GO:0010236]; tyrosine catabolic process [GO:0006572]; vitamin E biosynthetic process [GO:0010189] | GO:0003868; GO:0005506; GO:0005829; GO:0006559; GO:0006572; GO:0010189; GO:0010236; GO:0016117; GO:0042802 | Metabolic pathways; Biosynthesis of cofactors; Tyrosine metabolism; Phenylalanine metabolism; Ubiquinone and other terpenoid-quinone biosynthesis; |
| Chr2.g9268.t1 | XP_015624815.1 | anthocyanidin synthase (EC 1.14.20.4) | A0A0E0GCD4 | 1.14.20.4 | flavonoid biosynthetic process [GO:0009813]; pigment biosynthetic process [GO:0046148] | GO:0009813; GO:0031418; GO:0046148; GO:0046872; GO:0051213 | Metabolic pathways; Biosynthesis of secondary metabolites; Flavonoid biosynthesis; |
| Chr3.g9914.t1 | BAF10729.1 | beta-carotene 3-hydroxylase (EC 1.14.15.24) | Q0DVK9 | 1.14.15.24 | carotenoid biosynthetic process [GO:0016117] | GO:0005506; GO:0016117; GO:0016491; GO:0031969; GO:0071771 | Metabolic pathways; Biosynthesis of secondary metabolites; Carotenoid biosynthesis; |
| Chr3.g9967.t1 | NP_001404106.1 | 4-coumarate--CoA ligase (EC 6.2.1.12) | I1P788 | 6.2.1.12 | phenylpropanoid metabolic process [GO:0009698] | GO:0005524; GO:0005777; GO:0009698; GO:0016020; GO:0016207; GO:0106290 | Metabolic pathways; Biosynthesis of secondary metabolites; alpha-Linolenic acid metabolism; |
| Chr3.g9977.t1 | XP_015630171.1 | 4-coumarate--CoA ligase (EC 6.2.1.12) | Q10S60 | 6.2.1.12 | phenylpropanoid metabolic process [GO:0009698] | GO:0009698; GO:0016207; GO:0106290 | - |
| Chr3.g10111.t1 | NP_001404146.1 | 4-coumarate--CoA ligase-like 1 (EC 6.2.1.12) | Q0DV32 | 6.2.1.12 | phenylpropanoid metabolic process [GO:0009698] | GO:0005524; GO:0009698; GO:0016207; GO:0016405; GO:0106290 | Metabolic pathways; Biosynthesis of secondary metabolites; Ubiquinone and other terpenoid-quinone biosynthesis; Phenylpropanoid biosynthesis; |
| Chr3.g11118.t1 | XP_052150241.1 | 4-coumarate--CoA ligase (EC 6.2.1.12) | A0A0E0GLA8 | 6.2.1.12 | phenylpropanoid metabolic process [GO:0009698] | GO:0009698; GO:0016207; GO:0106290 | Metabolic pathways; Biosynthesis of secondary metabolites; Microbial metabolism in diverse environments; Carbon metabolism; Glycolysis_Gluconeogenesis; Pyruvate metabolism; Glyoxylate and dicarboxylate metabolism; Butanoate metabolism; |
| Chr3.g11119.t1 | XP_015631446.1 | 4-coumarate--CoA ligase (EC 6.2.1.12) | I1PAK2 | 6.2.1.12 | phenylpropanoid metabolic process [GO:0009698] | GO:0009698; GO:0016207; GO:0106290 | Metabolic pathways; Biosynthesis of secondary metabolites; Microbial metabolism in diverse environments; Carbon metabolism; Glycolysis_Gluconeogenesis; Pyruvate metabolism; Glyoxylate and dicarboxylate metabolism; Butanoate metabolism; |
| Chr3.g13790.t1 | XP_015627840.1 | 4-coumarate--CoA ligase (EC 6.2.1.12) | Q10BT3 | 6.2.1.12 | phenylpropanoid metabolic process [GO:0009698] | GO:0009698; GO:0016207; GO:0106290 | - |
| Chr3.g13903.t1 | NP_001405881.1 | Chalcone-flavonone isomerase family protein | A0A0D9ZF13 | - | flavonoid biosynthetic process [GO:0009813] | GO:0009813; GO:0045430 | Metabolic pathways; Biosynthesis of secondary metabolites; Flavonoid biosynthesis; |
| Chr4.g18003.t1 | XP_052154092.1 | 4-coumarate--CoA ligase (EC 6.2.1.12) | I1PQV2 | 6.2.1.12 | phenylpropanoid metabolic process [GO:0009698] | GO:0009698; GO:0016207; GO:0106290 | - |
| Chr5.g18913.t1 | XP_015638939.1 | Os05g0212500 protein | Q6I5D0 | - | flavonoid biosynthetic process [GO:0009813] | GO:0009813; GO:0016747 | Biosynthesis of secondary metabolites; Stilbenoid, diarylheptanoid and gingerol biosynthesis; |
| Chr5.g18915.t1 | XP_015638862.1 | Chalcone synthase | Q6I5C6 | - | flavonoid biosynthetic process [GO:0009813] | GO:0009813; GO:0016747 | Biosynthesis of secondary metabolites; Stilbenoid, diarylheptanoid and gingerol biosynthesis; |
| Chr5.g19700.t1 | XP_015639433.1 | 4-coumarate--CoA ligase (EC 6.2.1.12) | A0A0E0PKM9 | 6.2.1.12 | phenylpropanoid metabolic process [GO:0009698] | GO:0004467; GO:0005783; GO:0009698; GO:0016020; GO:0016207; GO:0106290 | Metabolic pathways; Fatty acid metabolism; Fatty acid biosynthesis; Fatty acid degradation; Peroxisome; Ferroptosis; Quorum sensing; Adipocytokine signaling pathway; PPAR signaling pathway; Thermogenesis; |
| Chr6.g21650.t1 | XP_015642954.1 | Cytochrome P450 | A0A0E0PSB3 | - | flavonoid biosynthetic process [GO:0009813] | GO:0005506; GO:0009813; GO:0016020; GO:0016712; GO:0020037 | - |
| Chr6.g22055.t1 | XP_015643056.1 | Long-chain-fatty-acid--CoA ligase (EC 6.2.1.3) | B9FRL4 | 6.2.1.3 | phenylpropanoid metabolic process [GO:0009698] | GO:0004467; GO:0005524; GO:0009698; GO:0016207; GO:0106290 | Metabolic pathways; Fatty acid metabolism; Fatty acid biosynthesis; Fatty acid degradation; Peroxisome; Ferroptosis; Quorum sensing; Adipocytokine signaling pathway; PPAR signaling pathway; Thermogenesis; |
| Chr6.g25045.t1 | ACY70856.1 | 15-cis-phytoene synthase (EC 2.5.1.32) | A2YH75 | 2.5.1.32 | carotenoid biosynthetic process [GO:0016117] | GO:0004311; GO:0010287; GO:0016117; GO:0031969; GO:0046905; GO:0051996 | Metabolic pathways; Biosynthesis of secondary metabolites; Carotenoid biosynthesis; |
| Chr7.g26238.t1 | NP_001409028.1 | Bisdemethoxycurcumin synthase (EC 2.3.1.211) (Curcuminoid synthase) | Q8LIL0 | 2.3.1.211 | flavonoid biosynthetic process [GO:0009813]; polyketide biosynthetic process [GO:0030639] | GO:0009813; GO:0016747; GO:0030639; GO:0042802; GO:0102452 | Biosynthesis of secondary metabolites; Stilbenoid, diarylheptanoid and gingerol biosynthesis; |
| Chr7.g26295.t1 | NP_001409031.1 | 4-coumarate--CoA ligase-like 7 (EC 6.2.1.12) | Q69RG7 | 6.2.1.12 | phenylpropanoid metabolic process [GO:0009698] | GO:0005524; GO:0009698; GO:0016207; GO:0016405; GO:0106290 | Metabolic pathways; Biosynthesis of secondary metabolites; Ubiquinone and other terpenoid-quinone biosynthesis; Phenylpropanoid biosynthesis; |
| Chr7.g26296.t1 | NP_001409031.1 | 4-coumarate--CoA ligase-like 7 (EC 6.2.1.12) | Q69RG7 | 6.2.1.12 | phenylpropanoid metabolic process [GO:0009698] | GO:0005524; GO:0009698; GO:0016207; GO:0016405; GO:0106290 | Metabolic pathways; Biosynthesis of secondary metabolites; Ubiquinone and other terpenoid-quinone biosynthesis; Phenylpropanoid biosynthesis; |
| Chr7.g26496.t1 | XP_015646301.1 | Type III polyketide synthase 10 (OsPKS10) (EC 2.3.1.-) (Type III polyketide synthase 2) (OsPKS2) | Q8H305 | 2.3.1.- | flavonoid biosynthetic process [GO:0009813]; pollen wall assembly [GO:0010208]; polyketide biosynthetic process [GO:0030639]; sporopollenin biosynthetic process [GO:0080110] | GO:0005783; GO:0009813; GO:0010208; GO:0016747; GO:0030639; GO:0080110; GO:0090439 | - |
| Chr7.g27133.t1 | XP_015646281.1 | Chalcone synthase | A0A0E0I172 | - | flavonoid biosynthetic process [GO:0009813]; polyketide biosynthetic process [GO:0030639] | GO:0009813; GO:0016747; GO:0030639 | Biosynthesis of secondary metabolites; Stilbenoid, diarylheptanoid and gingerol biosynthesis; |
| Chr7.g27287.t1 | XP_015646364.1 | Os07g0525500 protein | Q0D5Y1 | - | flavonoid biosynthetic process [GO:0009813] | GO:0009813; GO:0016747 | Biosynthesis of secondary metabolites; Stilbenoid, diarylheptanoid and gingerol biosynthesis; |
| Chr7.g27291.t1 | XP_015646172.1 | Chalcone synthase | A0A0E0Q962 | - | flavonoid biosynthetic process [GO:0009813]; polyketide biosynthetic process [GO:0030639] | GO:0009813; GO:0016747; GO:0030639 | Biosynthesis of secondary metabolites; Stilbenoid, diarylheptanoid and gingerol biosynthesis; |
| Chr8.g28834.t1 | NP_001390411.1 | Uncharacterized protein | A0A0E0QEJ7 | - | flavonol biosynthetic process [GO:0051555]; lignin biosynthetic process [GO:0009809]; melatonin biosynthetic process [GO:0030187]; methylation [GO:0032259] | GO:0009809; GO:0017096; GO:0030187; GO:0032259; GO:0046983; GO:0051555; GO:0102822 | Metabolic pathways; Biosynthesis of secondary metabolites; Tryptophan metabolism; Phenylpropanoid biosynthesis; |
| Chr9.g32317.t1 | BAD36365.1 | Uncharacterized protein | Q69M32 | - | terpenoid biosynthetic process [GO:0016114] | GO:0010333; GO:0016114 | - |
| Chr9.g32651.t1 | XP_015612347.1 | 4-coumarate--CoA ligase (EC 6.2.1.12) | A0A0E0IIP2 | 6.2.1.12 | phenylpropanoid metabolic process [GO:0009698] | GO:0009698; GO:0016020; GO:0016207; GO:0106290 | - |
| Chr10.g34732.t1 | XP_015612714.1 | Flavonoid 3'-monooxygenase CYP75B4 (EC 1.14.14.82) (Chrysoeriol 5'-hydroxylase) (Cytochrome P450 75B4) (Flavonoid 3'-hydroxylase CYP75B4) | Q8LM92 | 1.14.14.82 | flavonoid biosynthetic process [GO:0009813] | GO:0005506; GO:0009813; GO:0016020; GO:0016711; GO:0020037 | - |
| Chr10.g34744.t1 | XP_015613041.1 | flavonoid 3'-monooxygenase (EC 1.14.14.82) | A0A0D3HC70 | 1.14.14.82 | flavonoid biosynthetic process [GO:0009813] | GO:0004497; GO:0005506; GO:0009813; GO:0016020; GO:0016705; GO:0020037 | Metabolic pathways; Biosynthesis of secondary metabolites; Flavonoid biosynthesis; Flavone and flavonol biosynthesis; |
| Chr10.g36154.t1 | XP_015614471.1 | Flavanone 3-dioxygenase 2 (EC 1.14.11.9) (Flavanone 3-beta-hydroxylase 2) (Flavanone 3-hydroxylase 2) (OsF3H-2) | Q8W2X5 | 1.14.11.9 | flavonoid biosynthetic process [GO:0009813] | GO:0009813; GO:0016706; GO:0031418; GO:0045486; GO:0046872 | - |
| Chr10.g36462.t1 | AAG46175.1 | 4-coumarate--CoA ligase-like 2 (EC 6.2.1.12) | Q336M7 | 6.2.1.12 | phenylpropanoid metabolic process [GO:0009698]; ubiquinone biosynthetic process [GO:0006744] | GO:0005524; GO:0005777; GO:0006744; GO:0009698; GO:0016207; GO:0016405; GO:0106290 | Metabolic pathways; Biosynthesis of secondary metabolites; Ubiquinone and other terpenoid-quinone biosynthesis; Phenylpropanoid biosynthesis; |
| Chr11.g36779.t1 | XP_015617433.1 | Long-chain-fatty-acid--CoA ligase (EC 6.2.1.3) | Q2RAL6 | 6.2.1.3 | phenylpropanoid metabolic process [GO:0009698] | GO:0004467; GO:0005524; GO:0005783; GO:0009698; GO:0016020; GO:0016207; GO:0106290 | Metabolic pathways; Fatty acid metabolism; Fatty acid biosynthesis; Fatty acid degradation; Peroxisome; Ferroptosis; Quorum sensing; Adipocytokine signaling pathway; PPAR signaling pathway; Thermogenesis; |
| Chr11.g38383.t1 | XP_015616270.1 | Chalcone/stilbene synthase N-terminal domain-containing protein | I1R0N1 | - | flavonoid biosynthetic process [GO:0009813]; polyketide biosynthetic process [GO:0030639] | GO:0009813; GO:0016747; GO:0030639 | Biosynthesis of secondary metabolites; Stilbenoid, diarylheptanoid and gingerol biosynthesis; |
| Chr11.g38384.t1 | XP_015616272.2 | Chalcone/stilbene synthase N-terminal domain-containing protein | A0A0E0R986 | - | flavonoid biosynthetic process [GO:0009813]; polyketide biosynthetic process [GO:0030639] | GO:0009813; GO:0016747; GO:0030639 | Biosynthesis of secondary metabolites; Stilbenoid, diarylheptanoid and gingerol biosynthesis; |
| Chr11.g38386.t1 | XP_052135962.1 | chalcone synthase (EC 2.3.1.74) | I1R0N5 | 2.3.1.74 | flavonoid biosynthetic process [GO:0009813]; polyketide biosynthetic process [GO:0030639] | GO:0009813; GO:0016747; GO:0030639 | Metabolic pathways; Biosynthesis of secondary metabolites; Flavonoid biosynthesis; Circadian rhythm - plant; |
| Chr11.g38598.t1 | XP_015616695.1 | Chalcone and stilbene synthases, N-terminal domain containing protein, expressed (Os11g0566800 protein) (cDNA clone:006-208-A07, full insert sequence) | Q2R2G0 | - | flavonoid biosynthetic process [GO:0009813] | GO:0009813; GO:0016747 | - |
| Chr12.g39794.t1 | XP_015620747.1 | 4-coumarate--CoA ligase (EC 6.2.1.12) | Q2QX58 | 6.2.1.12 | phenylpropanoid metabolic process [GO:0009698] | GO:0004467; GO:0005783; GO:0009698; GO:0016020; GO:0016207; GO:0106290 | Metabolic pathways; Fatty acid metabolism; Fatty acid biosynthesis; Fatty acid degradation; Peroxisome; Ferroptosis; Quorum sensing; Adipocytokine signaling pathway; PPAR signaling pathway; Thermogenesis; |
| Chr12.g41139.t1 | ABA98306.1 | Ent-kaurene synthase B, chloroplast, putative | Q2QQJ7 | - | terpenoid biosynthetic process [GO:0016114] | GO:0010333; GO:0016114 | - |
| Scaf_89.g42352.t1 | WP_028722639.1 | Phytoene desaturase (lycopene-forming) (EC 1.3.99.31) (4-step phytoene desaturase) (Phytoene dehydrogenase) | A0AAJ1CY22 | 1.3.99.31 | carotenoid biosynthetic process [GO:0016117] | GO:0016117; GO:0016627; GO:0071949 | - |

**Table: Abiotic stress responsive genes**

| **Gene ID** | RefSeq ID | **Protein Name** | Entry | EC number | **GO Biological Process** | **GO ID** | **KEGG Pathway** |
| --- | --- | --- | --- | --- | --- | --- | --- |
| Chr1.g265.t1 | NP_001388403.1 | SHSP domain-containing protein | A0A0D3EJB5 | - | response to heat [GO:0009408] | GO:0009408 | Protein processing in endoplasmic reticulum; |
| Chr1.g635.t1 | NP_001393167.1 | SHSP domain-containing protein | A0A0D3EKF4 | - | response to heat [GO:0009408] | GO:0005737; GO:0009408 | Protein processing in endoplasmic reticulum; |
| Chr2.g5500.t1 | XP_015626415.1 | Os02g0110400 protein (cDNA clone:J033023K10, full insert sequence) | Q6Z8Z5 | - | response to cold [GO:0009409] | GO:0003729; GO:0009409 | - |
| Chr2.g5527.t1 | XP_015625898.1 | F-box domain-containing protein | I1NWJ2 | - | response to cold [GO:0009409] | GO:0009409 | - |
| Chr2.g5624.t1 | NP_001396188.1 | SHSP domain-containing protein | A0A0E0G0J1 | - | response to heat [GO:0009408] | GO:0009408 | Protein processing in endoplasmic reticulum; |
| Chr2.g8445.t1 | XP_015622655.1 | Salt tolerance receptor-like cytoplasmic kinase 1 (EC 2.7.12.1) (Receptor-like cytoplasmic kinase 154) | A0A0E0HDU6 | 2.7.12.1 | response to cold [GO:0009409]; response to salt [GO:1902074]; response to water deprivation [GO:0009414] | GO:0004674; GO:0004713; GO:0005524; GO:0005886; GO:0009409; GO:0009414; GO:1902074 | - |
| Chr2.g8526.t1 | NP_001388845.1 | Trehalose 6-phosphate phosphatase (EC 3.1.3.12) | A0A0E0GAQ9 | 3.1.3.12 | response to cold [GO:0009409]; response to salt stress [GO:0009651]; trehalose biosynthetic process [GO:0005992] | GO:0004805; GO:0005992; GO:0009409; GO:0009651 | Metabolic pathways; Biosynthesis of secondary metabolites; Starch and sucrose metabolism; |
| Chr2.g9174.t1 | ADE61246.1 | Annexin | A2X9Q4 | - | response to cold [GO:0009409]; response to heat [GO:0009408]; response to salt stress [GO:0009651]; response to water deprivation [GO:0009414] | GO:0001786; GO:0005509; GO:0005544; GO:0005737; GO:0005886; GO:0009408; GO:0009409; GO:0009414; GO:0009651 | - |
| Chr2.g9326.t1 | XP_052143193.1 | Heat shock 70 kDa protein, mitochondrial | I1P4R6 | - | response to heat [GO:0009408] | GO:0005524; GO:0005783; GO:0009408; GO:0051082; GO:0140662 | RNA degradation; |
| Chr3.g9824.t1 | XP_015628989.1 | Salt tolerance receptor-like cytoplasmic kinase 1 (EC 2.7.12.1) (Receptor-like cytoplasmic kinase 154) | Q8GZY0 | 2.7.12.1 | response to cold [GO:0009409]; response to salt [GO:1902074]; response to water deprivation [GO:0009414]; signal transduction [GO:0007165] | GO:0004672; GO:0004674; GO:0004713; GO:0005524; GO:0005886; GO:0007165; GO:0009409; GO:0009414; GO:1902074 | - |
| Chr3.g10860.t1 | NP_001389056.1 | Uncharacterized protein | A0A0E0GKD0 | - | response to heat [GO:0009408] | GO:0009408 | Protein processing in endoplasmic reticulum; |
| Chr3.g10929.t1 | XP_015630538.1 | Uncharacterized protein | A0A0D3FGU6 | - | response to heat [GO:0009408]; response to virus [GO:0009615] | GO:0005524; GO:0009408; GO:0009615; GO:0140662 | Spliceosome; Protein processing in endoplasmic reticulum; MAPK signaling pathway; Endocytosis; Antigen processing and presentation; Longevity regulating pathway - multiple species; Legionellosis; Toxoplasmosis; Lipid and atherosclerosis; |
| Chr3.g11028.t1 | XP_015628701.1 | Os03g0289400 protein (Rhodanese-like family protein, putative) | Q10MY4 | - | response to cold [GO:0009409] | GO:0009409; GO:0016020 | - |
| Chr3.g14024.t1 | XP_015629763.1 | Uncharacterized protein | A0A0D3FR29 | - | response to cold [GO:0009409] | GO:0000166; GO:0003729; GO:0005634; GO:0005737; GO:0008270; GO:0009409 | - |
| Chr4.g16406.t1 | NP_001406291.1 | Uncharacterized protein | A0A0E0P9Q3 | - | response to heat [GO:0009408] | GO:0005783; GO:0009408 | Protein processing in endoplasmic reticulum; |
| Chr4.g17514.t1 | NP_001406777.1 | Calcium-transporting ATPase 5, plasma membrane-type (OsACA5) (EC 7.2.2.10) (Ca(2+)-ATPase isoform 5) (OsACA6) | Q7X8B5 | 7.2.2.10 | response to cold [GO:0009409]; response to water deprivation [GO:0009414] | GO:0005388; GO:0005516; GO:0005524; GO:0005886; GO:0009409; GO:0009414; GO:0016887; GO:0043231; GO:0046872 | - |
| Chr5.g18968.t1 | XP_015637534.1 | ABC transporter domain-containing protein | I1PTF8 | - | response to salt stress [GO:0009651] | GO:0005524; GO:0009651; GO:0016020; GO:0016887; GO:0140359 | ABC transporters; Bile secretion; Folate transport and metabolism; Antifolate resistance; |
| Chr5.g19588.t1 | XP_015639965.1 | Uncharacterized protein | I1PU83 | - | response to heat [GO:0009408] | GO:0005524; GO:0009408; GO:0051082; GO:0140662 | Spliceosome; Protein processing in endoplasmic reticulum; MAPK signaling pathway; Endocytosis; Antigen processing and presentation; Longevity regulating pathway - multiple species; Legionellosis; Toxoplasmosis; Lipid and atherosclerosis; |
| Chr5.g20139.t1 | XP_015640603.1 | Annexin | Q6L4C5 | - | response to cold [GO:0009409]; response to heat [GO:0009408]; response to salt stress [GO:0009651]; response to water deprivation [GO:0009414] | GO:0001786; GO:0005509; GO:0005544; GO:0005737; GO:0005886; GO:0009408; GO:0009409; GO:0009414; GO:0009651 | - |
| Chr5.g20941.t1 | NP_001407618.1 | 22.3 kDa class VI heat shock protein (22.3 kDa heat shock protein) (OsHsp22.3) | Q6AUW3 | - | response to heat [GO:0009408] | GO:0005737; GO:0009408 | - |
| Chr6.g22469.t1 | XP_052160360.1 | Annexin | I1Q0R9 | - | response to cold [GO:0009409]; response to heat [GO:0009408]; response to salt stress [GO:0009651]; response to water deprivation [GO:0009414] | GO:0001786; GO:0005509; GO:0005544; GO:0005737; GO:0005886; GO:0009408; GO:0009409; GO:0009414; GO:0009651 | - |
| Chr6.g23092.t1 | XP_015644245.1 | Selenoprotein O | Q5ZA96 | - | response to water deprivation [GO:0009414] | GO:0005524; GO:0009414; GO:0009534; GO:0046872; GO:0070733 | - |
| Chr7.g25074.t1 | XP_015646591.1 | BTB domain-containing protein | A0A0D3GL87 | - | cellular response to salt stress [GO:0071472]; protein ubiquitination [GO:0016567]; regulation of protein ubiquitination [GO:0031396] | GO:0005634; GO:0005829; GO:0016567; GO:0031396; GO:0042802; GO:0046872; GO:0071472 | Hedgehog signaling pathway; |
| Chr7.g25501.t1 | NP_001408921.1 | BTB domain-containing protein | A0A0E0Q4I8 | - | cellular response to salt stress [GO:0071472]; protein ubiquitination [GO:0016567] | GO:0016567; GO:0046872; GO:0071472 | Hedgehog signaling pathway; |
| Chr9.g33119.t1 | XP_015611676.1 | Annexin | I1QPH0 | - | response to cold [GO:0009409]; response to heat [GO:0009408]; response to salt stress [GO:0009651]; response to water deprivation [GO:0009414] | GO:0001786; GO:0005509; GO:0005544; GO:0005737; GO:0005886; GO:0009408; GO:0009409; GO:0009414; GO:0009651 | - |
| Chr9.g33363.t1 | XP_052167563.1 | Heat shock 70 kDa protein, mitochondrial | I1QRY4 | - | response to heat [GO:0009408] | GO:0005524; GO:0005783; GO:0009408; GO:0051082; GO:0140662 | RNA degradation; |
| Chr12.g39886.t1 | NP_001411447.1 | Thioredoxin domain-containing protein | A0A0E0J7K4 | - | hydrogen peroxide catabolic process [GO:0042744]; plastid organization [GO:0009657] | GO:0009507; GO:0009657; GO:0015035; GO:0042744 | NOD-like receptor signaling pathway; Salmonella infection; Fluid shear stress and atherosclerosis; |
| Chr12.g40185.t1 | XP_015619775.1 | Uncharacterized protein | A0A0E0RFH7 | - | response to heat [GO:0009408] | GO:0005524; GO:0009408; GO:0051082; GO:0140662 | Spliceosome; Protein processing in endoplasmic reticulum; MAPK signaling pathway; Endocytosis; Antigen processing and presentation; Longevity regulating pathway - multiple species; Legionellosis; Toxoplasmosis; Lipid and atherosclerosis; |

The annotation table includes several key fields that describe the biological characteristics of each gene. The gene ID represents the gene model identifier derived from the genome assembly. The RefSeq ID corresponds to the best protein match identified from the NCBI Reference Sequence database. UniProt accession numbers and entry names provide additional information regarding the protein identity and species homology. The protein name and gene symbol describe the predicted function of the gene based on sequence similarity. Enzyme Commission (EC) numbers indicate the biochemical reaction catalyzed by enzyme-encoding genes, while Gene Ontology terms provide functional classification in terms of biological processes, molecular functions, and cellular components. KEGG pathway annotations link genes to specific metabolic or signaling pathways, allowing functional interpretation of gene products within biological networks.
