# Supplementary material for: Genome sequencing and de novo assembly elucidate the genetic basis of grain quality and fragrance in heirloom rice cultivar Mushk Budji
Source: Front Plant Sci. 2026 Oct 14;17:1896265. doi: 10.3389/fpls.2026.1896265 (PMC13713480; doi:10.3389/fpls.2026.1896265)
Supplement: Supplementary file 3 [file Table5.docx]

**Genome sequencing and *de novo* assembly elucidate the genetic basis of grain quality and fragrance in heirloom** **rice cultivar *Mushk Budji***

**Raheel Shafeeq Khan^1^, Noor-ul-Ain^1^, V. Shasi Kiran Reddy^1^, Ishrat Rafiq^1^, Gazala H. Khan^2^, Fayaz Mohiddin^2^, Sajad M. Zargar^3^, Syed Zameer Hussain^4^, Bashir A. Alie^5^, Najeeb-ul-Rehman Sofi^2^, Asif Bashir Shikari^1^**

^1^Division of Genetics & Plant Breeding, Sher-e-Kashmir University of Agricultural Sciences & Technology of Kashmir, Wadura Campus, J&K, India, 193201

^2^Mountain Research Centre for Field Crops, Sher-e Kashmir University of Agricultural Sciences and Technology of Kashmir, Khudwani Campus, J&K, India, 192101

^3^Division of Biotechnology, Sher-e Kashmir University of Agricultural Sciences and Technology of Kashmir, Shalimar Campus, J&K, India, 190025

^4^Division of Food Science and Technology, Sher-e Kashmir University of Agricultural Sciences and Technology of Kashmir, Shalimar Campus, J&K, India, 190025

^5^Division of Agronomy, Sher-e Kashmir University of Agricultural Sciences and Technology of Kashmir, Wadura Campus, J&K, India, 193201

Corresponding author: [asifshikari@skuastkashmir.ac.in](mailto:asifshikari@skuastkashmir.ac.in)

**Supplementary Table S5. Imputed haplotype matrix of genes related to rice quality**

| **Gene** | **Hap_ID** | **REF**  **(Nipponbare)** | **Hap_1** | **Hap_2** | **Hap_3** | **Hap_4** | **Hap_5** | **Hap_6** |
| --- | --- | --- | --- | --- | --- | --- | --- | --- |
| **Os06g0229800 (*Alk*)** | **6748824** | **C** | **C** | **C** | **G** | **G** | **G** | ***C**** |
|  | **6750630** | **G** | **G** | **G** | **A** | **G** | **G** | **G** |
|  | **6751562** | **G** | **G** | **G** | **G** | **T** | **G** | **G** |
|  | **6752357** | **A** | **A** | **A** | **G** | **G** | ***A**** | **G** |
|  | **6752756** | **A** | **G** | **G** | **G** | **G** | **G** | **G** |
|  | **6752888** | **C** | **C** | **T** | **C** | **C** | **C** | **C** |
| **Os08g0424500 (*Badh2*)** | **20380322** | **A** | **A** | **A** | **G** | **A** | **A** |  |
|  | **20382857** | **A** | **A** | **A** | **A** | **T** | **T** |  |
|  | **20382859** | **A** | **A** | **A** | **A** | **-** | ***-**** |  |
|  | **20382860** | **A** | **A** | **A** | **A** | **-** | ***-**** |  |
|  | **20382861** | **G** | **G** | **G** | **G** | **-** | ***-**** |  |
|  | **20382862** | **A** | **A** | **A** | **A** | **-** | ***-**** |  |
|  | **20382863** | **T** | **T** | **T** | **T** | **-** | ***-**** |  |
|  | **20382867** | **G** | **G** | **G** | **G** | **-** | ***-**** |  |
|  | **20382868** | **G** | **G** | **G** | **G** | **-** | ***-**** |  |
|  | **20382869** | **C** | **C** | **C** | **C** | **-** | ***-**** |  |
|  | **20383153** | **A** | **A** | **G** | **A** | **A** | **G** |  |
|  | **20384391** | **C** | **C** | **G** | **C** | **C** | **C** |  |
|  | **20385103** | **T** | **T** | **T** | **-** | **T** | **T** |  |
| **Os03g0407400 (*GS3*)** | **16734956** | **T** | **T** | **T** | **T** | **-** |  |  |
|  | **16734955** | **T** | **T** | **T** | **T** | **-** |  |  |
|  | **16734954** | **A** | **A** | **A** | **A** | **-** |  |  |
|  | **16734953** | **G** | **G** | **G** | **G** | **-** |  |  |
|  | **16733441** | **G** | **G** | **T** | ***G**** | **G** |  |  |
|  | **16730020** | **A** | **A** | **A** | **T** | ***A**** |  |  |
| **Os06g0160700 (*SSI*)** | **3086531** | **C** | **C** | **C** | **A** | **C** | **C** |  |
|  | **3086439** | **G** | **G** | **T** | **T** | **T** | **T** |  |
|  | **3086397** | **T** | **T** | **C** | **C** | ***T**** | **T** |  |
|  | **3086385** | **C** | **C** | **A** | **A** | ***C**** | ***A**** |  |
|  | **3086359** | **C** | **C** | **A** | **A** | ***A**** | ***A**** |  |
|  | **3086313** | **A** | **A** | **G** | **G** | **A** | ***G**** |  |
|  | **3086312** | **C** | **C** | **C** | **T** | **C** | **C** |  |
|  | **3086311** | **C** | **C** | **T** | **T** | **C** | ***T**** |  |
|  | **3083079** | **T** | **T** | **C** | ***C**** | **T** | **C** |  |
|  | **3082490** | **G** | **G** | **A** | **A** | **G** | **A** |  |
| **Os06g0133000 (*Wx*)** | **1765761** | **T** | **T** | **G** | **G** | **G** | **G** |  |
|  | **1767938** | **C** | **C** | **C** | **C** | **-** | **-** |  |
|  | **1768006** | **A** | **A** | **A** | **C** | **A** | **A** |  |
|  | **1768998** | **C** | **C** | **C** | **C** | **C** | **T** |  |

(*)- Imputed signal

**Supplementary Table S6. Imputed haplotype summary of genes related to rice quality**

| ***Gene*** | ***REF***  ***(Nipponbare)*** | **Total Count** | **Core Count** | **Imputed Count** | **Accessions** |
| --- | --- | --- | --- | --- | --- |
| **Os06g0229800 (*Alk*)** | Hap_1 | 8 | 3 | 5 | Katarni Rice, Mushkandi I, Mushq Budji, Begum*, Ram Hall*, Shalimar Sugandh-1*, Bata Baber*, Taraori Basmati* |
|  | Hap_2 | 3 | 1 | 2 | Sant Andrea, K-332*, Koshihikari* |
|  | Hap_3 | 6 | 3 | 3 | Karnal Local, Sadri Rice 1, Sonasal, Pusa Basmati 1121*, TYPE-3 Basmati*, Ranbir Basmati* |
|  | Hap_4 | 2 | 1 | 1 | GS-28, Bansali* |
|  | Hap_5 | 1 | 0 | 1 | Gobindobhog* |
|  | Hap_6 | 1 | 0 | 1 | IR64A* |
| **Os08g0424500 (*Badh2*)** | Hap_1 | 7 | 5 | 2 | Bata Baber, Begum, IR64A, K-332, Sant Andrea, Koshihikari*, Ram Hall* |
|  | Hap_2 | 1 | 1 | 0 | Bansali |
|  | Hap_3 | 2 | 1 | 1 | Mushkandi I, GS-28* |
|  | Hap_4 | 10 | 6 | 4 | Karnal Local, Ranbir Basmati, Sadri Rice 1, Shalimar Sugandh-1, Sonasal, Taraori Basmati, Gobindobhog*, Mushq Budji*, Pusa Basmati 1121*, Katarni Rice* |
|  | Hap_5 | 1 | 0 | 1 | TYPE-3 Basmati* |
| **Os03g0407400 (*GS3*)** | Hap_1  (Short Grained, except Taraori Basmati | 11 | 9 | 2 | Gobindobhog, K-332, Katarni Rice, Koshihikari, Mushkandi I, Mushq Budji, Ram Hall, Sonasal, Taraori Basmati, Bata Baber*, Begum* |
|  | Hap_2  (Long grained) | 8 | 7 | 1 | IR64A, Karnal Local, Pusa Basmati 1121, Ranbir Basmati, Sadri Rice 1, Sant Andrea, Shalimar Sugandh-1, TYPE-3 Basmati* |
|  | Hap_3 | 1 | 0 | 1 | Bansali* |
|  | Hap_4 | 1 | 0 | 1 | GS-28* |
| **Os06g0160700 (*SSI*)** | Hap_1 | 9 | 5 | 4 | Begum, K-332, Koshihikari, Mushq Budji, Sant Andrea, Bata Baber*, IR64A*, Mushkandi I*, Shalimar Sugandh-1* |
|  | Hap_2 | 9 | 4 | 5 | Karnal Local, Katarni Rice, Sadri Rice 1, Taraori Basmati, GS-28*, Gobindobhog*, Pusa Basmati 1121*, Sonasal*, TYPE-3 Basmati* |
|  | Hap_3 | 1 | 0 | 1 | Bansali* |
|  | Hap_4 | 1 | 0 | 1 | Ram Hall* |
|  | Hap_5 | 1 | 0 | 1 | Ranbir Basmati* |
| **Os06g0133000 (*Wx*)** | Hap_1 (*Wx^b^*) | 3 | 3 | 0 | K-332, Ram Hall, Sant Andrea |
|  | Hap_2 (*Wx^lv^*) | 1 | 1 | 0 | Begum |
|  | Hap_3 (*Wx^in^*) | 13 | 10 | 3 | Bata Baber, Gobindobhog, IR64A, Karnal Local, Koshihikari, Mushq Budji, Pusa Basmati 1121, Sadri Rice 1, Sonasal, TYPE-3 Basmati, Mushkandi I*, Ranbir Basmati*, Taraori Basmati* |
|  | Hap_4 (*Wx^lv^)* | 2 | 1 | 1 | Bansali, Katarni Rice* |
|  | Hap_5 (*Wx^a^*) | 2 | 1 | 1 | GS-28, Shalimar Sugandh-1* |

(*)- Imputed Count

**Core Count:** Number of accessions with definitive sequencing coverage across all analysed functional sites.

**Imputed Count:** Number of accessions where minor gaps or heterozygosity were resolved using the whole-gene genetic background.

**Supplementary Table 7. Haplotype-phenotype grouping of *Mushk Budji* and reference genotypes for *Wx* locus**

| **Haplotype** | **Amylose Content (%)** | **Amylose Content (%)**  **Haplotype average** | **Accession** |
| --- | --- | --- | --- |
| Hap_1 | 17.6 | 18.27 | K-332 |
| Hap_1 | 19.3 |  | Ram Hall |
| Hap_1 | 17.9 |  | Sant Andrea |
| Hap_2 | 20.2 | 20.21 | Begum |
| Hap_3 | 18.7 | 21.97 | Bata Baber |
| Hap_3 | 19.5 |  | Gobindobhog |
| Hap_3 | 23.9 |  | IR-64 |
| Hap_3 | 22.5 |  | Karnal Local |
| Hap_3 | 16.2 |  | Koshihikari |
| Hap_3 | 18.9 |  | Mushkandi I |
| Hap_3 | 17.6 |  | Mushk Budji |
| Hap_3 | 24.2 |  | Pusa Basmati 1121 |
| Hap_3 | 22.8 |  | Ranbir Basmati |
| Hap_3 | 22.5 |  | Sadri Rice 1 |
| Hap_3 | 19.7 |  | Sonasal |
| Hap_3 | 20.8 |  | Taraori Basmati |
| Hap_3 | 21.1 |  | Type-3 Basmati |
| Hap_4 | 22.8 | 22.85 | Bansali |
| Hap_4 | 22.9 |  | Katarni Rice |
| Hap_5 | 21.0 | 22.70 | GS-28 |
| Hap_5 | 24.4 |  | Shalimar Sugandh 1 |

**Supplementary Table 8. Haplotype-phenotype grouping of *Mushk Budji* and reference genotypes for *GS3* locus**

| **Haplotype** | **Grain Type** | **Accession** |
| --- | --- | --- |
| Hap_1 | Short-bold | Bata Baber |
| Hap_1 | Short-bold | Begum |
| Hap_1 | Short-medium | Gobindobhog |
| Hap_1 | Short-bold | K-332 |
| Hap_1 | Short-medium | Katarni Rice |
| Hap_1 | Short-bold | Koshihikari |
| Hap_1 | Short-bold | Mushkandi I |
| Hap_1 | Short-bold | Mushk Budji |
| Hap_1 | Medium-slender | Ram Hall |
| Hap_1 | Short-bold | Sonasal |
| Hap_1 | Long-slender | Taraori Basmati |
| Hap_2 | Long-slender | IR-64 |
| Hap_2 | Long-slender | Karnal Local |
| Hap_2 | Long-slender | Pusa Basmati 1121 |
| Hap_2 | Long-slender | Ranbir Basmati |
| Hap_2 | Long-slender | Sadri Rice 1 |
| Hap_2 | Medium-bold | Sant Andrea |
| Hap_2 | Long-slender | Shalimar Sugandh-1 |
| Hap_2 | Long-slender | Type-3 Basmati |
| Hap_3 | Medium-bold | Bansali |
| Hap_4 | Short-bold | GS-28 |

**Supplementary Table 9. Haplotype-phenotype grouping of *Mushk Budji* and reference genotypes for *Badh2* locus**

| **Haplotype** | **Aroma** | **Accession** |
| --- | --- | --- |
| Hap_1 | Non-aromatic | Bata Baber |
| Hap_1 | Non-aromatic | Begum |
| Hap_1 | Non-aromatic | IR-64 |
| Hap_1 | Non-aromatic | K-332 |
| Hap_1 | Non-aromatic | Koshihikari |
| Hap_1 | Non-aromatic | Ram Hall |
| Hap_1 | Non-aromatic | Sant Andrea |
| Hap_2 | Non-aromatic | Bansali |
| Hap_3 | Non-aromatic | GS-28 |
| Hap_3 | Non-aromatic | Mushkandi I |
| Hap_4 | Aromatic | Gobindobhog |
| Hap_4 | Aromatic | Karnal Local |
| Hap_4 | Aromatic | Katarni Rice |
| Hap_4 | Aromatic | Mushk Budji |
| Hap_4 | Aromatic | Pusa Basmati 1121 |
| Hap_4 | Aromatic | Ranbir Basmati |
| Hap_4 | Aromatic | Sadri Rice 1 |
| Hap_4 | Aromatic | Shalimar Sugandh-1 |
| Hap_4 | Aromatic | Sonasal |
| Hap_4 | Aromatic | Taraori Basmati |
| Hap_5 | Aromatic | TYPE-3 Basmati |

**Supplementary Table S10. Population parameters of genes related to rice quality at evolutionary scale**

| **Category** | **Parameter** | **Sub-category** | **Os06g0229800** | **Os08g0424500** | **Os03g0407400** | **Os06g0160700** | **Os06g0133000** |
| --- | --- | --- | --- | --- | --- | --- | --- |
| **Nucleotide Diversity (π)** | **Overall Gene** | Length (bp) | 4905 | 6153 | 5609 | 7513 | 5032 |
|  |  | Variant Sites | 35 | 32 | 25 | 110 | 37 |
|  |  | π | 0.002510 | 0.001170 | 0.000804 | 0.006825 | 0.002003 |
|  | **CDS** | Length (bp) | 2433 | 1512 | 699 | 1926 | 1830 |
|  |  | Variant Sites | 8 | 1 | 2 | 21 | 4 |
|  |  | π | 0.001205 | 0.000336 | 0.000906 | 0.004962 | 0.000623 |
|  | **Intron** | Length (bp) | 1987 | 4348 | 4664 | 4890 | 2779 |
|  |  | Variant Sites | 24 | 30 | 23 | 87 | 33 |
|  |  | π | 0.003960 | 0.001468 | 0.000831 | 0.008336 | 0.003217 |
|  | **UTR** | Length (bp) | 485 | 293 | 246 | 697 | 344 |
|  |  | Variant Sites | 3 | 1 | 0 | 2 | 0 |
|  |  | π | 0.003110 | 0.001054 | 0 | 0.001371 | 0 |
| **Selection Pressure (Ka/Ks)** | **Ka (dN)** | — | 0.002175 | 0.000867 | 0.003711 | 0.004753 | 0.001424 |
|  | **Ks (dS)** | — | 0.006799 | 0 | 0 | 0.031871 | 0.004732 |
|  | **Ka/Ks Ratio** | — | 0.319860 | 0 | 0 | 0.149141 | 0.300901 |
|  | **Interpretation** | — | Moderate purifying selection (functional constraint) | No synonymous substitutions detected; ratio undefined but suggests positive selection | No synonymous substitutions detected; ratio undefined but suggests positive selection | Strong purifying selection; protein highly conserved | Moderate purifying selection; functional constraint |
| **Neutrality Test** | **Tajima’s D** | — | 1.702900 | -0.118200 | -0.787700 | 3.575900 | 0.509900 |
|  | **Interpretation** | — | Moderately positive (balancing selection or population) | Near neutral | Near neutral | Significantly positive; Strong Balancing selection | Near neutral |
| **Variant Quality** | **Ti/Tv Ratio** | — | 1.652000 | 1.400000 | 2.250000 | 1.681000 | 1.471000 |
|  | **Interpretation** | — | Acceptable; variant calls are generally reliable | Low; possible transversion artifacts | Excellent; consistent with expected biological ratio | Acceptable | Low; possible transversion artifacts |
