# Supplementary material for: Genome sequencing and de novo assembly elucidate the genetic basis of grain quality and fragrance in heirloom rice cultivar Mushk Budji
Source: Front Plant Sci. 2026 Oct 14;17:1896265. doi: 10.3389/fpls.2026.1896265 (PMC13713480; doi:10.3389/fpls.2026.1896265)
Supplement: Supplementary file 4 [file Table6.docx]

**Genome sequencing and *de novo* assembly elucidate the genetic basis of grain quality and fragrance in heirloom** **rice cultivar *Mushk Budji***

**Raheel Shafeeq Khan^1^, Noor-ul-Ain^1^, V. Shasi Kiran Reddy^1^, Ishrat Rafiq^1^, Gazala H. Khan^2^, Fayaz Mohiddin^2^, Sajad M. Zargar^3^, Syed Zameer Hussain^4^, Bashir A. Alie^5^, Najeeb-ul-Rehman Sofi^2^, Asif Bashir Shikari^1^**

^1^Division of Genetics & Plant Breeding, Sher-e-Kashmir University of Agricultural Sciences & Technology of Kashmir, Wadura Campus, J&K, India, 193201

^2^Mountain Research Centre for Field Crops, Sher-e Kashmir University of Agricultural Sciences and Technology of Kashmir, Khudwani Campus, J&K, India, 192101

^3^Division of Biotechnology, Sher-e Kashmir University of Agricultural Sciences and Technology of Kashmir, Shalimar Campus, J&K, India, 190025

^4^Division of Food Science and Technology, Sher-e Kashmir University of Agricultural Sciences and Technology of Kashmir, Shalimar Campus, J&K, India, 190025

^5^Division of Agronomy, Sher-e Kashmir University of Agricultural Sciences and Technology of Kashmir, Wadura Campus, J&K, India, 193201

Corresponding author: [asifshikari@skuastkashmir.ac.in](mailto:asifshikari@skuastkashmir.ac.in)

**Supplementary Figure S1. BUSCO Genome completeness assessment (N=425)**

**Supplementary Figure S2. KEGG Pathway distribution in Mushk Budji**

**Supplementary Figure S3. Genome-wide distribution and density of SNPs and InDels based on 10 kb sliding window analysis**
